# Phase Portraits of a Class of Continuous Piecewise Linear Differential Systems

**DOI:** 10.1007/s12591-023-00666-7

**Published:** 2023-11-30

**Authors:** Jie Li, Jaume Llibre

**Affiliations:** 1 School of Sciences, Southwest Petroleum University, Chengdu, 610500 Sichuan People’s Republic of China; 2 Departament de Matemàtiques, Universitat Autònoma de Barcelona, 08193 Bellaterra, Barcelona, Catalonia Spain

**Keywords:** Continuous piecewise linear differential system, Phase portrait, Limit cycle, Poincaré disc, 34A36, 34C07, 37G05

## Abstract

The phase portraits of the planar linear differential systems are very well known. This is not the case for the phase portraits of the planar continuous piecewise linear differential systems. In this paper we classify the phase portraits of the class of planar continuous piecewise linear differential systems of the form

$$\begin{aligned} {\dot{x}}= a|x|+by+c,\qquad {\dot{y}}= \alpha |x|+\beta y+\gamma , \end{aligned}$$

in the Poincaré disc when $a\beta -b\alpha \ne 0$, and prove the existence and uniqueness of limit cycles. Note that on the straight line $x=0$ these differential systems are only continuous.

## Introduction and Statement of the Main Result

Andronov et al. [1] started to study the piecewise linear differential systems in the 1920s for modelizing some mechanical systems, but the interest on this kind of differential systems persists up to nowadays. During the past twenty years many authors studied the dynamics of the piecewise linear differential systems, which can model many problems of mechanics, electronics, economy more accurately, see for instance [3, 4, 14, 18].

While the phase portraits of the linear differential systems

$$\begin{aligned} {{\dot{x}}}= ax+by+c,\qquad {{\dot{y}}}= \alpha x+\beta y+\gamma , \end{aligned}$$

are very well known, the phase portraits of the most easiest class of continuous piecewise linear differential systems

1 $$\begin{aligned} {{\dot{x}}}= a|x|+by+c,\qquad {{\dot{y}}}= \alpha |x|+\beta y+\gamma , \end{aligned}$$

with $a\beta -b\alpha \ne 0$ separated by the straight line $x=0$ are unknown. As usual the dot denotes derivative with respect to the independent variable of the differential systems, here called the time *t*. Note that these piecewise linear differential systems are analytic in ${{\mathbb {R}}}^2 \setminus \{x=0\}$ and only continuous on the straight line $x=0$. Of course the domain of definition of the continuous piecewise linear differential systems (1) is the whole plane ${{\mathbb {R}}}^2$.

The objective of this paper is to classify all topologically distinct phase portraits of the differential systems (1) in the Poincaré disc.

Recall that the *phase portrait* of a differential system is the description of the domain of definition of the differential system as union of all their orbits, in this way we know where the orbits born and die (i.e., their $\alpha$-limits and $\omega$-limits), and where equilibrium points, periodic orbits and homoclinic orbits are, ..., of course if these kinds of orbits exist. In other words the phase portrait of a differential system provides all the qualitative information about the dynamics of a differential system. There are some new results about planar continuous piecewise linear differential systems with two pieces separated by a straight line, such as [9, 10], and some references therein.

A phase portrait in the Poincaré disc has the advantage with respect to a phase portrait in the plane ${{\mathbb {R}}}^2$ that it controls the orbits which come from or escape to the infinity. Roughly speaking the Poincaré disc ${{\mathbb {D}}}$ is the closed disc of radius one centered at the origin of coordinates whose interior has been identified with ${{\mathbb {R}}}^2$ and its boundary, the circle ${{\mathbb {S}}}^1$, with the infinity of ${{\mathbb {R}}}^2$. For more details in the Poincaré disc see “Poincaré Compactification” section.

A periodic orbit isolated in the set of all periodic orbits of systems (1) is called a limit cycle.

Our main result is the following one.

### Theorem 1

**Fig. 1:**
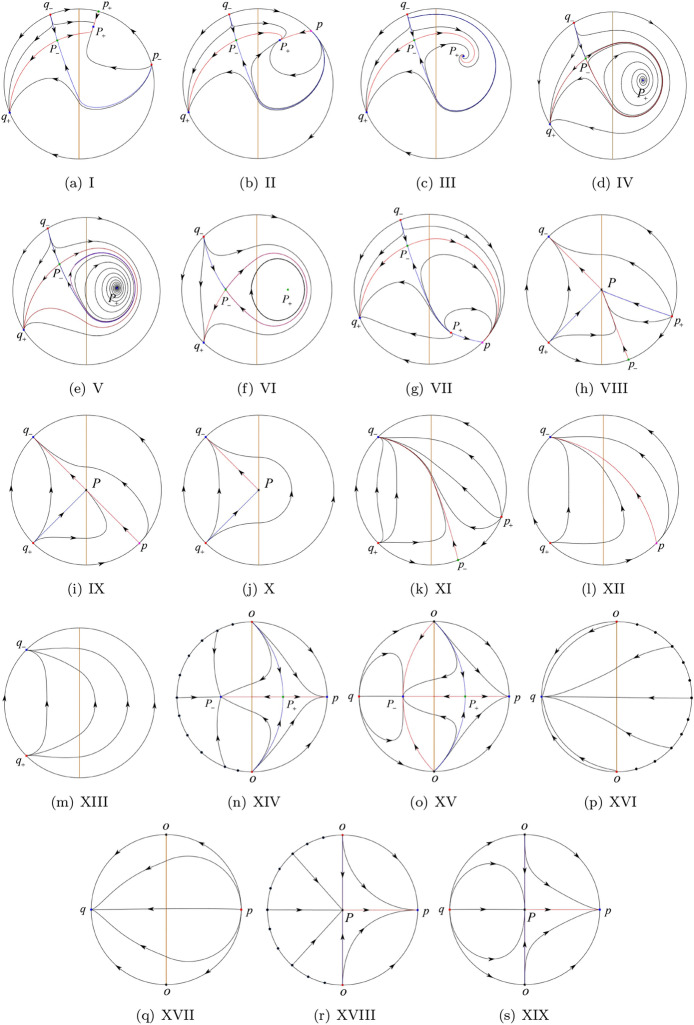
The phase portrait in the Poincaré disc of a continuous piecewise differential system (1) is topologically equivalent to one of the XIX phase portraits

The phase portrait in the Poincaré disc of a continuous piecewise linear differential system (1) is topologically equivalent to one of the XIX phase portraits described in Fig. 1. Moreover, there exists a limit cycle in Figs. 21 and 25.

Theorem 1 is proved in Sects. 3 and 4.

## Preliminaries

### The Normal Forms of the Differential Systems (1)

The continuous piecewise linear differential systems (1) depend on six parameters, but we will see that only two parameters are essential.

Since *b* and $\beta$ cannot be zero simultaneously, first we can assume that $b\ne 0$. Inspired in Proposition 3.1 of [7] we do the diffeomorphism $h:{{\mathbb {R}}}^2 \rightarrow {{\mathbb {R}}}^2$ defined by $h(x,y)= (x,\beta x-b y-c)=(X,Y)$, which transforms systems (1) into the continuous piecewise linear differential systems

2 $$\begin{aligned} \quad \,{{\dot{X}}}= {{\bar{a}}} X-Y, \qquad {{\dot{Y}}}= {{\bar{d}}} X+{{\bar{c}}}, \qquad \text{ if } X\ge 0\text{, } \text{ and } \end{aligned}$$

3 $$\begin{aligned} {{\dot{X}}}= {{\bar{b}}} X-Y, \qquad {{\dot{Y}}}= -{{\bar{d}}} X+{{\bar{c}}}, \quad \text{ if } X\le 0, \end{aligned}$$

where $${{\bar{a}}}=\beta +a$$, $${{\bar{b}}}=\beta -a$$, $${{\bar{c}}}=c\beta -b\gamma$$ and $$\bar{d}=a\beta -b\alpha \ne 0$$.

If ${{\bar{c}}}=0$, then doing the rescaling $(X,Y,t)= (x/|{\bar{d}}|,y,{{\bar{t}}} /|{\bar{d}}|)$ systems (2) and (3) become

4 $$\begin{aligned} \qquad {{\dot{x}}}= {{\hat{a}}} x-y, \qquad {{\dot{y}}}= \pm x, \quad \text{ if } x\ge 0\text{, } \text{ and } \end{aligned}$$

5 $$\begin{aligned} {{\dot{x}}}= {{\hat{b}}} x-y, \qquad {{\dot{y}}}= {\mp } x, \quad \text{ if } x\le 0, \end{aligned}$$

where ${{\hat{a}}}= {{\bar{a}}}/|{{\bar{d}}}|$, ${{\hat{b}}}= {{\bar{b}}}/|{{\bar{d}}}|$, now the dot denotes derivative with respect to the new time ${{\bar{t}}}$, the upper sign takes place when ${{\bar{d}}}>0$, and the lower sign takes place when ${{\bar{d}}}<0$. Note that by exchanging the position of ${\hat{a}}$ and ${{\hat{b}}}$ systems (4) with upper sign are the same that systems (5) with lower sign, while systems (4) with lower sign are the same that systems (5) with upper sign.

Now we further do the rescaling $(X,Y,t)=({{\bar{c}}} {{\bar{x}}}/{{\bar{d}}}, \bar{c} {{\bar{y}}}/\sqrt{|{{\bar{d}}}|}, {{\bar{t}}}/\sqrt{|{{\bar{d}}}|})$ if ${{\bar{c}}} \bar{d}>0$ and systems (2) and (3) become

6 $$\begin{aligned} \qquad \,\dot{{{\bar{x}}}}= {{\tilde{a}}} {{\bar{x}}}\pm {{\bar{y}}}, \qquad \dot{{{\bar{y}}}}= {{\bar{x}}}+1, \qquad \text{ if } {{\bar{x}}}\ge 0\text{, } \text{ and } \end{aligned}$$

7 $$\begin{aligned} \dot{{{\bar{x}}}}= {{\tilde{b}}} {{\bar{x}}}\pm {{\bar{y}}}, \qquad \dot{{{\bar{y}}}}= -\bar{x}+1, \quad \text{ if } {{\bar{x}}}\le 0, \end{aligned}$$

where ${{\tilde{a}}}= {{\bar{a}}}/\sqrt{|{{\bar{d}}}|}$, ${{\tilde{b}}}= \bar{b}/\sqrt{|{{\bar{d}}}|}$, and now the dot denotes derivative with respect to the new time ${{\bar{t}}}$. Moreover, if ${{\bar{d}}}>0$ then the signs in (6) and (7) are negative, otherwise they are positive. When ${{\bar{c}}}{{\bar{d}}}<0$, using the rescaling $(X,Y,t)=(-\bar{c} {{\bar{x}}}/{{\bar{d}}}, {{\bar{c}}} {{\bar{y}}}/\sqrt{|{{\bar{d}}}|}, {{\bar{t}}}/\sqrt{|\bar{d}|})$, we change systems (2) and (3) to the following

8 $$\begin{aligned} \qquad \dot{{{\bar{x}}}}= {{\tilde{a}}} {{\bar{x}}}\pm {{\bar{y}}}, \qquad \dot{{{\bar{y}}}}= -{{\bar{x}}}+1, \qquad \text{ if } {{\bar{x}}}\ge 0\text{, } \text{ and } \end{aligned}$$

9 $$\begin{aligned} \dot{{{\bar{x}}}}= {{\tilde{b}}} {{\bar{x}}}\pm {{\bar{y}}}, \qquad \dot{{{\bar{y}}}}= \bar{x}+1, \qquad \text{ if } {{\bar{x}}}\le 0. \end{aligned}$$

Similarly, if ${{\bar{d}}}>0$ then the signs in (8) and (9) are positive, otherwise they are negative. By $({\bar{x}},{\bar{y}},t)\rightarrow (-{\bar{x}},{\bar{y}},t)$, the negative situation is similar to the positive situation by exchanging the position of ${\tilde{a}}$ and ${{\tilde{b}}}$.

Assuming that $b=0$, we similarly do the diffeomorphism $h:{{\mathbb {R}}}^2 \rightarrow {{\mathbb {R}}}^2$ defined as $h(x,y)= (x,\beta y+\gamma )=(X,Y)$, which transforms systems (1) into the continuous piecewise linear differential systems

10 $$\begin{aligned} {{\dot{X}}}= aX+c, \qquad {{\dot{Y}}}=\beta (\alpha X+Y), \qquad \text{ if } X\ge 0\text{, } \text{ and } \end{aligned}$$

11 $$\begin{aligned} {{\dot{X}}}= -aX+c, \qquad {{\dot{Y}}}= \beta (-\alpha X+Y), \quad \text{ if } X\le 0. \end{aligned}$$

If $c=0$, then doing the rescaling $(X,Y,t)= (x,y,{{\bar{t}}} /|a|)$ systems (10) and (11) become

12 $$\begin{aligned} {{\dot{x}}}= \pm x, \qquad {{\dot{y}}}= {\check{a}} x+{\check{b}}y, \quad \text{ if } x\ge 0\text{, } \text{ and } \end{aligned}$$

13 $$\begin{aligned} {{\dot{x}}}= {\mp } x, \qquad {{\dot{y}}}= -{\check{a}} x+{\check{b}}y, \quad \text{ if } x\le 0, \end{aligned}$$

where ${\check{a}}= \alpha \beta /|a|$, ${\check{b}}= \beta /|a|$, now the dot denotes derivative with respect to the new time ${{\bar{t}}}$, the upper sign takes place when $a>0$, and the lower sign takes place when $a<0$. Note that $a\ne 0$, otherwise $a\beta -\alpha b=0$. On the other hand systems (12) with the lower sign are the same that systems (13) with the upper sign if we regard ${\check{a}}$ as $-{\check{a}}$. While systems (12) with the upper sign are also the same that systems (13) with the lower sign.

If $c\ne 0$, doing the rescaling $(X,Y,t)= (|c|x/|a|,y,{{\bar{t}}}/|a|)$ systems (10) and (11) become

14 $$\begin{aligned} \qquad \qquad S_+: {{\dot{x}}}= \pm x\pm 1, \qquad {{\dot{y}}}=\check{{\check{a}}} x+{\check{b}}y, \qquad \text{ if } x\ge 0\text{, } \text{ and } \end{aligned}$$

15 $$\begin{aligned} \qquad \qquad S_-: {{\dot{x}}}= {\mp } x\pm 1, \qquad {{\dot{y}}}=-\check{{\check{a}}} x+{\check{b}}y, \qquad \text{ if } x\le 0. \end{aligned}$$

where $\check{{\check{a}}}=\alpha \beta |c|/|a|^2$, ${\check{b}}=\beta /|a|$ and now the dot denotes derivative with respect to the new time ${{\bar{t}}}$. Note that the signs of *a* and *c* determine the signs in front of *x* and 1 respectively. More precisely, the upper signs takes place when $a>0$ and $c>0$ respectively, and the lower signs takes place when $a<0$ and $c<0$ respectively. For convenience we denote systems (14) by $S_+:(+,+), S_+:(+,-), S_+:(-,+), S_+:(-,-)$ and systems (15) by $S_-:(-,+), S_-:(-,-), S_-:(+,+), S_-:(+,-)$ according to the order of signs in front of *x* and 1. By $(x,y,\check{{\check{a}}},{\check{b}},t)\rightarrow (x,y,-\check{{\check{a}}},-{\check{b}},-t),$ systems $S_+:(+,+)$ and $S_+(+,-)$ (respectively $S_-(-,+)$ and $S_-(-,-)$) are changed to systems $S_+:(-,-)$ and $S_+:(-,+)$ (respectively $S_-(+,-)$ and $S_-(+,+)$). Thus we can obtain the 6 normal forms with only two parameters of the continuous piecewise differential systems (1) shown in Table 1, where we use $\sigma$ and $\varsigma$ as two new parameters for convenience. This completes the proof of Table 1.

In summary to classify the phase portraits of the continuous piecewise differential systems (1) is equivalent to classify the phase portraits of the continuous piecewise linear differential systems of Table 1. Note that the continuous piecewise linear differential systems of Table 1 only depend on two parameters.

**Table 1:** The 6 normal forms with only two parameters of the continuous piecewise differential systems (1)

$b\ne 0$	${{\bar{c}}}<0$	${{\bar{d}}}>0$	$S_+:{{\dot{x}}}= \sigma x+ y,\quad {{\dot{y}}}= -x+1$, if $x\ge 0$
			(I):
			$S_-:{{\dot{x}}}= \varsigma x+y, \quad {{\dot{y}}}= x+1$, if $x\le 0$
	${{\bar{c}}}=0$	${{\bar{d}}}>0$	$S_+:{{\dot{x}}}= \sigma x- y,\quad {{\dot{y}}}= x$, if $x\ge 0$
			(II):
			$S_-:{{\dot{x}}}=\varsigma x- y,\quad {{\dot{y}}}= -x$, if $x\le 0$
	${{\bar{c}}}>0$	${{\bar{d}}}>0$	$S_+:{{\dot{x}}}= \sigma x-y, \quad {{\dot{y}}}= x+1, \quad \text{ if } x\ge 0$
			(III):
			$$S_-:{{\dot{x}}}= \varsigma x- y, \quad {{\dot{y}}}= -x+1, \quad \text{ if } x\le 0$$
$b=0$	$c<0$	$a>0$	$S_+:{{\dot{x}}}= x- 1, \qquad {{\dot{y}}}=\sigma x+\varsigma y, \quad \text{ if } x\ge 0$
			(IV):
			$$S_-:{{\dot{x}}}= - x-1, \quad {{\dot{y}}}=-\sigma x+\varsigma y, \quad \text{ if } x\le 0,$$
		$a<0$	$S_+:{{\dot{x}}}= -x-1, \quad {{\dot{y}}}=\sigma x+\varsigma y, \quad \text{ if } x\ge 0$
			(V):
			$$S_-:{{\dot{x}}}= x-1, \qquad {{\dot{y}}}=-\sigma x+\varsigma y, \quad \text{ if } x\le 0,$$
	$c=0$	$a>0$	$S_+:{{\dot{x}}}= x, \qquad {{\dot{y}}}= \sigma x+\varsigma y, \quad \text{ if } x\ge 0$
			(VI):
			$$S_-:{{\dot{x}}}=-x, \quad {{\dot{y}}}= -\sigma x+\varsigma y, \quad \text{ if } x\le 0,$$

### Poincaré Compactification

In the proof of Theorem 1 we will use the Poincaré compactification of a planar polynomial vector field ${\mathcal {X}}(x,y)=(P(x,y),Q(x,y))$ of degree *d*. The *Poincaré compactification* of ${\mathcal {X}}$, denoted by $p({\mathcal {X}})$, is an induced vector field on the sphere ${{\mathbb {S}}}^2 =\{y=(y_1,y_2,y_3) \in {{\mathbb {R}}}^3: y_1^2 + y_2^2 + y_3^2=1\}$. We call ${{\mathbb {S}}}^2$ the *Poincaré sphere*. For more details on the Poincaré compactification see [5, Chapter 5]. Here we just introduce what we need.

Denote by $T_p {{\mathbb {S}}}^2$ be the tangent space to ${{\mathbb {S}}}^2$ at the point *p*. Assume that ${\mathcal {X}}$ is defined in the plane $T_{(0,0,1)} {{\mathbb {S}}}^2 ={{\mathbb {R}}}^2$. Consider the central projection $f :T_{(0,0,1)} {{\mathbb {S}}}^2 \rightarrow {{\mathbb {S}}}^2$. This map defines two copies of ${\mathcal {X}}$, one in the open northern hemisphere ${\mathcal {H}}^+$ and the other in the open southern hemisphere ${\mathcal {H}}^-$. Denote by ${\mathcal {X}}^{1}$ the vector field $Df \circ {\mathcal {X}}$ defined on ${{\mathbb {S}}}^2$ except on its equator ${{\mathbb {S}}}^1 =\{y \in {{\mathbb {S}}}^2 : y_3=0\}$. Clearly ${{\mathbb {S}}}^1$ is identified with the infinity of ${{\mathbb {R}}}^2$. In order to extend ${\mathcal {X}}^{1}$ to a vector field on ${{\mathbb {S}}}^2$ (including ${{\mathbb {S}}}^1$) it is necessary that ${\mathcal {X}}$ satisfies suitable conditions. In the case that ${\mathcal {X}}$ is a planar polynomial vector field of degree *d*, then $p({\mathcal {X}})$ is the only analytic extension $y_3^{d-1} {\mathcal {X}}^{1}$ to ${{\mathbb {S}}}^2$. On ${{\mathbb {S}}}^2 {\setminus } {{\mathbb {S}}}^1 = {\mathcal {H}}^+ \cup {\mathcal {H}}^-$ there are two symmetric copies of $p({\mathcal {X}})$, one in ${\mathcal {H}}^+$ and the other in ${\mathcal {H}}^-$, and knowing the behaviour of $p({\mathcal {X}})$ around ${{\mathbb {S}}}^1$, we know the behaviour of ${\mathcal {X}}$ at infinity. The Poincaré compactification has the property that ${{\mathbb {S}}}^1$ is invariant under the flow of $p({\mathcal {X}})$. The equilibrium points of ${\mathcal {X}}$ are called the *finite equilibrium points* of ${\mathcal {X}}$ or of $p({\mathcal {X}})$, while the equilibrium points of $p({\mathcal {X}})$ contained in ${{\mathbb {S}}}^1$, i.e. at infinity, are called the *infinite equilibrium points* of ${\mathcal {X}}$ or of $p({\mathcal {X}})$. It is known that the infinity equilibrium points appear in pairs diametrically opposed.

To study the vector field $p({\mathcal {X}})$ we use six local charts on ${{\mathbb {S}}}^2$ given by $U_k=\{y\in {{\mathbb {S}}}^2:\,y_k>0\}$, $V_k=\{y\in {{\mathbb {S}}}^2\,:\,y_k<0\}$ for $k=1,2,3$. The corresponding local maps $\phi _k:U_k\rightarrow {{\mathbb {R}}}^2$ and $\psi _k:V_k\rightarrow {{\mathbb {R}}}^2$ are defined as $\phi _k(y)=\psi _k(y)=(y_m/y_k,y_n/y_k)$ for $m<n$ and $m,n\ne k$. We denote by $z=(u,v)$ the value of $\phi _k(y)$ or $\psi _k(y)$ for any *k*, then (*u*, *v*) will play different roles depending on the local chart we are considering. The points of the infinity ${{\mathbb {S}}}^1$ in any chart have their coordinates $v=0$. The expression for $p({\mathcal {X}})$ in local chart $(U_1,\phi _1)$ is

$$\begin{aligned} {{\dot{u}}}=v^d \left[ -u P\left( \dfrac{1}{v},\dfrac{u}{v}\right) + Q\left( \dfrac{1}{v},\dfrac{u}{v}\right) \right] ,\quad {{\dot{v}}}=-v^{d+1} P\left( \dfrac{1}{v},\dfrac{u}{v}\right) , \end{aligned}$$

in the local chart $(U_2,\phi _2)$ is

$$\begin{aligned} {{\dot{u}}}=v^d \left[ -u Q\left( \dfrac{u}{v},\dfrac{1}{v}\right) + P\left( \dfrac{u}{v},\dfrac{1}{v}\right) \right] ,\quad {{\dot{v}}}=-v^{d+1} Q\left( \dfrac{u}{v},\dfrac{1}{v}\right) , \end{aligned}$$

and in the local chart $(U_3,\phi _3)$ is ${{\dot{u}}}=P(u,v),\quad {{\dot{v}}}=Q(u,v)$.

We note that the expression of the vector field $p({\mathcal {X}})$ in the local chart $(V_i,\psi _i)$ is equal to the expression in the local chart $(U_i, \phi _i)$ multiplied by $(-1)^{d-1}$ for $i=1,2,3$.

The orthogonal projection under $\pi (y_1,y_2,y_3)=(y_1,y_2)$ of the closed northern hemisphere of ${{\mathbb {S}}}^2$ onto the plane $y_3=0$ is a closed disc ${{\mathbb {D}}}$ of radius one centered at the origin of coordinates called the *Poincaré disc*. Since a copy of the vector field ${\mathcal {X}}$ on the plane ${{\mathbb {R}}}^2$ is in the open northern hemisphere of ${{\mathbb {S}}}^2$, the interior of the Poincaré disc ${{\mathbb {D}}}$ is identified with ${{\mathbb {R}}}^2$ and the boundary of ${{\mathbb {D}}}$, the equator of ${{\mathbb {S}}}^2$, is identified with the infinity of ${{\mathbb {R}}}^2$. Consequently the phase portrait of the vector field ${\mathcal {X}}$ extended to the infinity corresponds to the projection of the phase portrait of the vector field $p({\mathcal {X}})$ in the Poincaré disc ${{\mathbb {D}}}$.

By definition the *infinite equilibrium points* of the polynomial vector field ${\mathcal {X}}$ are the equilibrium points of $p({\mathcal {X}})$ contained in the boundary of the Poincaré disc, i.e. in ${{\mathbb {S}}}^1$, and the *finite equilibrium points* of ${\mathcal {X}}$ are the equilibrium points of $p({\mathcal {X}})$ contained in the interior of the Poincaré disc, which of course coincide with the equilibrium points of ${\mathcal {X}}$ in ${{\mathbb {R}}}^2$.

Note that for studying the infinite equilibrium points of the continuous piecewise differential systems (1) in $x\ge 0$ we only need to study the infinite equilibrium points which are in the local chart $U_1$ and to determine if the origin of the local chart $U_2$ is or not an equilibrium point. While for studying the infinite equilibrium points of the continuous piecewise differential systems (1) in $x\le 0$ we only need to study the infinite equilibrium points which are in the local chart $V_1$ and to determine if the origin of the local chart $U_2$ is or not an equilibrium point.

### Topological Equivalence of Two Polynomial Vector Fields

Let ${\mathcal {X}}_1$ and ${\mathcal {X}}_2$ be two polynomial vector fields on ${\mathbb {R}}^2$. We say that they are *topologically equivalent* if there exists a homeomorphism on the Poincaré disc ${{\mathbb {D}}}$ which preserves the infinity ${{\mathbb {S}}}^{1}$ and sends the orbits of $\pi (p({\mathcal {X}}_1))$ to orbits of $\pi (p({\mathcal {X}}_2))$, preserving or reversing the orientation of all the orbits.

A *separatrix* of the Poincaré compactification $\pi (p({\mathcal {X}}))$ is one of following orbits: all the orbits at the infinity ${{\mathbb {S}}}^1$, the finite equilibrium points, periodic orbits which are isolated in the set of periodic orbits at least by one side, when a periodic orbit is isolated in the set of periodic orbits by both sides it is a limit cycle, and the two orbits at the boundary of a hyperbolic sector at a finite or an infinite equilibrium point, see for more details on the separatrices [15, 16].

The set of all separatrices of $\pi (p({\mathcal {X}}))$, which we denote by $\Sigma _{\mathcal {X}}$, is a closed set (see [16]).

A *canonical region* of $\pi (p({\mathcal {X}}))$ is an open connected component of ${{\mathbb {D}}}\setminus \Sigma _{\mathcal {X}}$. The union of the set $\Sigma _{\mathcal {X}}$ with an orbit of each canonical region form the *separatrix configuration* of $\pi (p({\mathcal {X}}))$ and is denoted by $\Sigma '_{\mathcal {X}}$. We denote the number of separatrices of a phase portrait in the Poincaré disc by *S*, and its number of canonical regions by *R*.

Two separatrix configurations $\Sigma '_{{\mathcal {X}}_1}$ and $\Sigma '_{{\mathcal {X}}_2}$ are *topologically equivalent* if there is a homeomorphism $h:{{\mathbb {D}}}\longrightarrow {{\mathbb {D}}}$ such that $h(\Sigma '_{{\mathcal {X}}_1})=\Sigma '_{{\mathcal {X}}_2}$.

According to the following theorem which was proved by Markus [15], Neumann [16] and Peixoto [17], it is sufficient to investigate the separatrix configuration of a polynomial differential system, for determining its global phase portrait.

#### Theorem 2

Two Poincaré compactified polynomial vector fields $\pi (p({\mathcal {X}}_1))$ and $\pi (p({\mathcal {X}}_2))$ with finitely many separatrices are topologically equivalent if and only if their separatrix configurations $\Sigma '_{{\mathcal {X}}_1}$ and $\Sigma '_{{\mathcal {X}}_2}$ are topologically equivalent.

### Limit Cycles

In 1991–1992 Lum and Chua in [12, 13] conjectured that a continuous piecewise linear differential system in the plane with two pieces separated by one straight line has at most one limit cycle. We note that even in this apparent simple case, only after a difficult analysis it was possible to prove the existence of at most one limit cycle, thus in 1998 this conjecture was proved by Freire, Ponce, Rodrigo and Torres in [6]. Recently, a new easier proof that at most one limit cycle exists for the continuous piecewise linear differential systems with two pieces separated by one straight line has been done by Llibre et al. in [11]. For other results on the limit cycles of continuous and discontinuous piecewise differential systems see [2].

## Proof of Theorem 1

The continuous piecewise differential systems (1) with $a\beta -b\alpha \ne 0$ are topologically equivalent to some of the six continuous piecewise differential systems of Table 1.

If the *x*-coordinate of an equilibrium point is positive (respectively negative), this equilibrium point is *real* (respectively *virtual*) for the differential systems $S_+$. If the *x*-coordinate of an equilibrium point is negative (respectively positive), this equilibrium point is *real* (respectively *virtual*) for the differential systems $S_-$. Of course if the *x*-coordinate of an equilibrium point is zero, then this equilibrium point is real for both differential systems $S_+$ and $S_-$.

### Limit Cycles

For continuous piecewise linear differential systems (1), we study the existence and uniqueness of their limit cycles, see “Limit Cycles” section. Note that a region enclosed by a periodic orbit of systems (1) must contain equilibrium points and the sum of the indices of the equilibrium points in a region enclosed by any periodic orbit of systems (1) is one, as it is shown in the properties 2 and 3 of [19, p. 148].

#### Lemma 3

For continuous piecewise linear differential systems $\mathrm {(I)}$ there exists a unique limit cycle lying in the strip $-1<x<0$ if either $\varsigma <0$ and $0<\sigma <2$, or $\varsigma >0$ and $-2<\sigma <0$. Moreover, if $\varsigma =0$ and $\sigma =0$ there is a continuum of periodic orbits surrounding the equilibrium point $P_+$ of systems $S_+$ bounded by a homoclinic orbit connecting the saddle $P_-$ of systems $S_-$. And there is no periodic orbits for continuous piecewise linear differential systems $\mathrm {(II)}-\mathrm {(VI)}$

#### Proof

First we see that systems $S_-$ (respectively $S_+$) have the equilibrium point $P_-(-1,\varsigma )$ (respectively $P_+(1,-\sigma )$). Furthermore, the eigenvalues of the Jacobian matrix evaluated at $P_-$ are $(\varsigma \pm \sqrt{\varsigma ^{2}+4})/2$. The equilibrium point $P_-$ is a saddle whose index is $-1$, implying that there is no periodic orbits surrounding $P_-$ for systems (I) in the plane $x\le 1$.

The eigenvalues of the equilibrium point $P_+$ are $(\sigma \pm \sqrt{\sigma ^{2}-4})/2$. So if $\sigma \le -2$ (respectively $\sigma \ge 2$) then ${\lambda }_-\le {\lambda }_+<0$ (respectively ${\lambda }_+\ge {\lambda }_->0$) implying that $P_+$ is a stable (respectively an unstable) node. If $-2<\sigma <0$ (respectively $0<\sigma <2$) then ${\lambda }_\pm$ are a pair of imaginary eigenvalues with negative (respectively positive) real part, implying that $P_+$ is a stable (respectively an unstable) focus. If $\sigma =0$ then ${\lambda }_\pm$ are a pair of purely imaginary eigenvalues, implying that $P_+$ is a center. Thus the index of the equilibrium point $P_+$ is one.

On the other hand we see that the divergence of systems $S_+$ (respectively $S_-$) is $\sigma$ (respectively $\varsigma$). It implies by the Bendixson’s theorem [5, Theorem 7.10] that systems (I) have no periodic orbits surrounding $P_+$ when $\sigma \varsigma >0$.

When $\sigma \varsigma \le 0$ by the Bendixson’s theorem systems $S_+$ have no periodic orbits in the half plane $x\ge 0$ if $\sigma \ne 0$. Namely systems (I) have no periodic orbits in the half plane $x\ge 0$ if $\sigma \ne 0$.

When $\varsigma =0$ and $\sigma \ne 0$ by the Bendixson’s theorem systems $S_+$ have no periodic orbits in the half plane $x\ge -1$. Namely systems (I) have no periodic orbits in the half plane $x\ge -1$.

When $\sigma =0$ we define the two functions

$$\begin{aligned}{} & {} H_1(x,y)=(x - 1)^2 + y^2, \\{} & {} H_2(x,y)=\left( \frac{ (x+1)\sqrt{\varsigma ^2+4 } +\varsigma - \varsigma x - 2 y}{ (x+1)\sqrt{\varsigma ^2+4}-\varsigma +\varsigma x+2 y}\right) ^{\varsigma } (y^2 - (1 + x)^2 + \varsigma (x y - \varsigma x - y))^{\sqrt{4 + \varsigma ^2}}. \end{aligned}$$

We check $H_1$ (respectively $H_2$) is a first integral for systems $S_+$ (respectively $S_-$), i.e.,

$$\begin{aligned} (\partial H_{1}(x,y)/\partial x)y+(\partial H_{1}(x,y)/\partial y)(1-x)=0 \end{aligned}$$

(respectively $$(\partial H_{2}(x,y)/\partial x)(\varsigma x+y)+(\partial H_{2}(x,y)/\partial y)(x+1)=0$$). Compute

$$\begin{aligned} \lim \limits _{{x\rightarrow -1},{y\rightarrow \varsigma }} H_2(x,y)= 0. \end{aligned}$$

**Fig. 2:**
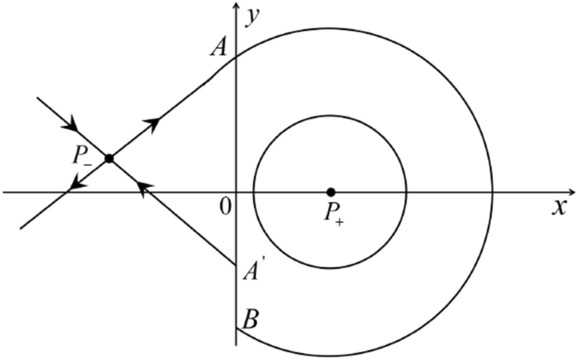
$$\varsigma >0$$

**Fig. 3:**
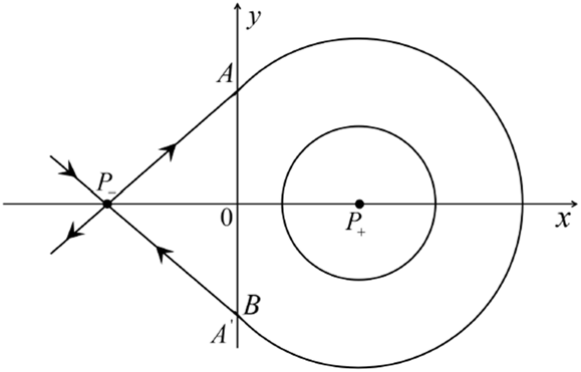
$$\varsigma =0$$

**Fig. 4:**
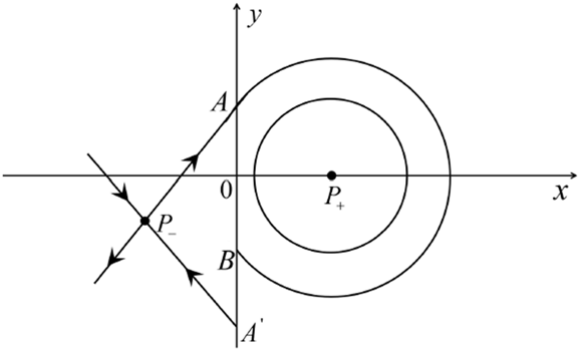
$$\varsigma <0$$

**Fig. 5:**
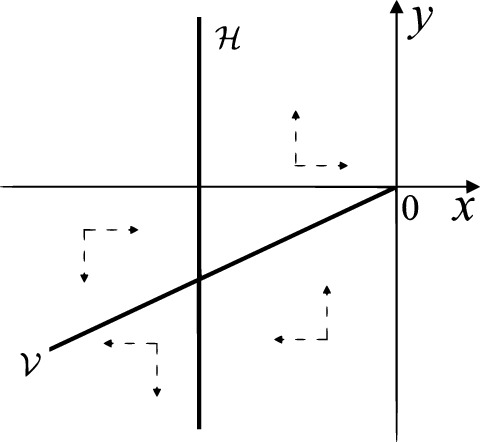
$$\varsigma <0$$

**Fig. 6:**
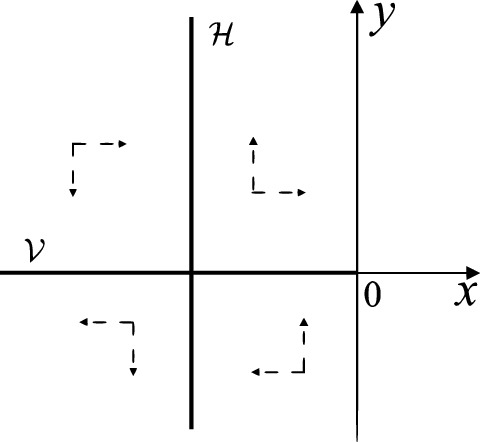
$$\varsigma =0$$

**Fig. 7:**
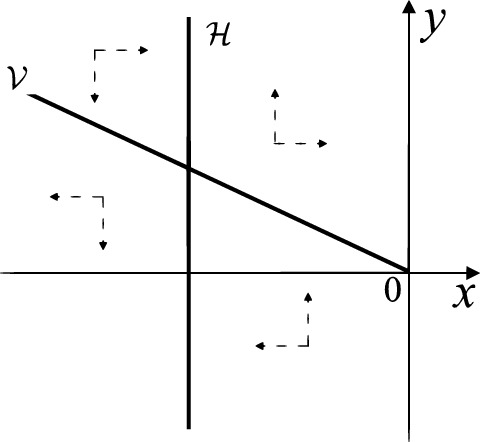
$$\varsigma >0$$

On the other hand for systems $S_-$ there are the horizontal isocline ${\mathcal {H}}: x=-1$ and the vertical isocline ${\mathcal {V}}: y=-\varsigma x$. More concretely, we see ${{\dot{y}}}>0$ on the right hand side of ${\mathcal {H}}$, and ${{\dot{y}}}<0$ on the left hand side of ${\mathcal {H}}$. And we get ${{\dot{x}}}>0$ in the upper of ${\mathcal {V}}$, and ${{\dot{x}}}<0$ in the lower of ${\mathcal {V}}$. So in the four regions divided by ${\mathcal {H}}$ and ${\mathcal {V}}$, the vector fields are shown in Figs. 2, 3, 4, 5, 6 and 7. Then one branch of the unstable manifold of the saddle $P_-$ intersects the positive *y*-axis at $A: (0,y_1)$, while one branch of the stable *y*-axis at $A': (0, y_1')$. Then $H_2(0,y_1)=H_2(0,y_1')=$ $\lim \limits _{{x\rightarrow -1},{y\rightarrow \varsigma }} H_2(x,y)=0$. Solving the equation $H_2(0,y)=0$, i.e.

$$\begin{aligned} \left( \frac{ \sqrt{4 + \varsigma ^2} +\varsigma - 2 y}{ \sqrt{4 + \varsigma ^2}-\varsigma + 2 y}\right) ^\varsigma (y^2 -\varsigma y-1)^{\sqrt{4 + \varsigma ^2}}=0, \end{aligned}$$

we get

$$\begin{aligned} y_1=\frac{1}{2} \left( \varsigma +\sqrt{\varsigma ^2+4}\right) ,\quad y_1'=\frac{1}{2} \left( \varsigma -\sqrt{\varsigma ^2+4}\right) . \end{aligned}$$

Note that some orbits of systems $S_+$ intersect *y*-axis and are symmetric with respect to the *x*-axis. Let $y_1=-y_1'$. Then $\varsigma =0$. Thus on the *y*-axis we look for the values of *y* such that $H_2(0,y)=H_2(0,-y)$. Namely

$$\begin{aligned} \left( \frac{ \sqrt{4 + \varsigma ^2} +\varsigma - 2 y}{ \sqrt{4 + \varsigma ^2}-\varsigma + 2 y}\right) ^\varsigma (y^2 -\varsigma y-1)^{\sqrt{4 + \varsigma ^2}}=\left( \frac{ \sqrt{4 + \varsigma ^2} +\varsigma + 2 y}{ \sqrt{4 + \varsigma ^2}-\varsigma - 2 y}\right) ^\varsigma (y^2 +\varsigma y-1)^{\sqrt{4 + \varsigma ^2}}. \end{aligned}$$

The equality holds when $\varsigma =0$. It implies that there are filled with periodic orbits inside the homoclinic orbit connecting to $P_-$, seeing Fig. 3. If $\varsigma >0$ then $y_1>y_1'$. One branch of the stable manifold of $P_-$ goes around the periodic orbit ${\mathcal {C}}:(x - 1)^2 + y^2=H_1(0,0)=1$. If $\varsigma <0$ then $y_1>y_1'$. One branch of the unstable manifold of $P_-$ goes around the periodic orbit ${\mathcal {C}}$. See Figs. 2 and 4.

**Fig. 8:**
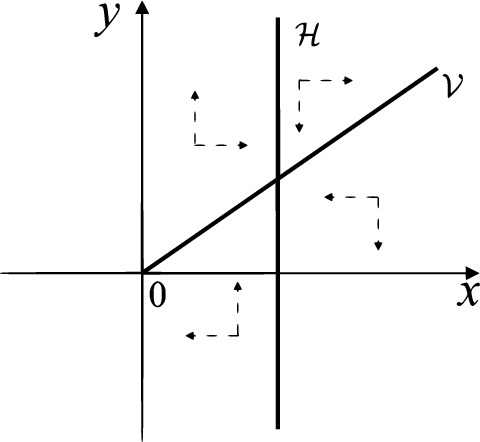
$$\sigma <0$$

**Fig. 9:**
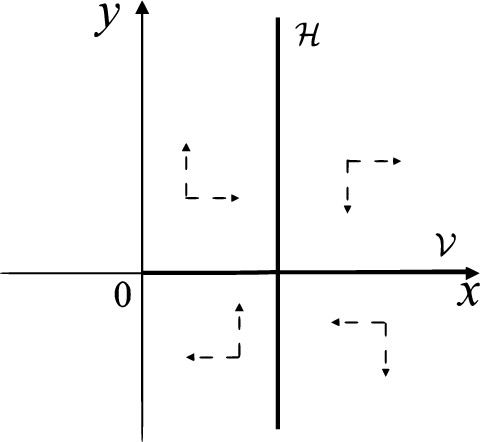
$$\sigma =0$$

**Fig. 10:**
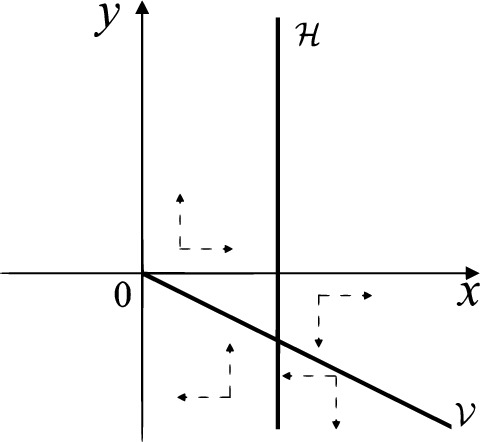
$$\sigma >0$$

When $\sigma \varsigma <0$ and either $\sigma \ge 2$ or $\sigma \le -2$ the equilibrium point $P_+$ is either an unstable node or a stable node. On the other hand for systems $S_+$ there are the horizontal isocline ${\mathcal {H}}: x=1$ and the vertical isocline ${\mathcal {V}}: y=-\sigma x$. More concretely, we see ${{\dot{y}}}<0$ on the right hand side of ${\mathcal {H}}$, and ${{\dot{y}}}>0$ on the left hand side of ${\mathcal {H}}$. And we get ${{\dot{x}}}>0$ in the upper of ${\mathcal {V}}$, and ${{\dot{x}}}<0$ in the lower of ${\mathcal {V}}$. So we see the vector fields in the four regions divided by ${\mathcal {H}}$ and ${\mathcal {V}}$ shown in Figs. 8, 9, and 10. There is an orbit linking $P_+$ with the infinity in the half plane $x\ge 1$. Thus there is no periodic orbits in the half plane $x\ge -1$.

When $\varsigma >0$ and $-2<\sigma <0$ let $M(0,\big (\varsigma +\sqrt{\varsigma ^2+4}\big )/2)$ (respectively $N(0,\big (\varsigma -\sqrt{\varsigma ^2+4}\big )/2)$) be the intersection point of the unstable (respectively stable) manifold of the saddle $P_-$ with the *y*-axis. Denote by $M_1(x_{M1},0)$ (respectively $N_1(x_{N1},0)$) the intersection point of the *x*-axis with the orbit starting at *M* (respectively *N*) if they intersect. When $\sigma =0$ we have the orbits living on the curves $H_1(x,y)=(x - 1)^2 + y^2=constant$ for $x\ge 0$, being symmetry with respect to *x*-axis. Then the points $M_1$ and $N_1$ exist with $x_{N1}<x_{M1}$. For $\sigma <0$ being small enough we see that $P_+$ is a stable focus and $x_{N1}<x_{M1}$ still holds as $\varsigma >0$. It follows from the Poincaré–Bendixson Theorem that there exists periodic orbits in the region surrounded by $P_-M\cup MM_1\cup M_1N_1 \cup N_1N\cup N P_-$. Furthermore we claim that there exists a value $\sigma _0$ such that $x_{N1}=x_{M1}$. This means that there exists a homoclinic orbit at $P_-$ and surrounding $P_+$. In fact when $\sigma =-2$ we write the differential systems $S_+$ in the local charts $U_1$ and $U_2$. Then in the local chart $U_1$ systems $S_+$ write

16 $$\begin{aligned} {{\dot{u}}}= -1+2 u+v-{u}^{2}, \quad {{\dot{v}}}= 2 v-uv; \end{aligned}$$

and in the local chart $U_2$ become

$$\begin{aligned} {{\dot{u}}}=1-2 u+{u}^{2}-uv, \quad {{\dot{v}}}= uv-{v}^{2}. \end{aligned}$$

**Fig. 11:**
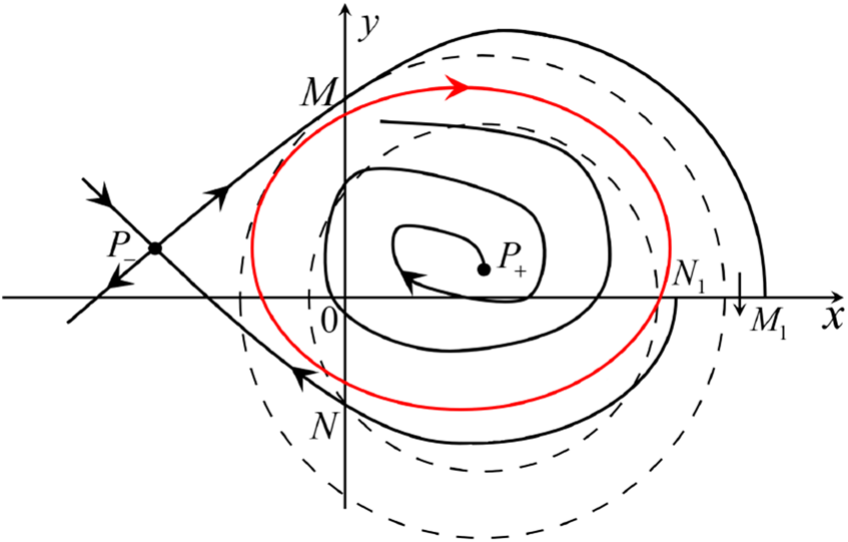
$$x_{M_1}>x_{N_1}$$

**Fig. 12:**
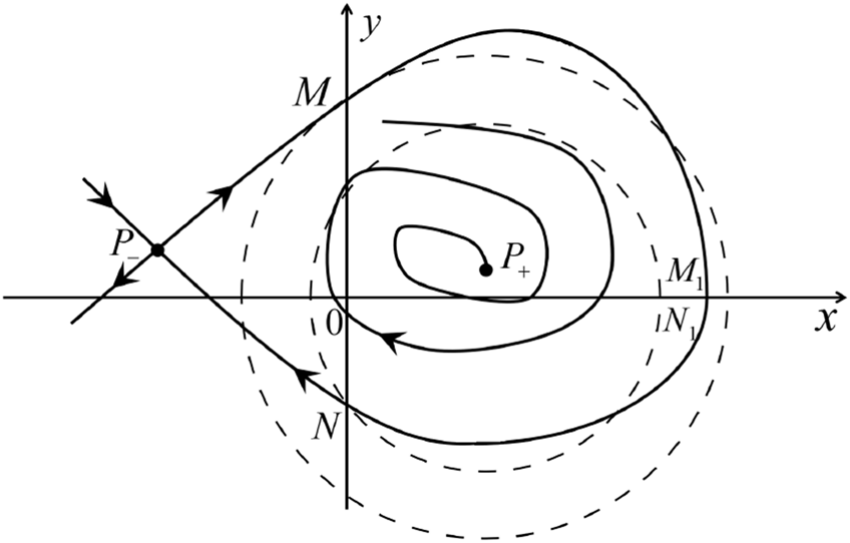
$$x_{M_1}=x_{N_1}$$

**Fig. 13:**
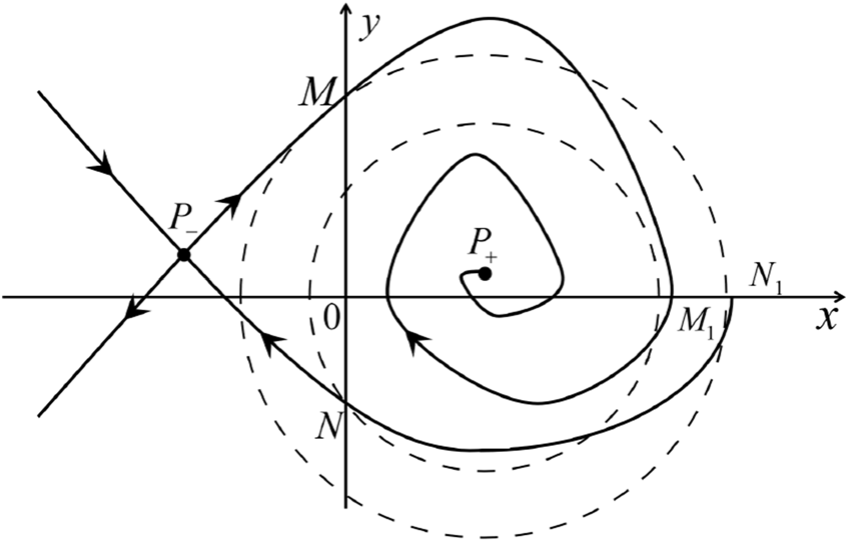
$$x_{M_1}<x_{N_1}$$

Then there is only one infinite equilibrium point of systems $S_+$ in the local chart $U_1$, namely $p= (1,0),$ and the origin *O* of the local chart $U_2$ is not an infinite equilibrium point. The eigenvalues of the equilibrium point *p* are 0 and 1. Therefore by [5, Theorem 2.19] the infinite equilibrium point *p* is a semi-hyperbolic saddle-node. By the direction of vector fields in Fig.  9 we have the stable manifold of $P_-$ intersecting with the *y*-axis comes from the semi-hyperbolic saddle-node *p*. While the unstable manifold of $P_-$ intersecting with the *y*-axis goes to the stale node $P_+$. They are shown in Figs. 11, 12 and 13. By the continuity of systems (I) in the interval $-2<\sigma <0$ we prove the claim.

**Fig. 14:**
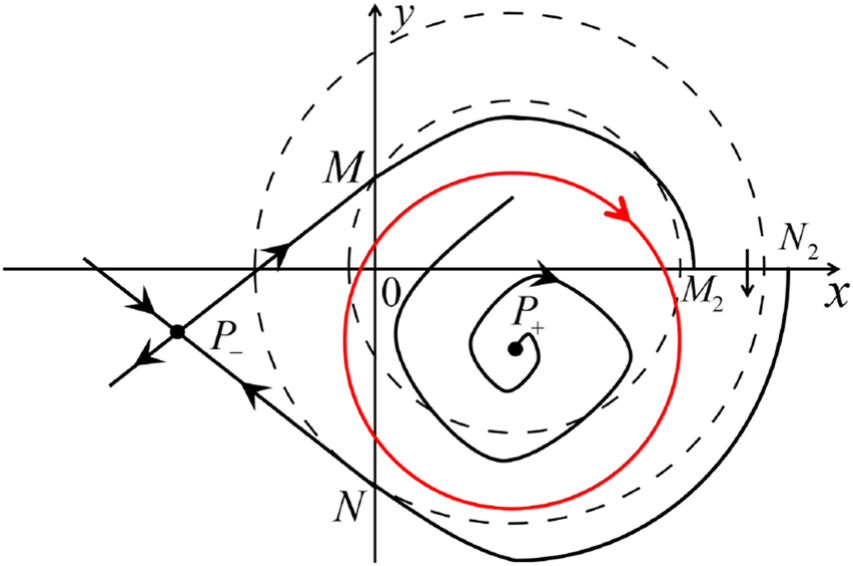
$$x_{M_2}<x_{N_2}$$

**Fig. 15:**
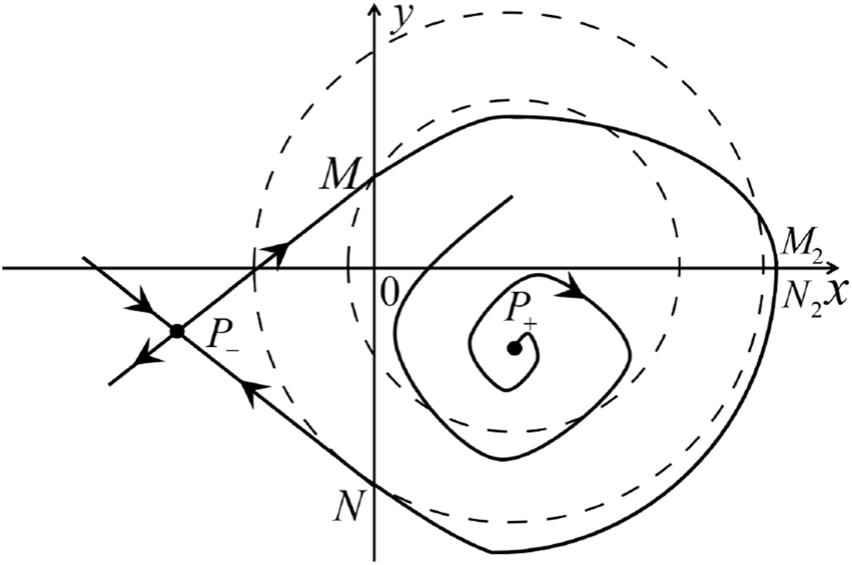
$$x_{M_2}=x_{N_2}$$

**Fig. 16:**
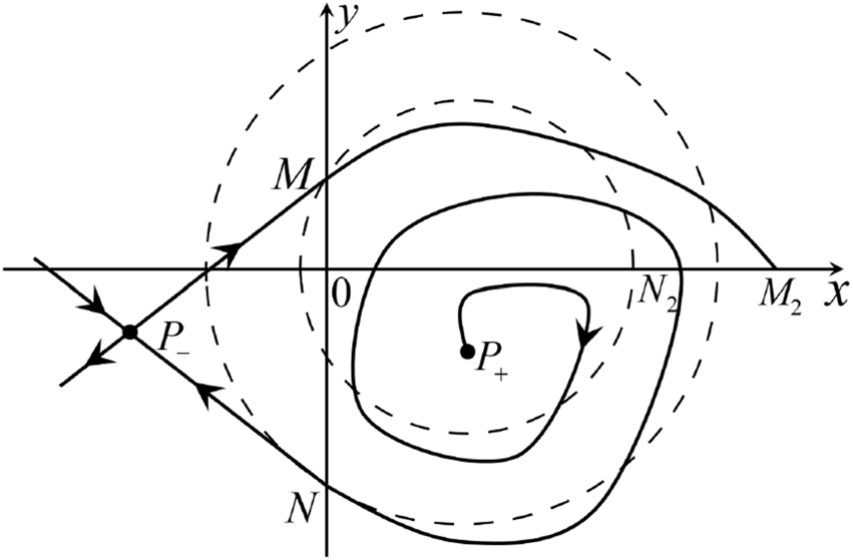
$$x_{M_2}>x_{N_2}$$

We consider the case that $\varsigma <0$ and $0<\sigma <2$ by the analogue method. Let $M_2(x_{M2},0)$ (respectively $N_2(x_{N2},0)$) be the intersection point of the *x*-axis with the orbit starting from *M* (respectively *N*) if they intersect. When $\sigma =0$ we see $x_{M2}<x_{N2}$ since $|\left( \varsigma -\sqrt{\varsigma ^2+4}\right) /2)|>\left( \varsigma +\sqrt{\varsigma ^2+4}\right) /2)$ as $\varsigma <0$. For $\sigma >0$ small enough we see that the inequality $x_{M2}<x_{N2}$ still holds. Similarly by the Poincaré–Bendixson Theorem there exist periodic orbits in the region surrounded by $P_-M\cup MM_2\cup M_2N_2 \cup N_2N \cup NP_-$. Furthermore we claim that there exists a value $\sigma _*$ such that $x_{N2}=x_{M2}$. This means that there exists a homoclinic orbit at $P_-$ and surrounding $P_+$. In fact when $\sigma =2$ there is only one infinite equilibrium point of systems $S_+$ in the local chart $U_1$, namely $q= (-1,0),$ and the origin *O* of the local chart $U_2$ is not an infinite equilibrium point. The eigenvalues of the equilibrium point *q* are 0 and $-1$, and therefore the infinite equilibrium point *q* is a semi-hyperbolic saddle-node. By the directions of the vector field in Fig. 10 we have that the stable manifold of $P_-$ intersecting the *y*-axis comes from the semi-hyperbolic saddle-node *q*. While the unstable manifold of $P_-$ intersects the *y*-axis and goes to the stable node $P_+$. They are shown in Figs. 14, 15 and 16. This competes the proof of systems (I).

For systems (II) we see that the differential systems $S_+$ (respectively $S_-$) have the equilibrium point $P_+=(0,0)$ (respectively $P_-=(0,0)$). Namely $P_-=P_+=:P$. Then the equilibrium point *P* is real for both systems $S_+$ and $S_-$. The eigenvalues of the equilibrium point $P_-$ are $\mu _-=({\hat{b}}-\sqrt{{\hat{b}}^{2}+4})/2$ and $\mu _+=({\hat{b}}+\sqrt{{\hat{b}}^{2}+4})/2$. Clearly, $\mu _-<0<\mu _+$, implying that $P_-$ is a saddle. Clearly there is no periodic orbits for systems (II).

For systems (III) the differential systems $S_+$ have the equilibrium point $P_+=(-1,-a)$, while the differential systems $S_-$ have the equilibrium point $P_-=(1,b)$. Then the equilibrium point $P_+$ (respectively $P_-$) is virtual for the differential systems $S_+$ (respectively $S_-$). Namely systems (III) have no finite equilibrium points, and therefore there is also no periodic orbits for systems (III).

For systems (IV) note that $\varsigma =\beta /|a|\ne 0$, otherwise $a\beta -\alpha b=0$ because $b=0$ in the case. Then the differential systems $S_+$ have the equilibrium point $P_+=(1,-\sigma /\varsigma ).$ While the differential systems $S_-$ have the equilibrium point $P_-=(-1,-\sigma /\varsigma ).$ Moreover the equilibrium point $P_+$ (respectively $P_-$) is real for the differential systems $S_+$ (respectively $S_-$). The eigenvalues of the equilibrium point $P_+$ are 1 and $\varsigma$. So if $\varsigma >0$ (respectively $\varsigma <0$) then $P_+$ is an unstable node (respectively a saddle). The eigenvalues of the equilibrium point $P_-$ are $-1$ and $\varsigma$. Then $P_-$ is a saddle if $\varsigma >0$ and a stable node if $\varsigma <0$. Clearly there is also no periodic orbits for systems (IV).

For systems (V) we check that systems $S_-$ (respectively $S_-$) of systems (V) have an equilibrium point $P_+=(-1,\sigma /\varsigma )$ (respectively $P_-=(1,\sigma /\varsigma )$). Moreover they are both virtual, and therefore there is no periodic orbits for systems (V).

For systems (VI) we have that the differential systems $S_+$ (respectively $S_-$) have the equilibrium point $P_+=(0,0)$ (respectively $P_-=(0,0)$). Namely $P_-=P_+=:P$. Then the equilibrium point *P* is real for both systems $S_+$ and $S_-$. The eigenvalues of the equilibrium point $P_+$ are 1 and $\varsigma$. So if $\varsigma >0$ (respectively $\varsigma <0$) then $P_+$ is an unstable node (respectively a saddle). The eigenvalues of the equilibrium point $P_-$ are $-1$ and $\varsigma$. Then $P_-$ is a saddle if $\varsigma >0$ and a stable node if $\varsigma <0$. Thus there is no periodic orbits for systems (VI) and therefore the proof is completed. $\square$

### Phase Portraits in the Poincaré Disc of Systems (I)

#### The Finite and Infinite Equilibrium Points

**Table 2:** The local phase portraits at the finite and infinite equilibrium points of the continuous piecewise differential systems (I)

Systems	Conditions	Finite equilibrium points	Infinite equilibrium points
(I)	(I-1): $\sigma <-2$	$P_+$(stable node)	$p_+$(saddle)
			$p_-$(unstable node)
		$P_-$(saddle)	$q_+$(stable node)
			$q_-$(unstable node)
	(I-2): $\sigma =-2$	$P_+$(stable node)	*p*(semi-hyperbolic saddle-node)
		$P_-$(saddle)	$q_+$(stable node)
			$q_-$(unstable node)
	(I-3): $-2<\sigma <0$	$P_+$(stable focus)	
		$P_-$(saddle)	$q_+$(stable node)
			$q_-$(unstable node)
	(I-4): $\sigma =0$	$P_+$(center)	
		$P_-$(saddle)	$q_+$(stable node)
			$q_-$(unstable node)
	(I-5): $0<\sigma <2$	$P_+$(unstable focus)	
		$P_-$(saddle)	$q_+$(stable node)
			$q_-$(unstable node)
	(I-6): $\sigma =2$	$P_+$(unstable node)	*p*(semi-hyperbolic saddle-node)
		$P_-$(saddle)	$q_+$(stable node)
			$q_-$(unstable node)
	(I-7): $\sigma >2$	$P_+$(unstable node)	$p_+$(stable node)
			$$p_-$$(saddle)
		$P_-$(saddle)	$q_+$(stable node)
			$q_-$(unstable node)

From the proof of Lemma 3 we obtain the results of the finite equilibrium points for systems (I).

For the infinite equilibrium points we write the differential systems $S_+$ in the local charts $U_1$ and $U_2$. Then in the local chart $U_1$ systems $S_+$ write

17 $$\begin{aligned} {{\dot{u}}}= -1-\sigma u+v-{u}^{2}, \quad {{\dot{v}}}= -\sigma v-uv; \end{aligned}$$

and in the local chart $U_2$ become

$$\begin{aligned} {{\dot{u}}}=1+\sigma u+{u}^{2}-uv, \quad {{\dot{v}}}= uv-{v}^{2}. \end{aligned}$$

We separate the study of the infinite equilibrium points of systems $S_+$ in three cases.

*Case* (I$1_+$): $\sigma >2$ or $\sigma <-2$. Then there are only two infinite equilibrium points of systems $S_+$ in the local chart $U_1$, namely $p_\pm = \big ((-\sigma \pm \sqrt{\sigma ^2-4})/2,0\big )$ and the origin of the local chart $U_2$ is not an infinite equilibrium point.

The eigenvalues of the equilibrium point $p_+$ are $-\sqrt{\sigma ^{2}-4}$ and ${\lambda }_p=-(\sigma +\sqrt{\sigma ^{2}-4})/2$. If $\sigma >2$ then ${\lambda }_p<0$, implying that $p_+$ is a stable node, and if $\sigma <-2$ then ${\lambda }_p>0$, implying that $p_+$ is a saddle.

The eigenvalues of the equilibrium point $p_-$ are $\sqrt{\sigma ^{2}-4}$ and $\mu _p=-(\sigma -\sqrt{\sigma ^{2}-4})/2$. If $\sigma >2$ then $\mu _p<0$, implying that $p_-$ is a saddle, and if $\sigma <-2$ then $\mu _p>0$, implying that $p_-$ is an unstable node.

*Case* (I$2_+$): $\sigma =-2$ and $\sigma =2$. Then there is only one infinite equilibrium point of systems $S_+$ in the local chart $U_1$, namely $p= (-\sigma /2,0),$ and the origin *O* of the local chart $U_2$ is not an infinite equilibrium point. The eigenvalues of the equilibrium point *p* are 0 and $-\sigma /2\ne 0$. Therefore by [5, Theorem 2.19] the infinite equilibrium point *p* is a semi-hyperbolic saddle-node.

*Case* (I$3_+$): $-2<\sigma <2$. Then systems $S_+$ have no infinite equilibrium points in the local chart $U_1$ and at the origin of the local chart $U_2$.

Again we write the differential systems $S_-$ in the local charts $V_1$ and $U_2$. Then in the local chart $V_1$ systems $S_-$ write

18 $$\begin{aligned} {{\dot{u}}}=1-\varsigma u+v-{u}^{2}, \quad {{\dot{v}}}= -\varsigma v-uv; \end{aligned}$$

and in the local chart $U_2$ become

$$\begin{aligned} {{\dot{u}}}=1+\varsigma u-{u}^{2}-uv, \quad {{\dot{v}}}= -uv-{v}^{2}. \end{aligned}$$

As we did for the systems $S_+$ there are only two infinite equilibrium points of systems $S_-$ in the local chart $V_1$, namely $q_\pm = \big ((-\varsigma \pm \sqrt{\varsigma ^{2}+4})/2,0\big )$ and the origin of the local chart $U_2$ is not an infinite equilibrium point.

The eigenvalues of the equilibrium point $q_+$ are $-\sqrt{\varsigma ^{2}+4}$ and ${\lambda }_q=-(\varsigma +\sqrt{\varsigma ^{2}+4})/2$. Clearly ${\lambda }_q<0$, implying that $q_+$ is a stable node. The eigenvalues of the equilibrium point $q_-$ are $\sqrt{\varsigma ^{2}+4}$ and $\mu _q=-(\varsigma -\sqrt{\varsigma ^{2}+4})/2$, therefore $q_-$ is an unstable node since $\mu _q>0$.

In summary from the above discussion, we obtain the results of Table 2.

#### The Global Phase Portraits in the Poincaré Disc

We below give a discussion for passing from the local phase portraits from all the finite and infinite equilibrium points to the global phase portraits in the Poincaré disc.

Note by (17) and (18) that the right hand sides of the equation ${{\dot{v}}}$ both have a common factor *v*, implying that the infinity is invariant, i.e, formed by orbits. Besides we observe that ${{\dot{x}}}=y$ and ${{\dot{y}}}=1$ on the *y*-axis. Then starting at points lying on the positive *y*-axis, all orbits go into the half plane $x\ge 0$ while starting at points lying in the negative *y*-axis, all orbits go into the half plane $x\le 0$. According to Table 2 and the direction of the vector fields shown in Figs. 5–10, we below discuss the global phase portraits in the following cases.

**Fig. 17:**
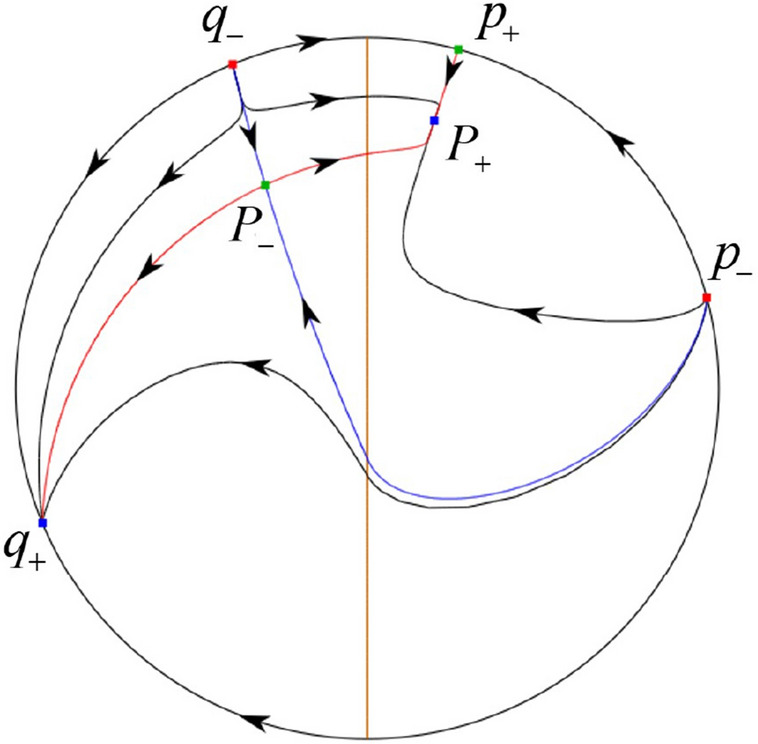
$$S=15,R=4$$

In the case (I-1) one stable separatrix of the saddle $P_-$ comes from the unstable node $p_-$ and the second stable separatrix of $P_-$ comes from the unstable node $q_-$. One unstable separatrix of $P_-$ goes to the stable node $q_+$ and the second unstable separatrix of $P_-$ goes to the stable node $P_+$. An unstable separatrix of the saddle $p_+$ goes to the stable node $P_+$. The remaining orbits of the phase portrait are determined where they start and where they end by the type of stability of the equilibrium points and by the Poincaré–Bendixson theorem (see for instance Theorem 1.25 of [1]). Thus the global phase portrait is given in Fig. 17.

**Fig. 18:**
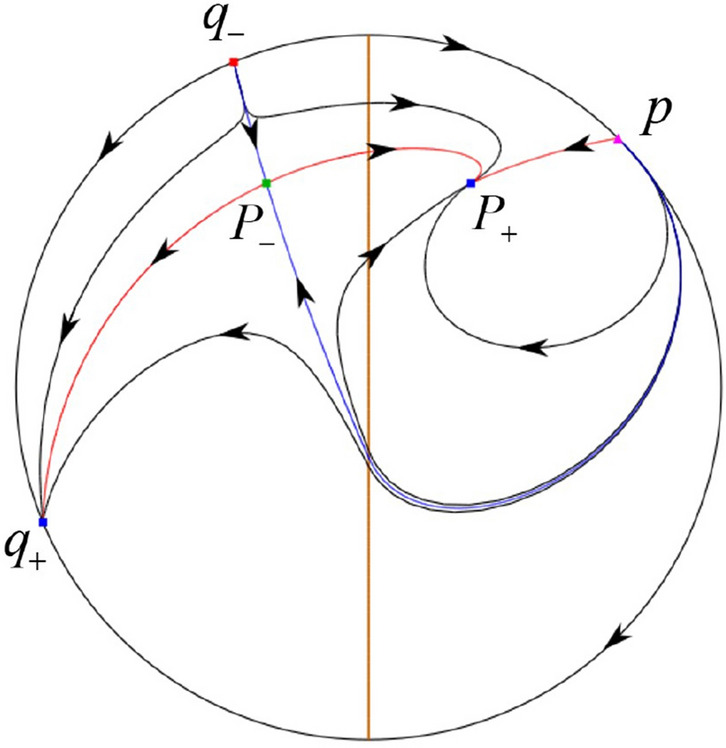
$$S=13, R=4$$

In the case (I-2) one stable separatrix of the saddle $P_-$ comes from the unstable node $q_-$ and the second stable separatrix of $P_-$ comes from the semi-hyperbolic saddle-node *p*. One unstable separatrix of $P_-$ goes to the stable node $q_+$ and the second unstable separatrix of $P_-$ goes to the stable node $P_+$. An unstable separatrix of the saddle-node *p* goes to the stable node $P_+$. The remaining orbits of the phase portrait are determined where they start and where they end by the type of stability of the equilibrium points and by the Poincaré–Bendixson theorem. Thus the global phase portrait is given in Fig. 18.

**Fig. 19:**
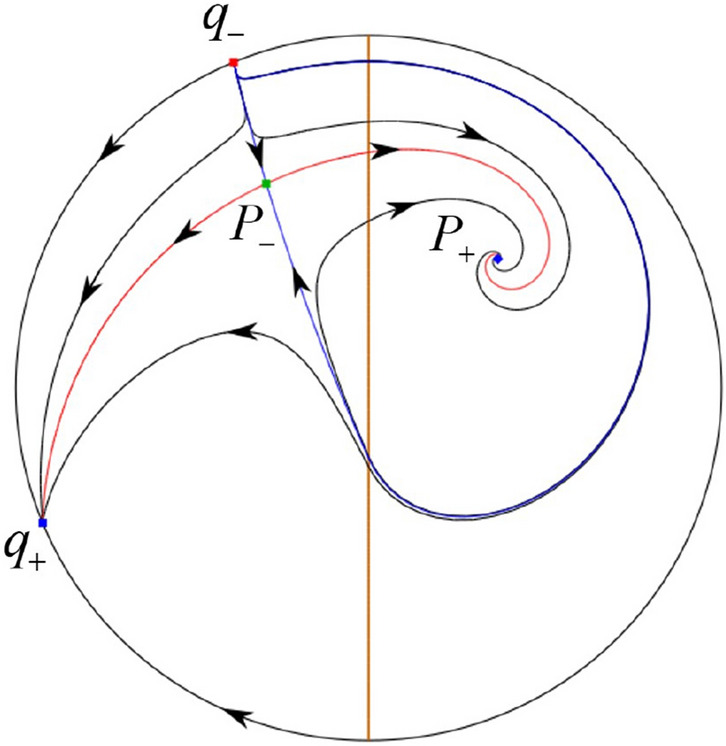
$$S=10, R=3$$

In the case (I-3) two stable separatrices of the saddle $P_-$ come from the unstable node $q_-$. One unstable separatrix of $P_-$ goes to the stable node $q_+$ and the second unstable separatrix of $P_-$ goes to the stable node $P_+$. The remaining orbits of the phase portrait are determined by the type of stability of the equilibrium points and by the Poincaré–Bendixson theorem. Thus the global phase portrait is given in Fig. 19,

In the case (I-4) one unstable separatrix of the saddle $P_-$ goes to the stable node $q_+$. One stable separatrix of $P_-$ comes from the unstable node $q_-$. Then discussions are divided into seven subcases.

**Fig. 20:**
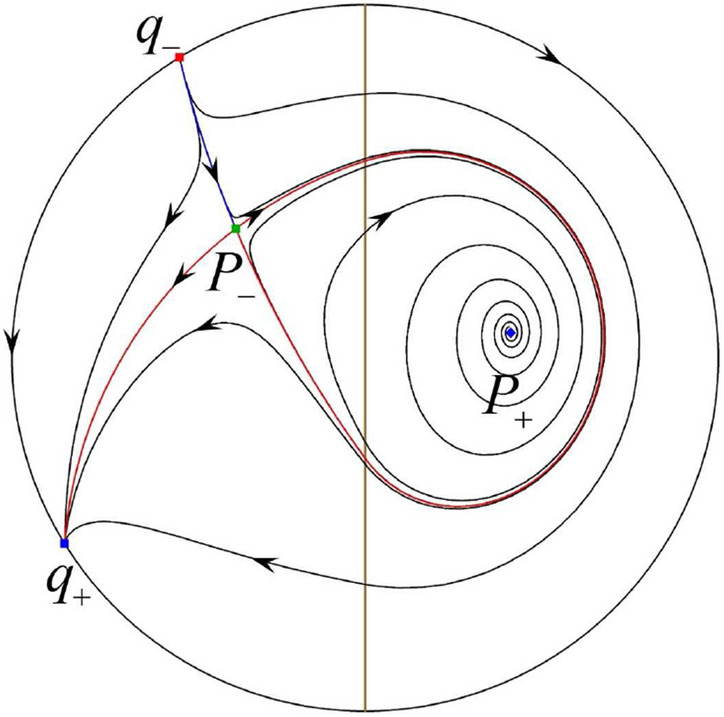
$$S=9, R=3$$

**Fig. 21:**
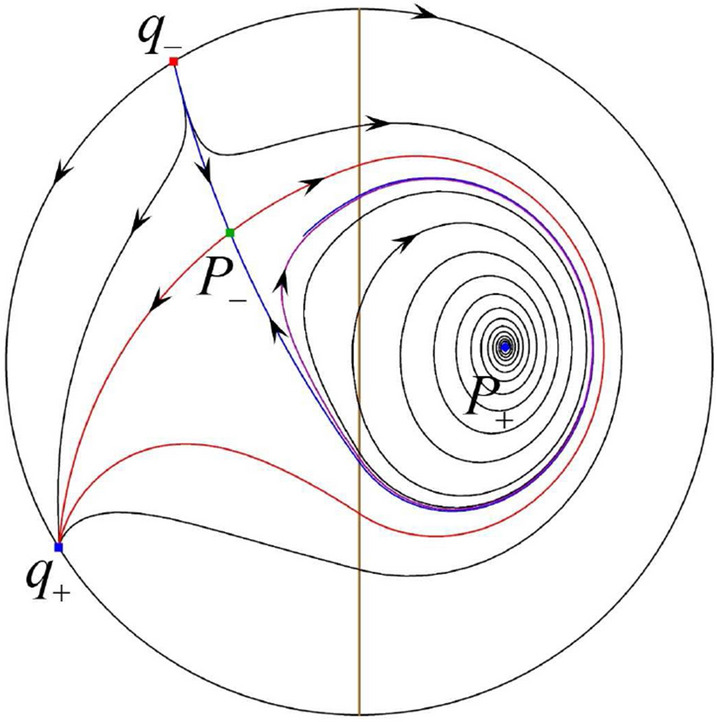
$$S=11, R=4$$

**Fig. 22:**
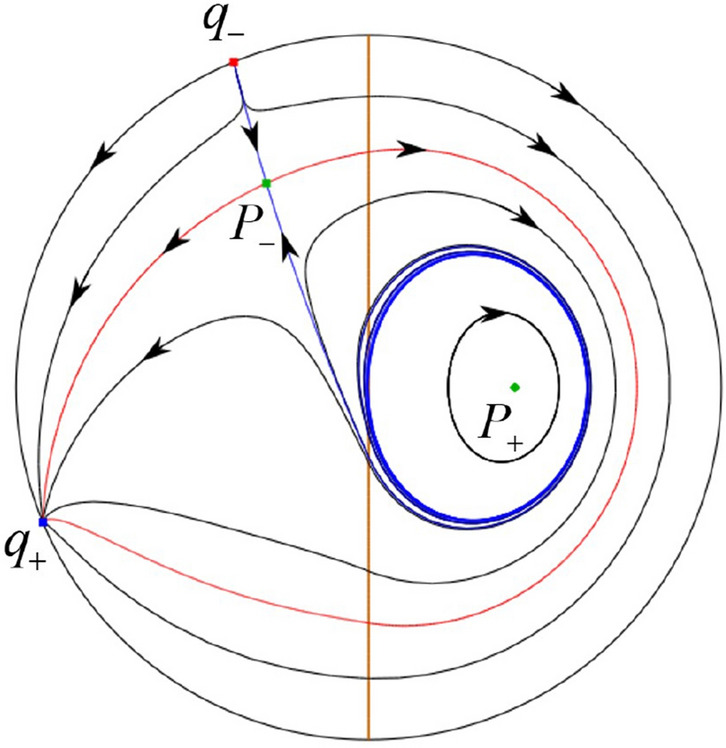
$$S=11, R=4$$

**Fig. 23:**
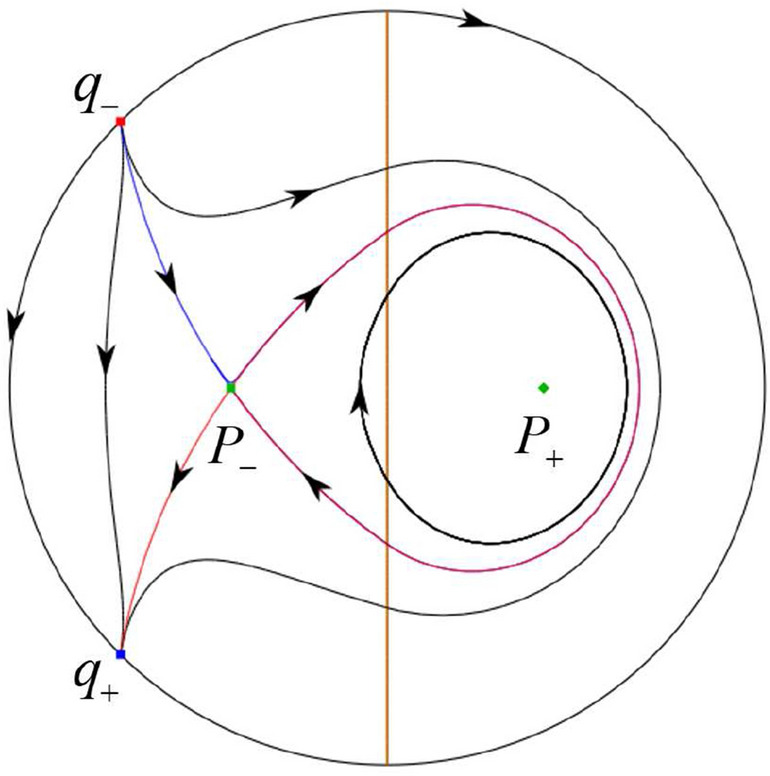
$$S=10, R=3$$

**Fig. 24:**
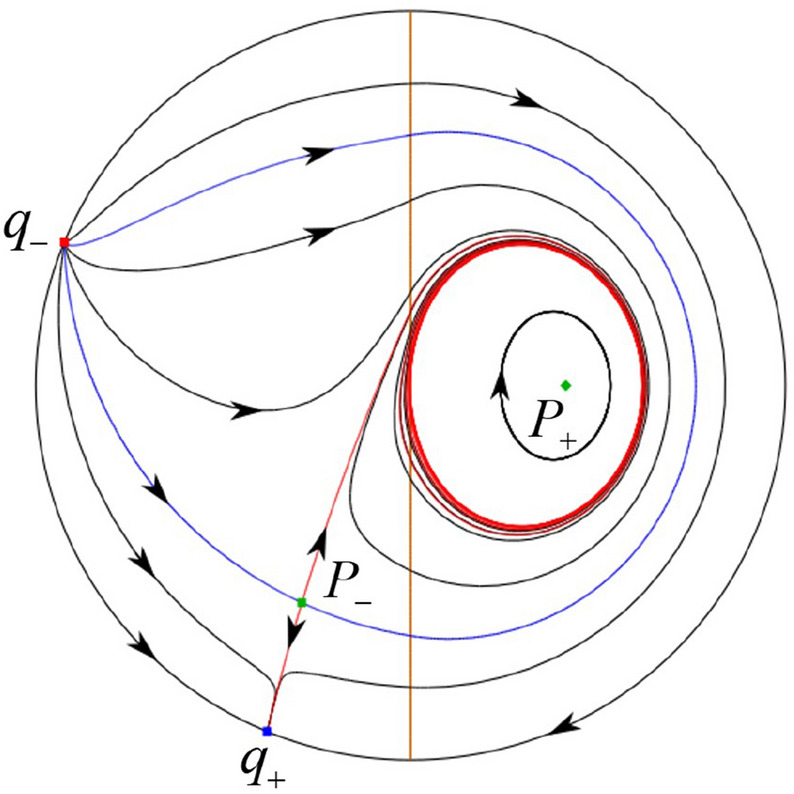
$$S=11, R=4$$

**Fig. 25:**
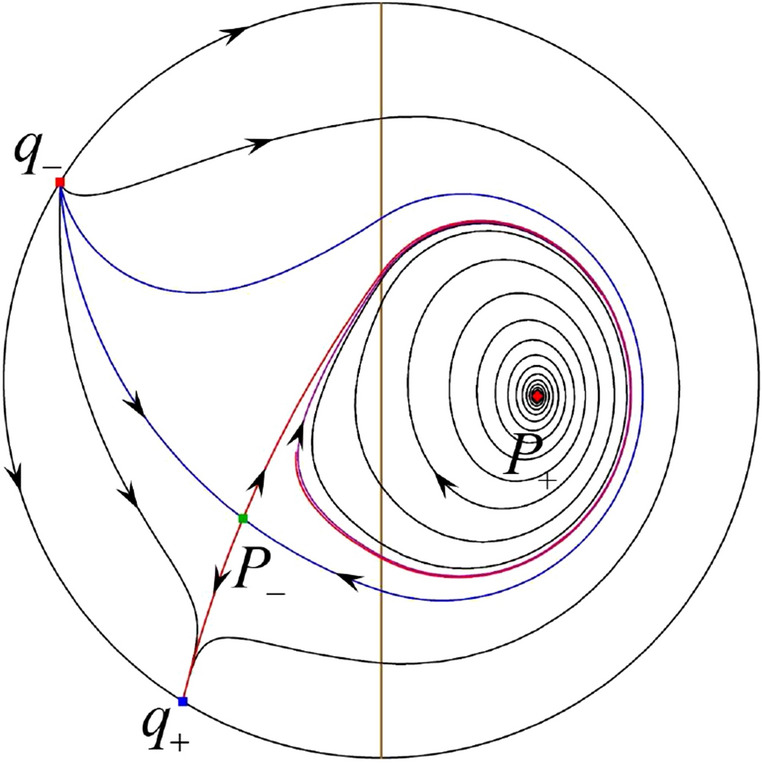
$$S=11, R=4$$

**Fig. 26:**
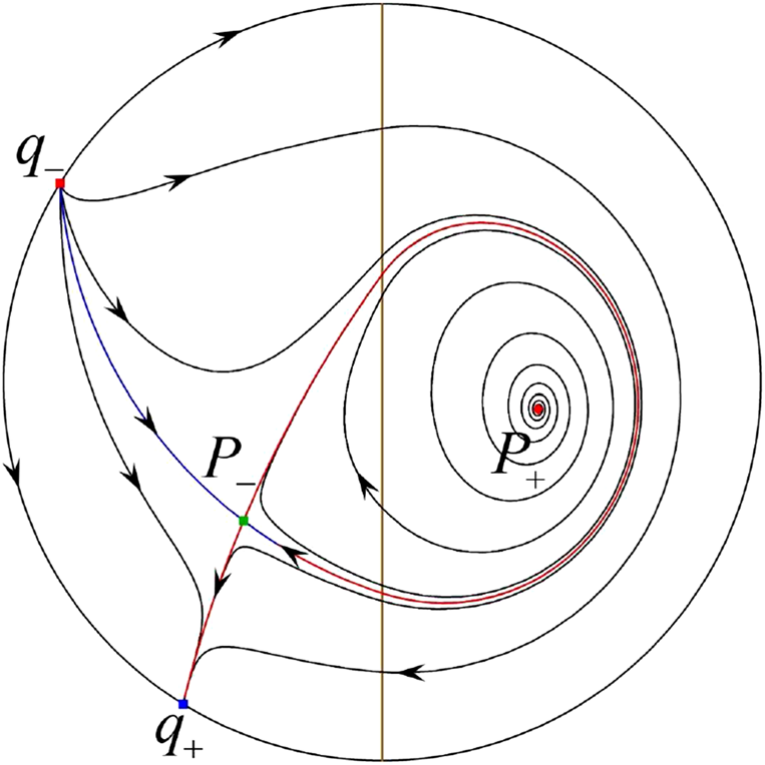
$$S=9, R=3$$

(i) The third separatrix of $P_-$ is a homoclinic orbit at $P_-$.
(ii) The second unstable separatrix of the saddle $P_-$ goes to $q_+$ and the second stable separatrix of $P_-$ comes from a limit cycle in the strip $-1<x<0$. The limit cycle is also a separatrix.
(iii) The second unstable separatrix of $P_-$ goes to $q_+$ and the second stable separatrix of $P_-$ comes from the periodic orbit ${\mathcal {C}}: (x-1)^2+y^2=1$.
(iv) The third separatrix of $P_-$ is a homoclinic orbit at $P_-$. On the other hand the periodic obit close to the homoclinic orbit is also a separatrix.
(v) The second unstable separatrix of $P_-$ goes around a periodic orbit ${\mathcal {C}}: (x-1)^2+y^2=1$ and the second stable separatrix of $P_-$ comes from $q_-$.
(vi) The second unstable separatrix of $P_-$ goes around a limit cycle. The limit cycle is also a separatrix. The second stable separatrix of $P_-$ comes from $q_-$.
(vii) The third separatrix of $P_-$ is a homoclinic orbit at $P_-$. The remaining orbits are determined by the Poincaré–Bendixson theorem and by the type of stability of the equilibrium points. Thus the global phase portraits of the seven subcases are shown in Figs. 20–26, respectively.

**Fig. 27:**
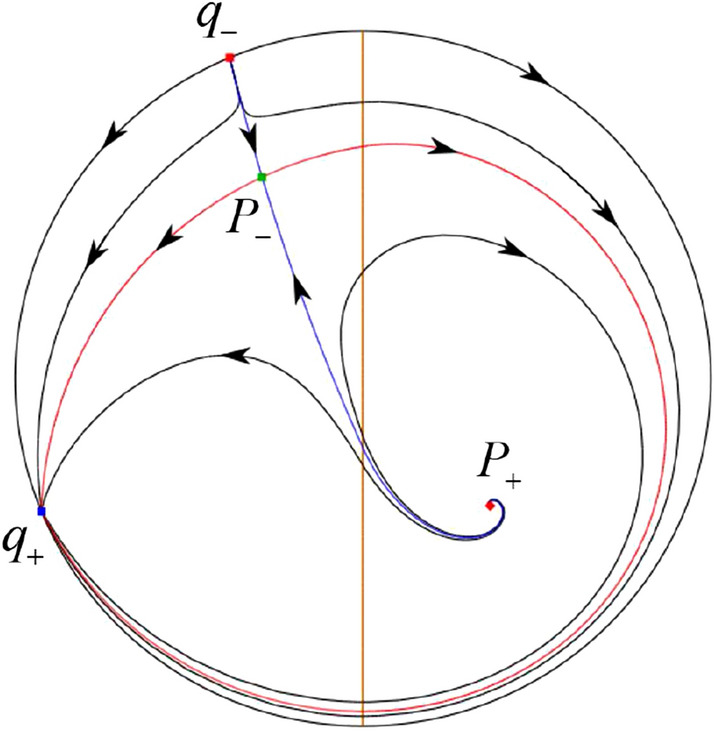
$$S=10,R=3$$

In the case (I-5) one stable separatrix of the saddle $P_-$ comes from the unstable node $q_-$ and the second stable separatrix of $P_-$ comes from the unstable node $P_+$. Two unstable separatrices of $P_-$ go to the stable node $q_+$. By the Poincaré–Bendixson theorem and by the type of stability of the equilibrium points we see where the remaining orbits of the phase portrait start and end. Thus the global phase portrait is given in Fig. 27.

**Fig. 28:**
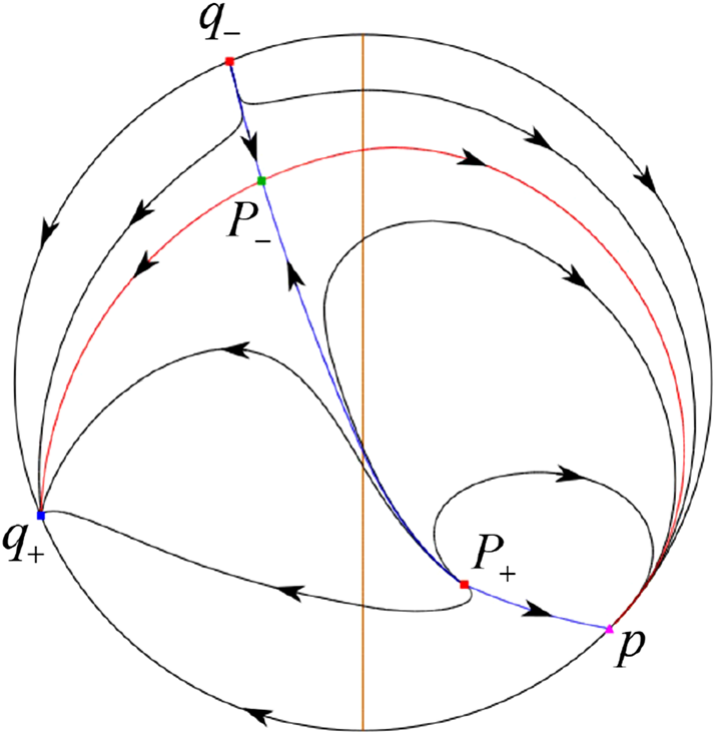
$$S=13,R=4$$

In the case (I-6) one stable separatrix of the saddle $P_-$ comes from the unstable node $q_-$ and the second stable separatrix of $P_-$ comes from the unstable node $P_+$. One unstable separatrix of $P_-$ goes to the stable node $q_+$ and the second unstable separatrix of $P_-$ goes to the semi-hyperbolic saddle-node *p*. A stable separatrix of the saddle-node *p* comes from the unstable node $P_+$. We determine the remaining orbits of the phase portrait by the Poincaré–Bendixson theorem and by the type of stability of the equilibrium points. Thus the global phase portrait is shown in Fig. 28.

**Fig. 29:**
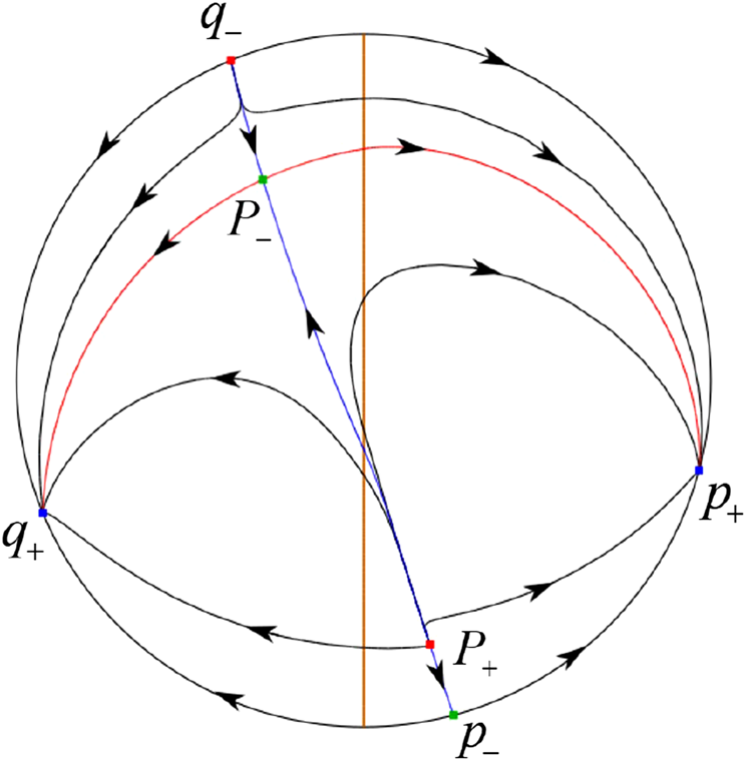
$$S=15,R=4$$

In the case (I-7) one stable separatrix of the saddle $P_-$ comes from the unstable node $q_-$ and the second stable separatrix of $P_-$ comes from the unstable node $P_+$. One unstable separatrix of $P_-$ goes to the stable node $q_+$ and the second unstable separatrix of $P_-$ goes to the stable node $p_+$. An stable separatrix of the saddle $p_-$ comes from $P_+$. By the Poincaré–Bendixson theorem and by the type of stability of the equilibrium points we get the remaining orbits of the phase portrait. Thus the global phase portrait is given in Fig. 29.

### Phase Portraits in the Poincaré Disc of Systems (II)

**Table 3:** The local phase portraits at the finite and infinite equilibrium points of the continuous piecewise differential systems (II)

Systems	Conditions	Finite equilibrium points	Infinite equilibrium points
(II)	(II-1): $\sigma <-2$	*P*(stable node)	$p_+$(unstable node)
			$$p_-$$(saddle)
		*P*(saddle)	$q_+$(unstable node)
			$$q_-$$(stable node)
	(II-2): $\sigma =-2$	*P*(stable node)	*p*(semi-hyperbolic saddle-node)
		*P*(saddle)	$q_+$(unstable node)
			$$q_-$$(stable node)
	(II-3): $-2<\sigma <0$	*P*(stable focus)	
		*P*(saddle)	$q_+$(unstable node)
			$$q_-$$(stable node)
	(II-4): $\sigma =0$	*P*(center)	
		*P*(saddle)	$q_+$(unstable node)
			$$q_-$$(stable node)
	(II-5): $0<\sigma <2$	*P*(unstable focus)	
		*P*(saddle)	$q_+$(unstable node)
			$$q_-$$(stable node)
	(II-6): $\sigma =2$	*P*(unstable node)	*p*(semi-hyperbolic saddle-node)
		*P*(saddle)	$q_+$(unstable node)
			$$q_-$$(stable node)
	(II-7): $\sigma >2$	*P*(unstable node)	$p_+$(saddle)
			$$p_-$$(stable node)
		*P*(saddle)	$q_+$(unstable node)
			$$q_-$$(stable node)

#### The Finite and Infinite Equilibrium Points

As it is given in the proof of Lemma 3 the differential systems $S_+$ and $S_-$ have the same real equilibrium point *P*(0, 0) and *P* is a saddle for systems $S_-$.

For systems $S_+$ the eigenvalues of *P* are ${\lambda }_-=(\sigma -\sqrt{\sigma ^{2}-4})/2$ and ${\lambda }_+=(\sigma +\sqrt{\sigma ^{2}-4})/2$. So if $\sigma \le -2$ (respectively $\sigma \ge 2$) then ${\lambda }_-\le {\lambda }_+<0$ (respectively ${\lambda }_+\ge {\lambda }_->0$), implying that *P* is a stable (respectively an unstable) node. If $-2<\sigma <0$ (respectively $0<\sigma <2$) then ${\lambda }_\pm$ are a pair of imaginary eigenvalues with negative (respectively positive) real part, implying that *P* is a stable (respectively an unstable) focus. If $\sigma =0$ then ${\lambda }_\pm$ are a pair of purely imaginary eigenvalues, implying that *P* is a center.

For the infinite equilibrium points we write the differential systems $S_+$ in the local charts $U_1$ and $U_2$. Then in the local chart $U_1$ systems $S_+$ write

19 $$\begin{aligned} {{\dot{u}}}= 1-\sigma u+u^2, \quad {{\dot{v}}}= -\sigma v+uv; \end{aligned}$$

and in the local chart $U_2$ become

$$\begin{aligned} {{\dot{u}}}=-1+\sigma u-u^2, \quad {{\dot{v}}}= -uv. \end{aligned}$$

We separate the study of the infinite equilibrium points of systems $S_+$ in three cases.

*Case* (II$1_+$): $\sigma >2$ or $\sigma <-2$. Then there are only two infinite equilibrium points of systems $S_+$ in the local chart $U_1$, namely $p_\pm = \big ((\sigma \pm \sqrt{\sigma ^2-4})/2,0\big )$ and the origin of the local chart $U_2$ is not an infinite equilibrium point.

The eigenvalues of the equilibrium point $p_+$ are $\sqrt{\sigma ^{2}-4}$ and ${\lambda }_p=-(\sigma -\sqrt{\sigma ^{2}-4})/2$. If $\sigma >2$ then ${\lambda }_p<0$, implying that $p_+$ is a saddle, and if $\sigma <-2$ then ${\lambda }_p>0$, implying that $p_+$ is an unstable node.

The eigenvalues of the equilibrium point $p_-$ are $-\sqrt{\sigma ^{2}-4}$ and $\mu _p=-(\sigma +\sqrt{\sigma ^{2}-4})/2$. If $\sigma >2$ then $\mu _p<0$, implying that $p_-$ is a stable node, and if $\sigma <-2$ then $\mu _p>0$, implying that $p_-$ is a saddle.

*Case* (II$2_+$): $\sigma =-2$ and $\sigma =2$. Then there is only one infinite equilibrium point of systems $S_+$ in the local chart $U_1$, namely $p= \big (\sigma /2,0\big ),$ and the origin *O* of the local chart $U_2$ is not an infinite equilibrium point. The eigenvalues of the equilibrium point *p* are 0 and $-\sigma /2\ne 0$. Therefore by [5, Theorem 2.19] the infinite equilibrium point *p* is a semi-hyperbolic saddle-node.

*Case* (II$3_+$): $-2<\sigma <2$. Then systems $S_+$ have no infinite equilibrium points in the local chart $U_1$ and at the origin of the local chart $U_2$.

Again we write the differential systems $S_-$ in the local charts $V_1$ and $U_2$. Then in the local chart $V_1$ systems $S_-$ write

20 $$\begin{aligned} {{\dot{u}}}=-1-{\hat{b}}u+u^2, \quad {{\dot{v}}}= -{\hat{b}}v+uv; \end{aligned}$$

and in the local chart $U_2$ become

$$\begin{aligned} {{\dot{u}}}=-1+{\hat{b}}u+u^2, \quad {{\dot{v}}}= uv. \end{aligned}$$

As we did for the systems $S_+$ there are only two infinite equilibrium points of systems $S_-$ in the local chart $V_1$, namely $q_\pm = \big (({\hat{b}}\pm \sqrt{{\hat{b}}^{2}+4})/2,0\big )$ and the origin of the local chart $U_2$ is not an infinite equilibrium point.

The eigenvalues of the equilibrium point $q_+$ are $\sqrt{{\hat{b}}^{2}+4}$ and ${\lambda }_q=-({\hat{b}}-\sqrt{{\hat{b}}^{2}+4})/2$. Clearly, ${\lambda }_q>0$, implying that $q_+$ is an unstable node. The eigenvalues of the equilibrium point $q_-$ are $-\sqrt{\varsigma ^{2}+4}$ and $\mu _q=-(\varsigma +\sqrt{\varsigma ^{2}+4})/2$, therefore $q_-$ is a stable node since $\mu _q<0$.

In summary from the above discussion, we obtain the results of Table 3.

#### The Global Phase Portraits in the Poincaré Disc

Similar to systems (I), by (19) and (20) we see that the right hand sides of the equation ${{\dot{v}}}$ both have a common factor *v*, implying that the infinity is formed by orbits. Further check that ${{\dot{x}}}=-y$ and ${{\dot{y}}}=0$ on the *y*-axis. Then starting at points lying on the positive *y*-axis, all orbits go into the half plane $x\le 0$ while starting at points lying in the negative *y*-axis, all orbits go into the half plane $x\ge 0$. On the other hand for systems $S_-$ there are the horizontal isocline ${\mathcal {H}}: x=0$ and the vertical isocline ${\mathcal {V}}: y={\hat{b}}x$. Also for systems (19) there are two invariant lines $u=(\sigma \pm \sqrt{\sigma ^2-4})/2$, and for systems (20) there are two invariant lines $u=({\hat{b}}\pm \sqrt{{\hat{b}}^{2}+4})/2$. According to Table 3 we below discuss the global phase portraits in the following cases.

**Fig. 30:**
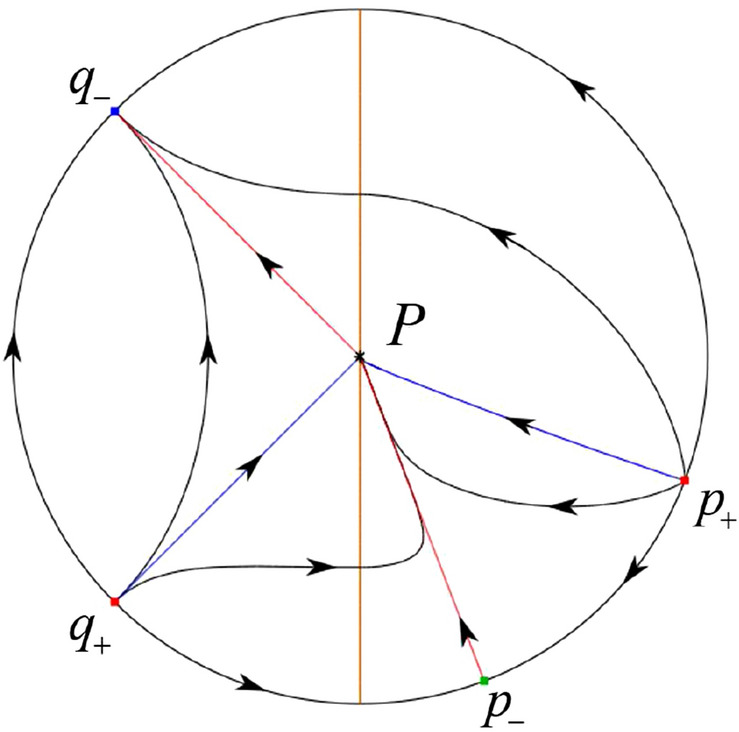
$$S=13,R=4$$

In the case (II-1) two separatrices respectively lying on the line $y=((\sigma +\sqrt{\sigma ^2-4})/2)x$ and $y=(({\hat{b}}-\sqrt{{\hat{b}}^{2}+4})/2)x$ are the orbits at the boundary of a hyperbolic sector at *P*. One stable separatrix of the saddle *P* in the half plane $x\le 0$ comes from the unstable node $q_+$ lying on the line $y=(({\hat{b}}+\sqrt{{\hat{b}}^{2}+4})/2)x$. One unstable separatrix of the saddle $p_-$ lying on the line $y=(({\hat{b}}-\sqrt{{\hat{b}}^{2}+4})/2)x$ goes to the stable node *P* in the half plane $x\le 0$. The remaining orbits of the phase portrait are determined where they start and where they end by the type of stability of the equilibrium points and by the Poincaré–Bendixson theorem. Thus the global phase portrait is shown in Fig. 30.

Note that for the remain cases (II-2)–(II-7) the phase portrait is the same as the case (II-1) in the half plane $x\le 0$. In the half plane $x\ge 0$ the phase portrait is studied in what follows.

**Fig. 31:**
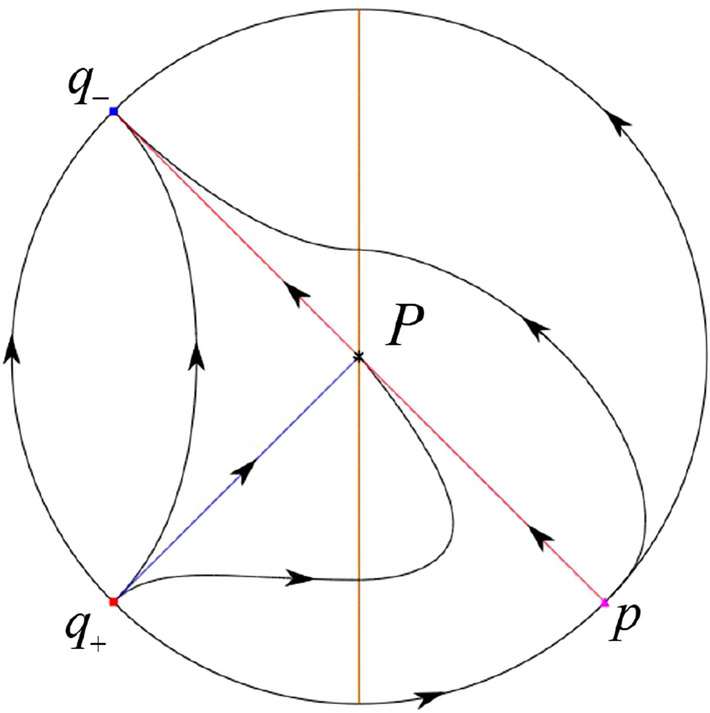
$$S=10,R=3$$

In the case (II-2) one unstable separatrix of the saddle-node *p* goes to the stable node *P* in the half plane $x\ge 0$, which lies on the line $y=(\sigma /2)x$. The remaining orbits of the phase portrait are determined by the type of stability of the equilibrium points and by the Poincaré–Bendixson theorem. Thus the global phase portrait is shown in Fig. 31.

**Fig. 32:**
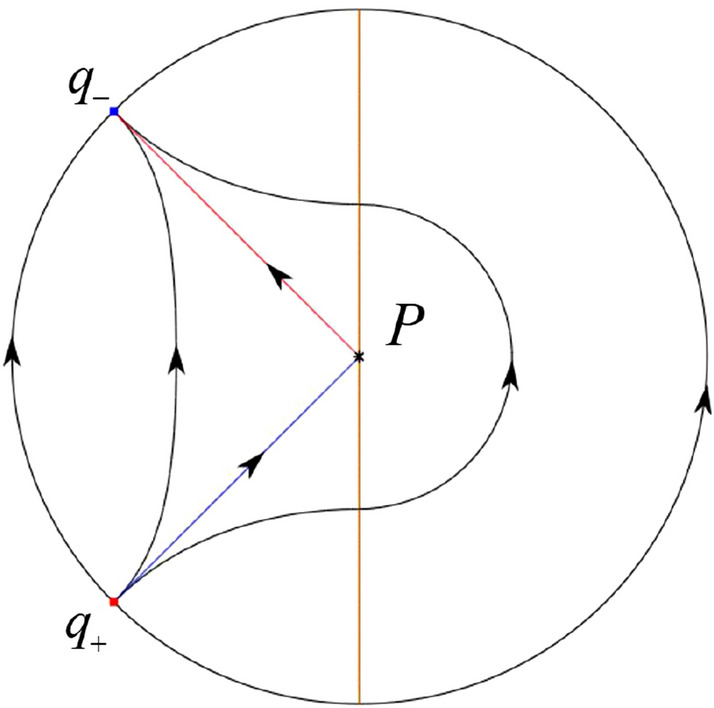
$$S=7,R=2$$

In cases (II-3)–(II-5) there is no separatrices in the half plane $x\ge 0$. The remaining orbits of the phase portrait are determined by the type of stability of the equilibrium points and by the Poincaré–Bendixson theorem. Thus the global phase portraits of these three cases are given in Fig. 32.

**Fig. 33:**
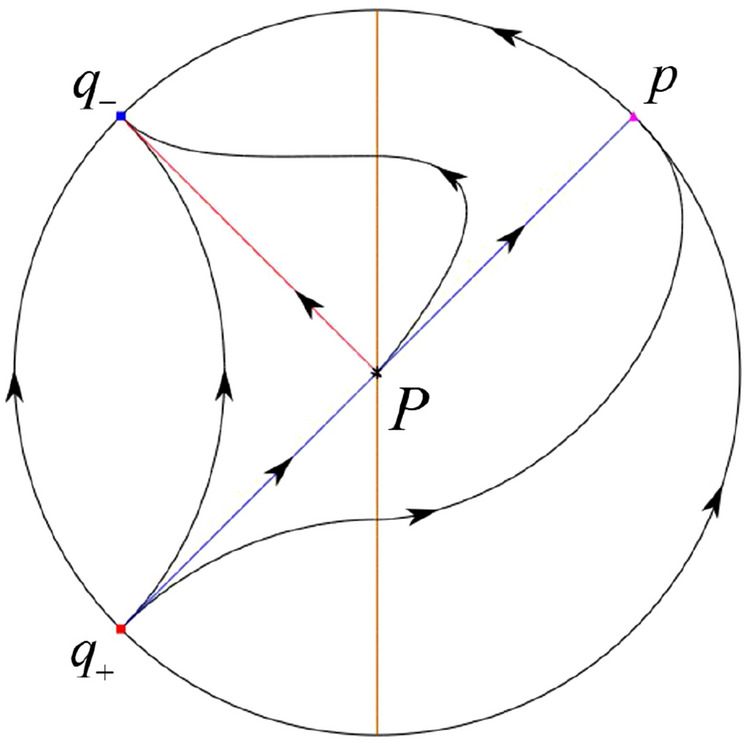
$$S=10,R=3$$

In the case (II-6) one stable separatrix of the saddle-node *p* comes from the unstable node *P* in the half plane $x\ge 0$ lying on the line $y=(\sigma /2) x$. We get the remaining orbits of the phase portrait by the type of stability of the equilibrium points and by the Poincaré–Bendixson theorem. Thus the global phase portrait is shown in Fig. 33.

**Fig. 34:**
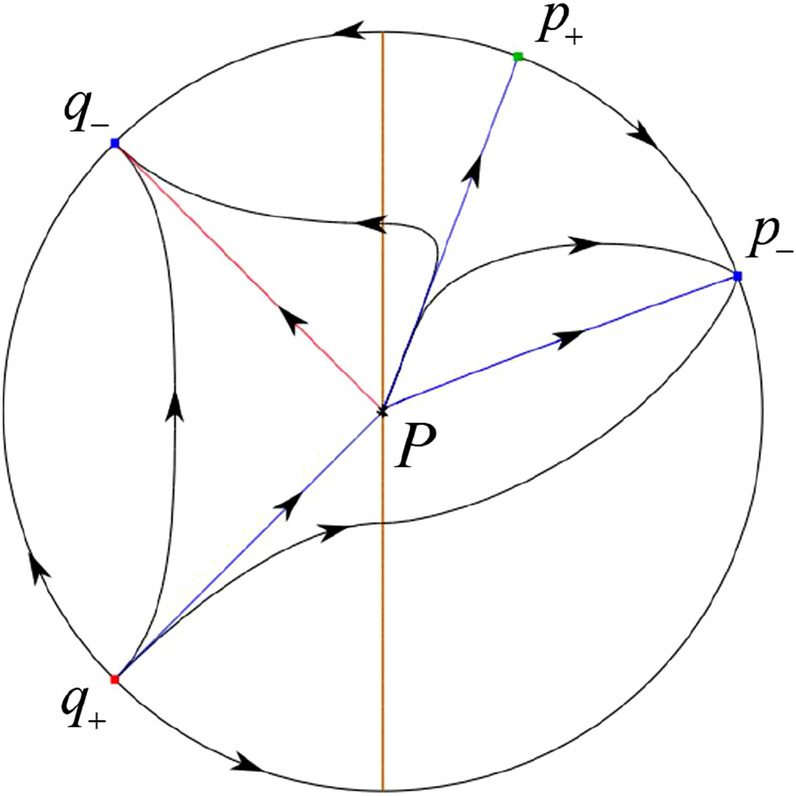
$$S=13,R=4$$

In the case (II-7) a separatrix is one of the orbits at the boundary of a hyperbolic sector at *P* lying on the line $y=((\sigma -\sqrt{\sigma ^2-4})/2)x$ in the half plane $x\ge 0$. One stable separatrix of the saddle $p_+$ comes from the unstable node *P* in the half plane $x\ge 0$ lying on the line $y=((\sigma +\sqrt{\sigma ^2-4})/2)x$. We get the remaining orbits of the phase portrait by the type of stability of the equilibrium points and by the Poincaré–Bendixson theorem. Thus the global phase portrait is shown in Fig. 34.

### Phase Portraits in the Poincaré Disc of Systems (III)

#### The Finite and Infinite Equilibrium Points

As it is given in the proof of Lemma 3 the differential systems $S_+$ have the virtual equilibrium point $P_+=(-1,-a)$, while the differential systems $S_-$ have the virtual equilibrium point $P_-=(1,b)$.

Doing the change $(x,y,t)\rightarrow (-x,y,t)$, systems (III) become

$$\begin{aligned} S_+: \quad {{\dot{x}}}= \varsigma x+ y, \qquad {{\dot{y}}}=x+1, \qquad \text{ if } x\ge 0\text{, } \text{ and }\\ S_-:\quad {{\dot{x}}}= \sigma x+ y, \qquad {{\dot{y}}}=-x+1, \qquad \text{ if } x\le 0. \end{aligned}$$

Systems $S_+$ are the same that systems $S_-$ of systems (I), while systems $S_-$ are the same that systems $S_+$ of systems (I). So from the results of systems (I) for the finite and infinite equilibrium points of systems (III) we get Table 4.

**Table 4:** The local phase portraits at the finite and infinite equilibrium points of the continuous piecewise differential systems (III)

Systems	Conditions	Finite equilibrium points	Infinite equilibrium points
(III)	(III-1): $\sigma <-2$	$P_+$(stable node)	$p_+$(unstable node)
			$$p_-$$(saddle)
		$P_-$(saddle)	$q_+$(unstable node)
			$$q_-$$(stable node)
	(III-2): $\sigma =-2$	$P_+$(stable node)	*p*(semi-hyperbolic saddle-node)
		$P_-$(saddle)	$q_+$(unstable node)
			$$q_-$$(stable node)
	(III-3): $-2<\sigma <0$	$P_+$(stable focus)	
		$P_-$(saddle)	$q_+$(unstable node)
			$$q_-$$(stable node)
	(III-4): $\sigma =0$	$P_+$(center)	
		$P_-$(saddle)	$q_+$(unstable node)
			$$q_-$$(stable node)
	(III-5): $0<\sigma <2$	$P_+$(unstable focus)	
		$P_-$(saddle)	$q_+$(unstable node)
			$$q_-$$(stable node)
	(III-6): $\sigma =2$	$P_+$(unstable node)	*p*(semi-hyperbolic saddle-node)
		$P_-$(saddle)	$q_+$(unstable node)
			$$q_-$$(stable node)
	(III-7): $\sigma >2$	$P_+$(unstable node)	$p_+$(saddle)
			$$p_-$$(stable node)
		$P_-$(saddle)	$q_+$(unstable node)
			$$q_-$$(stable node)

#### The Global Phase Portraits in the Poincaré Disc

Similar to systems (I) we check ${{\dot{x}}}=-y$ and ${{\dot{y}}}=1$ on the *y*-axis. Then starting at points lying on the positive *y*-axis all orbits go into the half plane $x\le 0$, while starting at points lying in the negative *y*-axis all orbits go into the half plane $x\ge 0$. On the other hand the infinity is formed by orbits. According to Table 4 we below discuss the global phase portraits in the following cases.

**Fig. 35:**
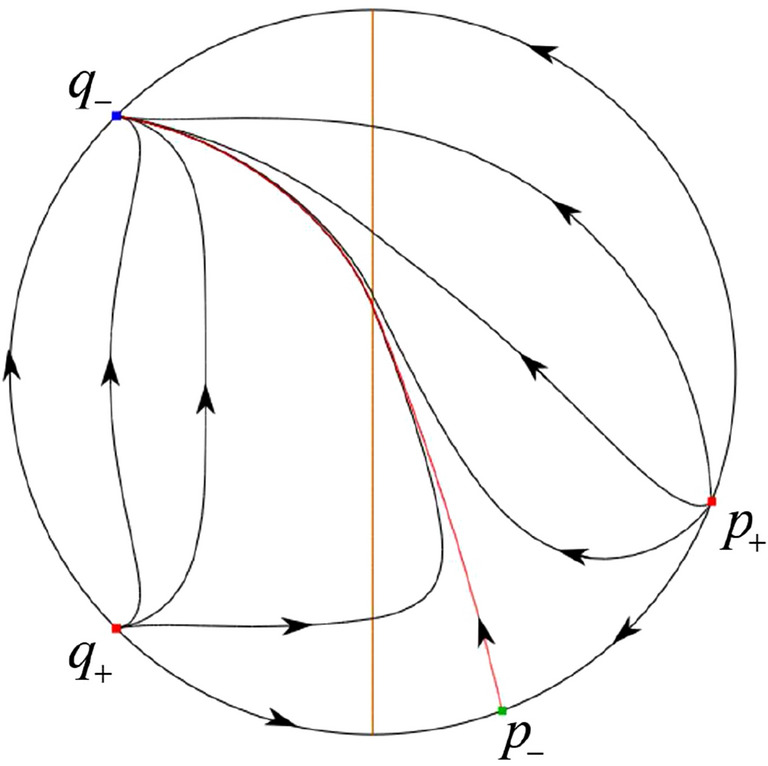
$$S=9,R=2$$

In the case (III-1) an unstable separatrix of the saddle $p_-$ goes to the stable node $q_-$. The remaining orbits of the phase portrait are determined where they start and where they end by the type of stability of the equilibrium points and by the Poincaré–Bendixson theorem. Thus the global phase portrait is given in Fig. 35.

**Fig. 36:**
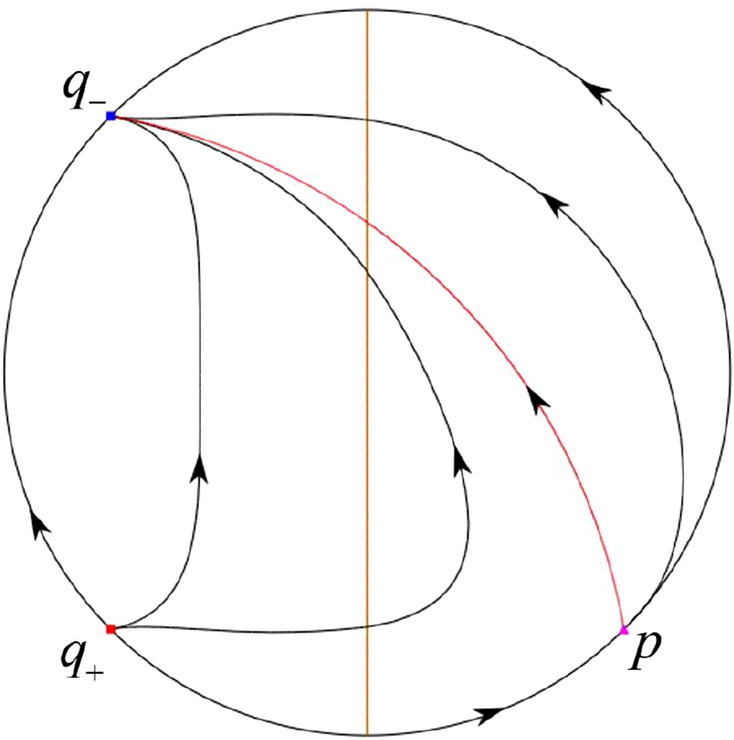
$$S=7,R=2$$

In the case (III-2) an unstable separatrix of the saddle-node *p* goes to the stable node $q_-$. By the type of stability of the equilibrium points and by the Poincaré–Bendixson theorem we get the remaining orbits of the phase portrait. Thus the global phase portrait is given in Fig. 36.

**Fig. 37:**
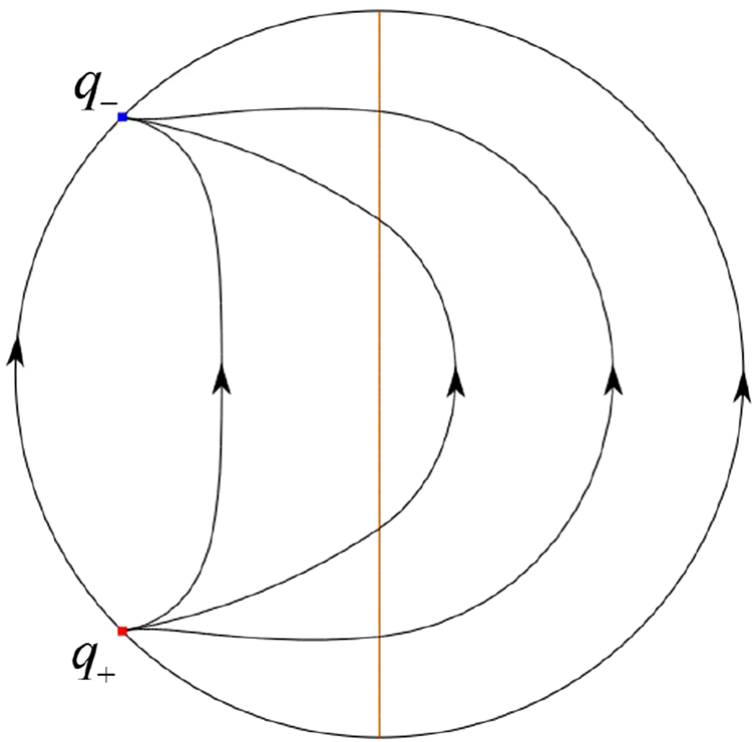
$$S=9,R=1$$

In the case (III-3)–(III-5) there is no separatrices in the phase portrait. All orbits leave $q_+$ for $q_-$. Thus the global phase portrait is shown in Fig. 37.

**Fig. 38:**
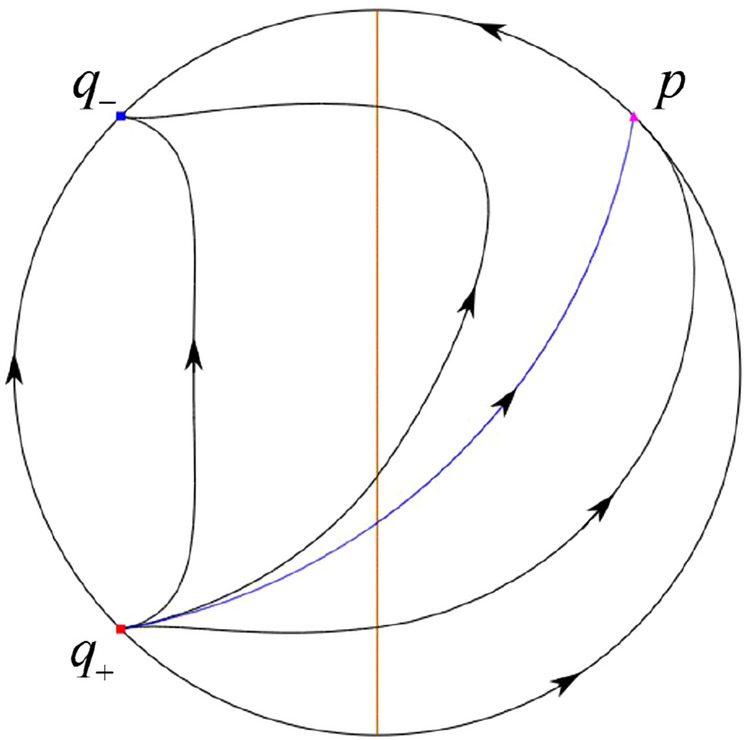
$$S=7,R=2$$

In the case (III-6) a stable separatrix of the saddle-node *p* comes from the unstable node $q_+$. The remaining orbits of the phase portrait are determined by the type of stability of the equilibrium points and by the Poincaré–Bendixson theorem. Thus the global phase portrait is shown in Fig. 38.

**Fig. 39:**
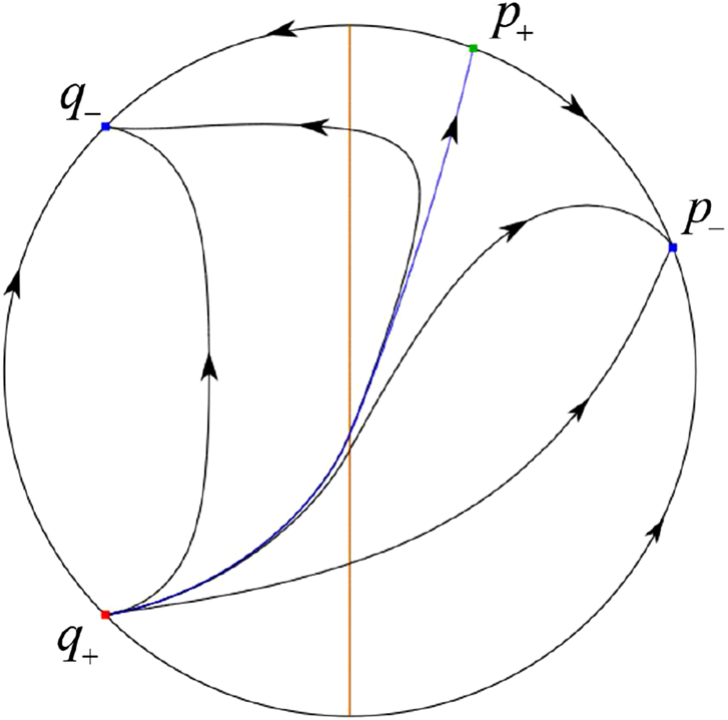
$$S=9,R=2$$

In the case (III-7) a stable separatrix of the saddle $p_+$ comes from the unstable node $q_+$. Similarly we get the remaining orbits of the phase portrait. Thus the global phase portrait is shown in Fig. 39.

**Table 5:** The local phase portraits at the finite and infinite equilibrium points of the continuous piecewise differential systems (IV)

Systems	Conditions	Finite equilibrium points	Infinite equilibrium points
(IV)	(IV-1): $\varsigma <-1$	$P_+$(saddle)	*p*(stable node)
			*O*(unstable node)
		$P_-$(stable node)	*q*(saddle)
			*O*(unstable node)
	(IV-2): $\varsigma =-1$,	$P_+$(saddle)	*p*(stable node)
	$\sigma <0$		*O*(unstable node)
		$$P_-$$(stable node)	
			*O*(semi-hyperbolic saddle-node)
	(IV-3): $\varsigma =-1,$	$P_+$(saddle)	*p*(stable node)
	$\sigma =0$		*O*(unstable node)
		$P_-$(stable node)	*u*-axis(starts an orbit)
			*O*(starts an orbit)
	(IV-4): $\varsigma =-1,$	$P_+$(saddle)	*p*(stable node)
	$\sigma >0$		*O*(unstable node)
		$$P_-$$(stable node)	
			*O*(semi-hyperbolic saddle-node)
	(IV-5): $-1<\varsigma <0$	$P_+$(saddle)	*p*(stable node)
			*O*(unstable node)
		$P_-$(stable node)	*q*(unstable node)
			*O*(saddle)
	(IV-6): $0<\varsigma <1$	$P_+$(unstable node)	*p*(stable node)
			*O*(saddle)
		$P_-$(saddle)	*q*(unstable node)
			*O*(stable node)
	(IV-7): $\varsigma =1,$	$P_+$(unstable node)	
	$\sigma <0$		*O*(semi-hyperbolic saddle-node)
		$P_-$(saddle)	*q*(unstable node)
			*O*(stable node)
	(IV-8): $\varsigma =1,$	$P_+$(unstable node)	*u*-axis(ends an orbit)
	$\sigma =0$		*O*(ends an orbit)
		$P_-$(saddle)	*q*(unstable node)
			*O*(stable node)
	(IV-9): $\varsigma =1,$	$P_+$(unstable node)	
	$\sigma >0$		*O*(semi-hyperbolic saddle-node)
		$P_-$(saddle)	*q*(unstable node)
			*O*(stable node)
	(IV-10): $\varsigma >1$	$P_+$(unstable node)	*p*(saddle)
			*O*(stable node)
		$P_-$(saddle)	*q*(unstable node)
			*O*(stable node)

### Phase Portraits in the Poincaré Disc of Systems (IV)

#### The Finite and Infinite Equilibrium Points

From the proof of Lemma 3 we obtain the results of the finite equilibrium points for systems (IV).

For the infinite equilibrium points we write the differential systems $S_+$ in the local charts $U_1$ and they become

21 $$\begin{aligned} {{\dot{u}}}= \sigma +(\varsigma -1)u+uv, \quad {{\dot{v}}}= -v+v^2; \end{aligned}$$

and in the local chart $U_2$ they become

$$\begin{aligned} {{\dot{u}}}=(1-\varsigma )u-v-\sigma u^2, \quad {{\dot{v}}}=-\varsigma v-\sigma uv. \end{aligned}$$

We separate the study of the infinite equilibrium points of systems $S_+$ in two cases.

*Case* (IV$1_+$): $\varsigma \ne 1$. Then there is only one infinite equilibrium point of systems $S_+$ in the local chart $U_1$, namely $p= (-\sigma /(\varsigma -1),0),$ and the origin *O* of the local chart $U_2$ is an infinite equilibrium point. The eigenvalues of the equilibrium point *p* are $-1$ and $\varsigma -1$. Thus *p* is a stable node if $\varsigma <1$ and a saddle if $\varsigma >1$. The eigenvalues of the equilibrium point *O* are $1-\varsigma$ and $-b$. Then *O* is an unstable node if $\varsigma <0$, a semi-hyperbolic saddle-node if $\varsigma =0$, a saddle if $0<\varsigma <1$, and a stable node $\varsigma >1$.

*Case* (IV$2_+$): $\varsigma =1$. We consider two subcases: $\sigma \ne 0$ and $\sigma =0$. In the first subcase there is no infinite equilibrium point in the local chart $U_1$, but the origin *O* of the local chart $U_2$ is an infinite equilibrium point. Moreover the eigenvalues of *O* are 0 and $-1$, implying that it is a semi-hyperbolic saddle-node. In the second subcase all points on the *u*-axis are infinite equilibrium points in the local chart $V_1$ for systems $S_+$. Since the eigenvalues at each one of these equilibrium points are 0 and $-1\ne 0$, by the normally hyperbolic equilibrium points theorem (see [8]) it follows that at each one of these equilibrium points ends an orbit. The origin of the local chart $U_2$ is also an equilibrium point inside the continuum of equilibrium points at infinity with eigenvalues 0 and $-1$, so the same conclusion for it.

Again we write the differential systems $S_-$ in the local charts $V_1$ and $U_2$. Then in the local chart $V_1$ systems $S_-$ write

22 $$\begin{aligned} {{\dot{u}}}=-\sigma +(\varsigma +1)u+uv, \quad {{\dot{v}}}= v+v^2; \end{aligned}$$

and in the local chart $U_2$ become

$$\begin{aligned} {{\dot{u}}}=-(1+\varsigma )u-v+\sigma u^2, \quad {{\dot{v}}}= -\varsigma v+\sigma uv. \end{aligned}$$

As we did for the systems $S_+$ we separate the study of the infinite equilibrium points of systems $S_-$ in two cases.

*Case* (IV$1_-$): $\varsigma \ne -1$. Then there is only one infinite equilibrium point of systems $S_-$ in the local chart $V_1$, namely $q= (\sigma /(\varsigma +1),0),$ and the origin *O* of the local chart $U_2$ is an infinite equilibrium point. The eigenvalues of the equilibrium point *q* are 1 and $\varsigma +1$. Then *q* is a saddle if $\varsigma <-1$ and an unstable node if $\varsigma >-1$. The eigenvalues of the equilibrium point *O* are $-1-\varsigma$ and $-b$. Then *O* is an unstable node if $\varsigma <-1$, a saddle if $-1<\varsigma <0$, a semi-hyperbolic saddle-node if $\varsigma =0$ and a stable node $\varsigma >0$.

*Case* (IV$2_-$): $\varsigma =-1$. Again we consider two subcases: $\sigma \ne 0$ and $\sigma =0$. In the first subcase there is no infinite equilibrium point in the local chart $U_1$, but the origin *O* of the local chart $U_2$ is an infinite equilibrium point. Moreover the eigenvalues of *O* are 0 and 1, implying that it is a semi-hyperbolic saddle-node. In the second subcase all points on the *u*-axis are infinite equilibrium points in the local chart $V_1$ for systems $S_-$. Since the eigenvalues at each one of these equilibrium points are 0 and $1\ne 0$, by the normally hyperbolic equilibrium points theorem at each one of these equilibrium points starts an orbit. At the origin of the local chart $U_2$ we also have a semi-hyperbolic saddle-node.

In summary from the above discussion, we obtain the results of Table 5.

#### The Global Phase Portraits in the Poincaré Disc

First we see ${{\dot{x}}}=-1$ on the *y*-axis. Then starting at points lying on the *y*-axis all orbits go into the half plane $x<0$. On the other hand the infinity as always is formed by orbits because the equations ${{\dot{v}}}$ of equations (21) and (22) have a common factor *v*. According to Table 5 we divide the study of the global phase portraits in the following cases.

**Fig. 40:**
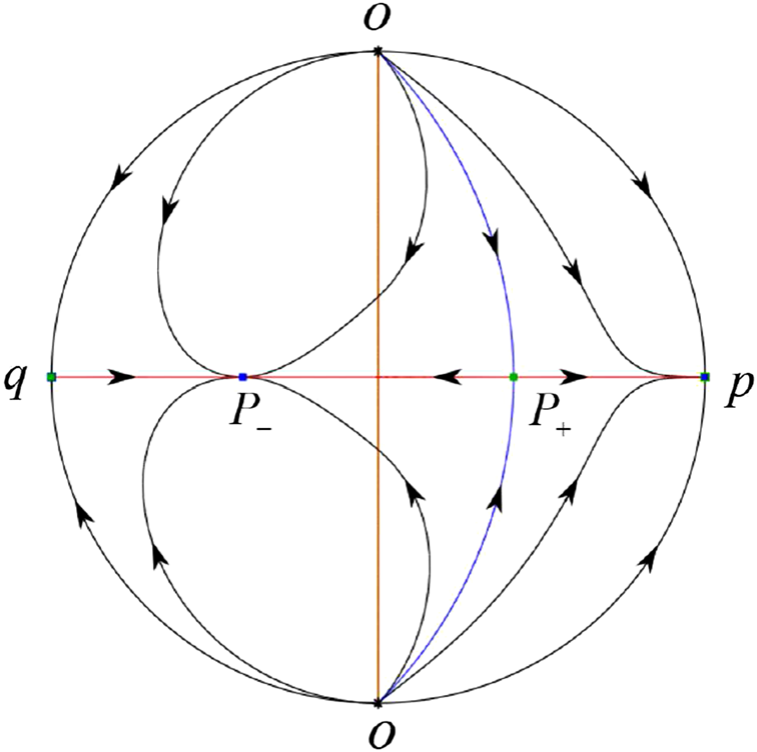
$$S=15,R=4$$

In the case (IV-1) one stable separatrix of the saddle $P_+$ comes from the unstable node *O* in the positive *y*-direction and the second stable separatrix of $P_+$ comes from the unstable node *O* in the negative *y*-direction. One unstable separatrix of $P_+$ goes to the stable node *p* and the second unstable separatrix of $P_+$ goes to the stable node $P_-$. An unstable separatrix of the saddle *q* goes to $P_-$. The remaining orbits of the phase portrait are determined where they start and they end by the type of stability of the equilibrium points and by the Poincaré–Bendixson theorem. Thus the global phase portrait is shown in Fig. 40.

**Fig. 41:**
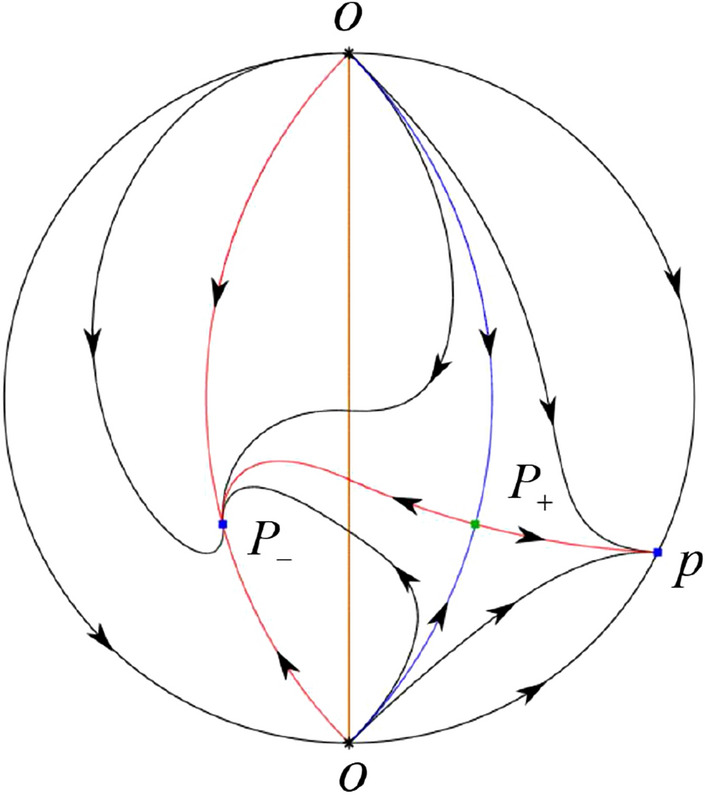
$$S=13,R=4$$

In the case (IV-2) one stable separatrix of the saddle $P_+$ comes from the semi-hyperbolic saddle-node *O* in the positive *y*-direction and the second stable separatrix of $P_+$ comes from the unstable node *O* in the negative *y*-direction. One unstable separatrix of $P_+$ goes to the stable node *p* and the second unstable separatrix of $P_+$ goes to the stable node $P_-$. The remaining orbits of the phase portrait are determined by the type of stability of the equilibrium points and by the Poincaré–Bendixson theorem. Thus the global phase portrait is shown in Fig. 41.

**Fig. 42:**
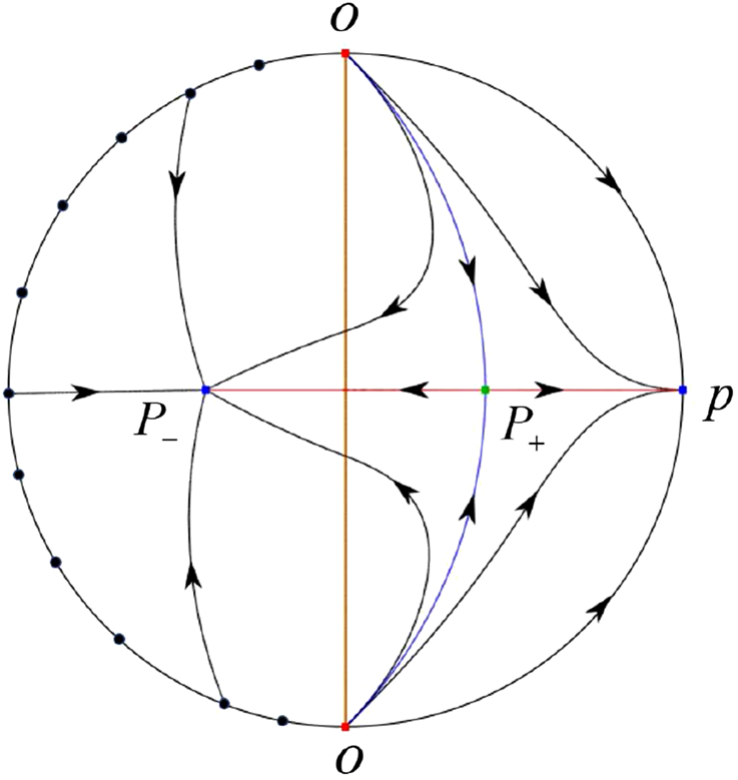
$$S=\infty$$

In the case (IV-3) one stable separatrix of the saddle $P_+$ comes from the unstable node *O* in the positive *y*-direction of the half plane $x\ge 0$ and the second stable separatrix of $P_+$ comes from the equilibrium point *O* in the negative *y*-direction of the half plane $x\ge 0$. One unstable separatrix of $P_+$ goes to the stable node *p* and the second unstable separatrix of $P_+$ goes to the stable node $P_-$. The infinity of $x\le 0$ is filled with equilibrium points. At each one of these infinite equilibrium points starts an orbit going to $P_-$. The remaining orbits of the phase portrait are determined by the type of stability of the equilibrium points and by the Poincaré–Bendixson theorem. Thus the global phase portrait is shown in Fig. 42.

**Fig. 43:**
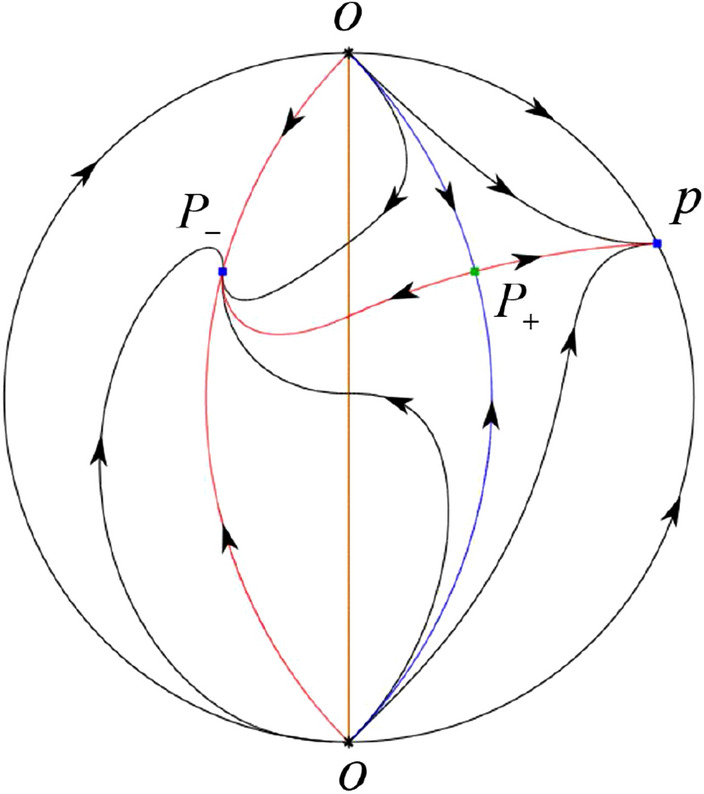
$$S=13,R=4$$

In the case (IV-4) one stable separatrix of the saddle $P_+$ comes from the unstable node *O* in the positive *y*-direction in the half plane $x\ge 0$ and the second stable separatrix of $P_+$ comes from the unstable node *O* in the negative *y*-direction. One unstable separatrix of $P_+$ goes to the stable node *p* and the second unstable separatrix of $P_+$ goes to the stable node $P_-$. An unstable separatrix of the saddle-node *O* in the positive *y*-direction goes to the stable node $P_-$. The remaining orbits of the phase portrait are determined by the type of stability of the equilibrium points and by the Poincaré–Bendixson theorem. Thus the global phase portrait is shown in Fig. 43.

**Fig. 44:**
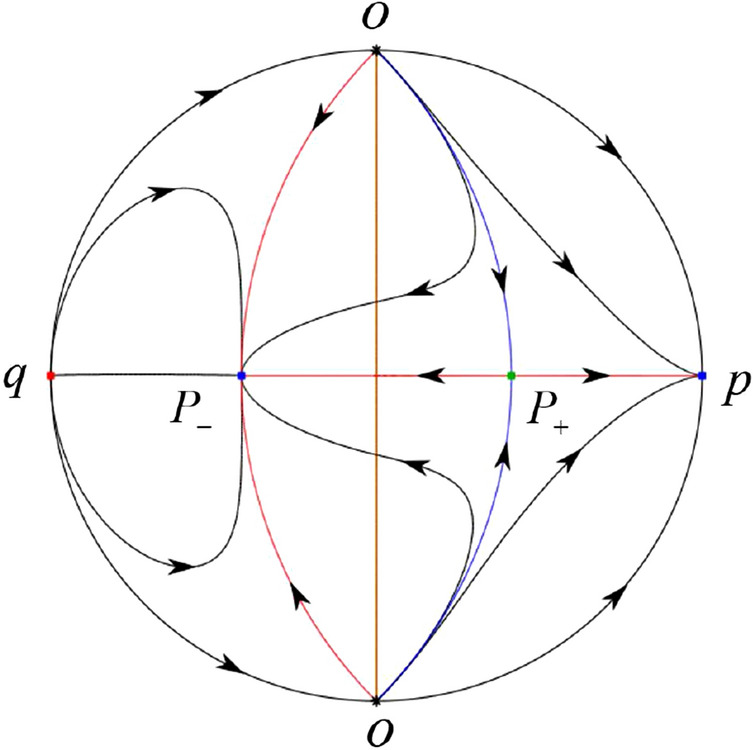
$$S=16,R=5$$

In the case (IV-5) one stable separatrix of the saddle $P_+$ comes from the saddle-node *O* in the positive *y*-direction and the second stable separatrix of $P_+$ comes from the saddle-node *O* in the negative *y*-direction. An unstable separatrix of $P_+$ goes to the stable node *p* and the second unstable separatrix of $P_+$ goes to the stable node $P_-$. One unstable separatrix of the saddle-node *O* in the positive *y*-direction goes to the stable node $P_-$ and an unstable separatrix of the saddle-node *O* in the negative *y*-direction goes to $P_-$. By the type of stability of the equilibrium point and by the Poincaré–Bendixson theorem we get the remaining orbits of the phase portrait. Thus the global phase portrait is shown in Fig. 44.

**Fig. 45:**
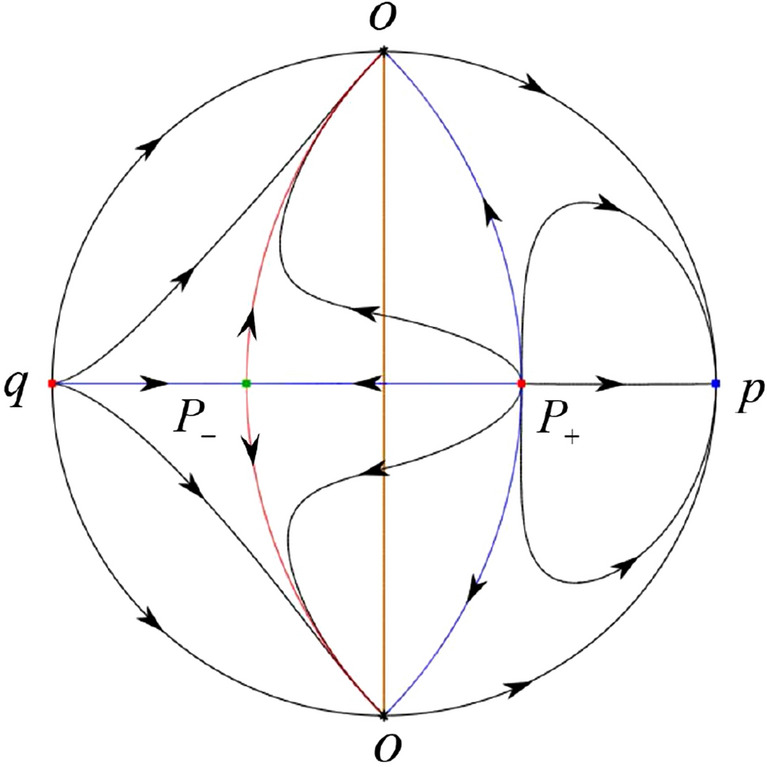
$$S=16,R=5$$

In the case (IV-6) one stable separatrix of the saddle $P_-$ comes from the unstable node *q* and the second stable separatrix of $P_-$ comes from the unstable node $P_+$. One unstable separatrix of $P_-$ goes to the saddle-node *O* in the positive *y*-direction and the second unstable separatrix of $P_-$ goes to the saddle-node *O* in the negative *y*-direction. A stable separatrix of the saddle-node *O* in the positive *y*-direction comes from $P_+$ and a stable separatrix of the saddle-node *O* in the negative *y*-direction comes from $P_+$. Similarly we get the remaining orbits of the phase portrait by the type of stability of the equilibrium points and by the Poincaré–Bendixson theorem. Thus the global phase portrait is shown in Fig. 45.

**Fig. 46:**
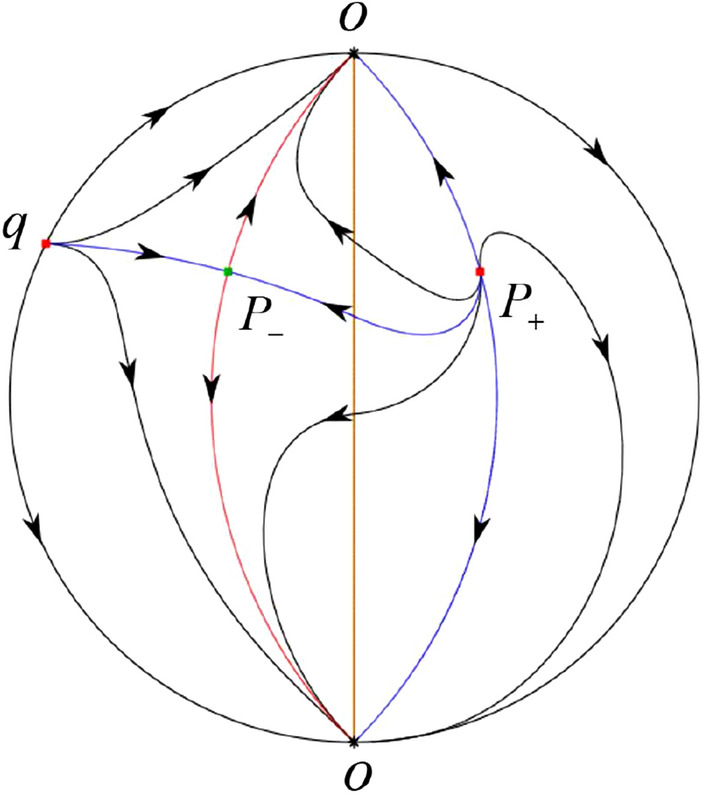
$$S=13,R=4$$

In the case (IV-7) one stable separatrix of the saddle $P_-$ comes from the unstable node *q* and the second stable separatrix of $P_-$ comes from the unstable node $P_+$. One unstable separatrix of $P_-$ goes to the saddle-node *O* in the positive *y*-direction of the half plane $x\le 0$ and the second unstable separatrix of $P_-$ goes to the stable node *O* in the negative *y*-direction. An stable separatrix of the saddle-node *O* in the positive *y*-direction comes from $P_+$. The remaining orbits of the phase portrait are determined by the type of stability of the equilibrium points and by the Poincaré–Bendixson theorem. Thus the global phase portrait is shown in Fig. 46.

**Fig. 47:**
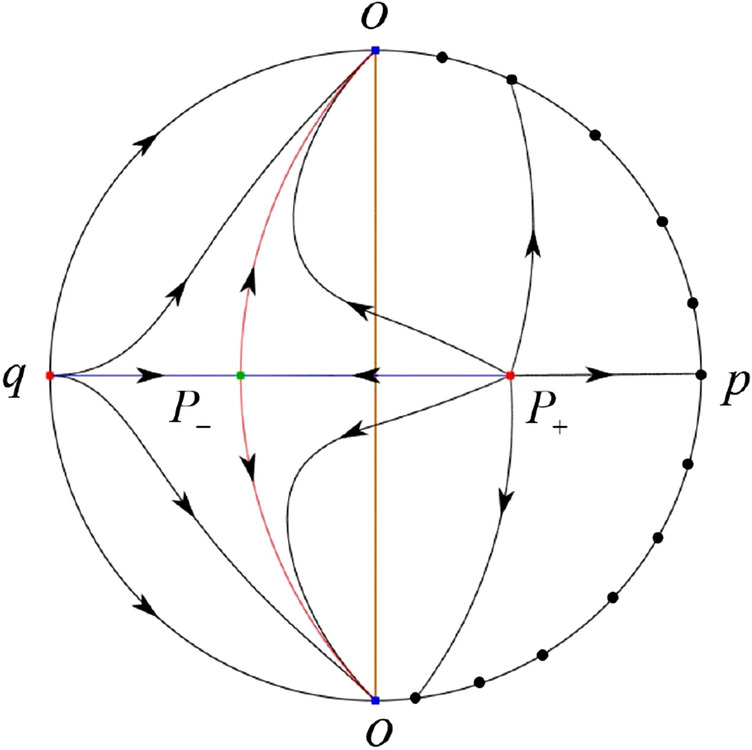
$$S=\infty$$

In the case (IV-8) one stable separatrix of the saddle $P_-$ comes from the unstable node *q* and the second stable separatrix of $P_-$ comes from the unstable node $P_+$. One unstable separatrix of $P_-$ goes to the stable node *O* in the positive *y*-direction of the half plane $x\le 0$ and the second unstable separatrix of $P_-$ goes to the stable node *O* in the negative *y*-direction of the half plane $x\le 0$. The infinity of $x\ge 0$ is filled with equilibrium points. At each one of these infinite equilibrium points arrives an orbit starting at $P_+$. The remaining orbits of the phase portrait are determined by the type of stability of the equilibrium points and by the Poincaré–Bendixson theorem. Thus the global phase portrait is shown in Fig. 47.

**Fig. 48:**
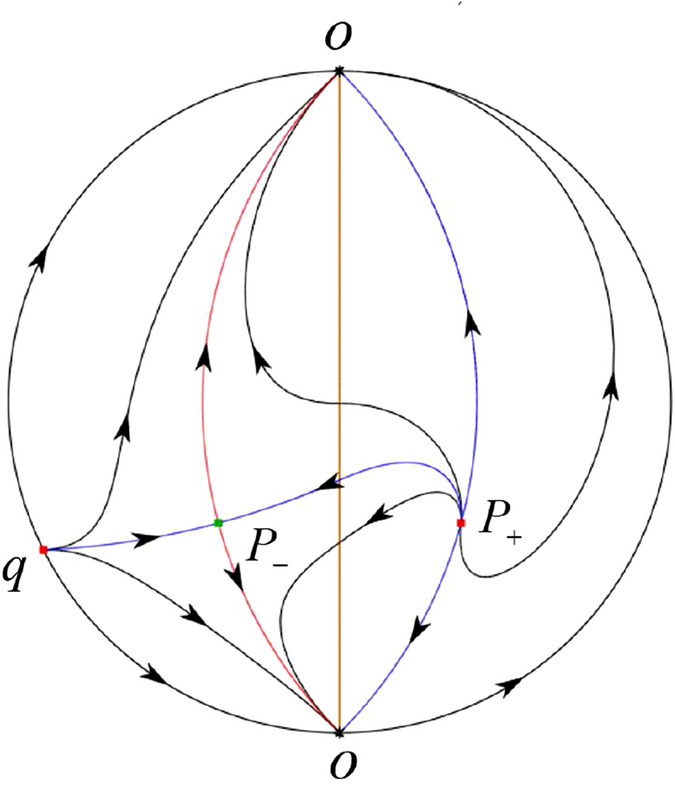
$$S=13,R=4$$

In the case (IV-9) one stable separatrix of the saddle $P_-$ comes from the unstable node *q* and the second stable separatrix of $P_-$ comes from the unstable node $P_+$. One unstable separatrix of $P_-$ goes to the stable node *O* in the positive *y*-direction and the second unstable separatrix of $P_-$ goes to the saddle-node *O* in the negative *y*-direction. A stable separatrix of the saddle-node *O* in the negative *y*-direction comes from $P_+$. Similar to the above, we get the remaining orbits of phase portrait. Thus the global phase portrait is shown in Fig. 48.

**Fig. 49:**
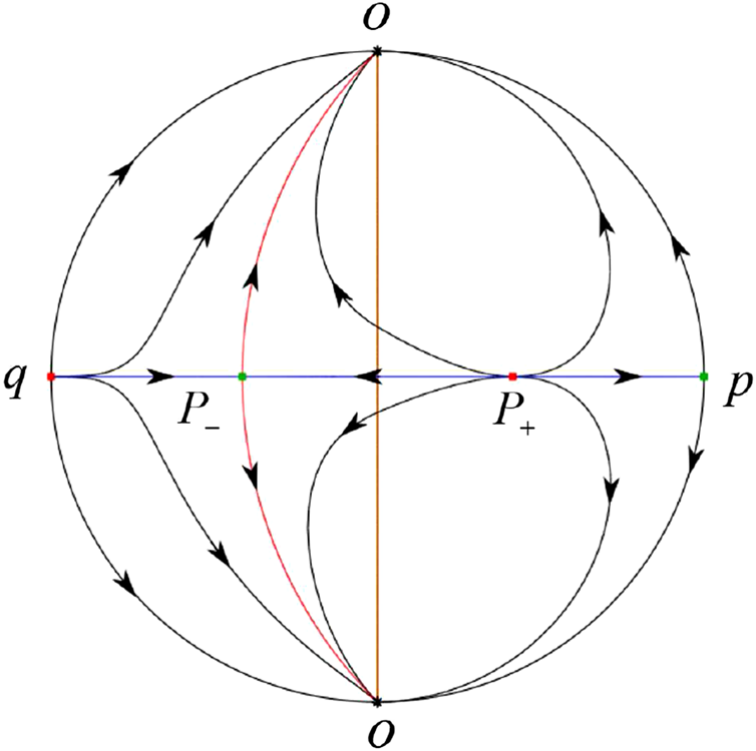
$$S=15,R=4$$

In the case (IV-10) one stable separatrix of the saddle $P_-$ comes from the unstable node *q* and the second stable separatrix of $P_-$ comes from the unstable node $P_+$. One unstable separatrix of $P_-$ goes to the stable node *O* in the positive *y*-direction and the second unstable separatrix of $P_-$ goes to the stable node *O* in the negative *y*-direction. A stable separatrix of the saddle *p* comes from $P_+$. Similarly we get the remaining orbits of the phase portrait by the type of stability of the equilibrium points and by the Poincaré–Bendixson theorem. Thus the global phase portrait is shown in Fig. 49.

### Phase Portraits in the Poincaré Disc of Systems (V)

#### The Finite and Infinite Equilibrium Points

From the proof of Lemma 3 we see that systems $S_+$ have the virtual equilibrium points $P_+=(-1,\sigma /\varsigma )$ and systems $S_-$ have the virtual equilibrium point $P_-=(1,\sigma /\varsigma )$.

Note that systems $S_+$ of systems (V) are the same that systems $S_-$ of systems (IV) if we regard $\sigma$ as $-\sigma$. While systems $S_-$ of systems (V) are also the same that systems $S_+$ of systems (IV). So from the results of systems (IV) for the finite and infinite equilibrium points of systems (V) we get Table 6.

#### The Global Phase Portraits in the Poincaré Disc

According to Table 6 we below discuss the global phase portraits in the following cases.

**Fig. 50:**
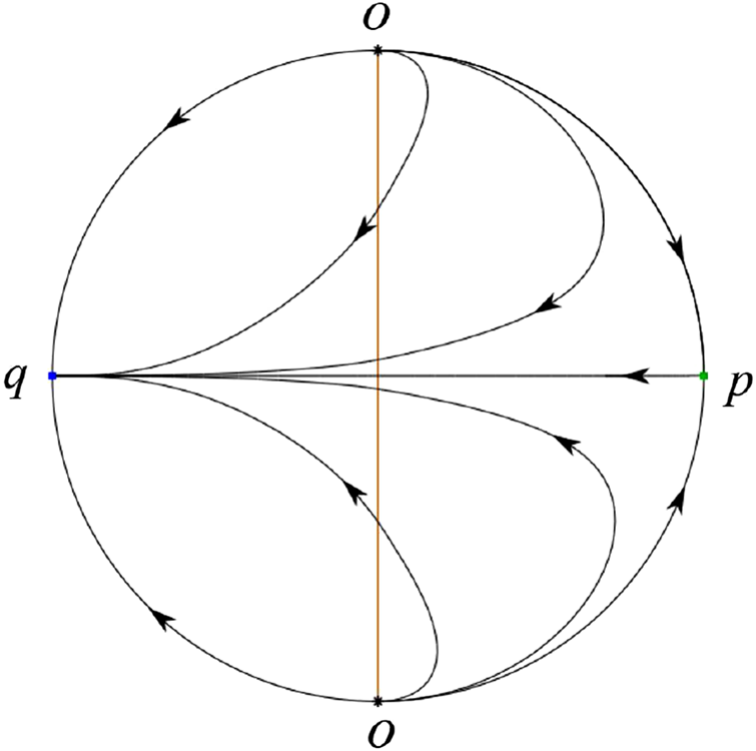
$$S=9,R=2$$

In the case (V-1) an unstable separatrix of the saddle *p* goes to the stable node *q*. The remaining orbits of the phase portrait are determined where they start and they end by the type of stability of the equilibrium points and by the Poincaré–Bendixson theorem. Thus the global phase portrait is shown in Fig. 50.

**Fig. 51:**
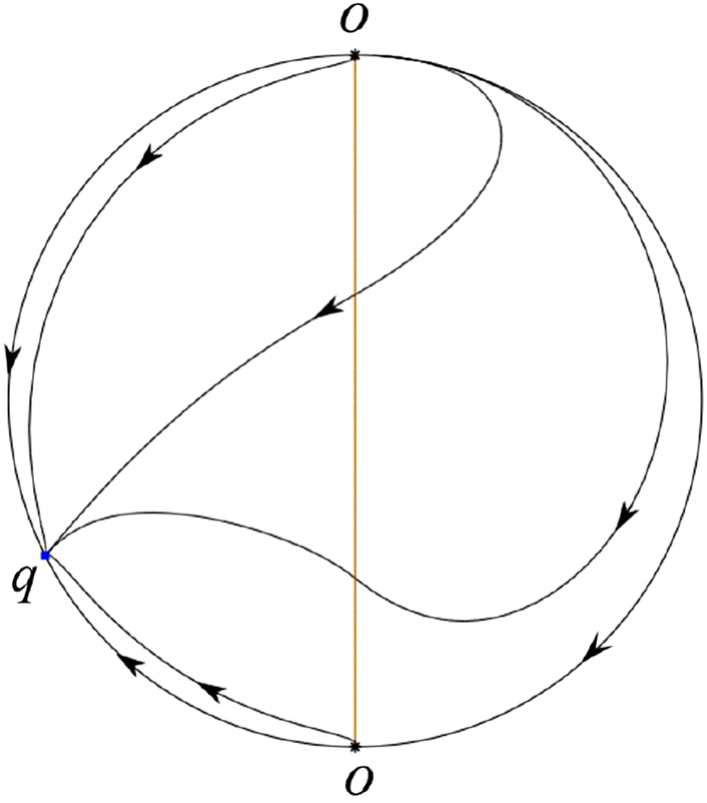
$$S=7,R=2$$

In the case (V-2) an unstable separatrix comes from the saddle-node *O* in the negative *y*-direction and goes to the stable node *q*. The remaining orbits of the phase portrait are determined by the type of stability of the equilibrium points and by the Poincaré–Bendixson theorem. Thus the global phase portrait is shown in Fig. 51.

**Fig. 52:**
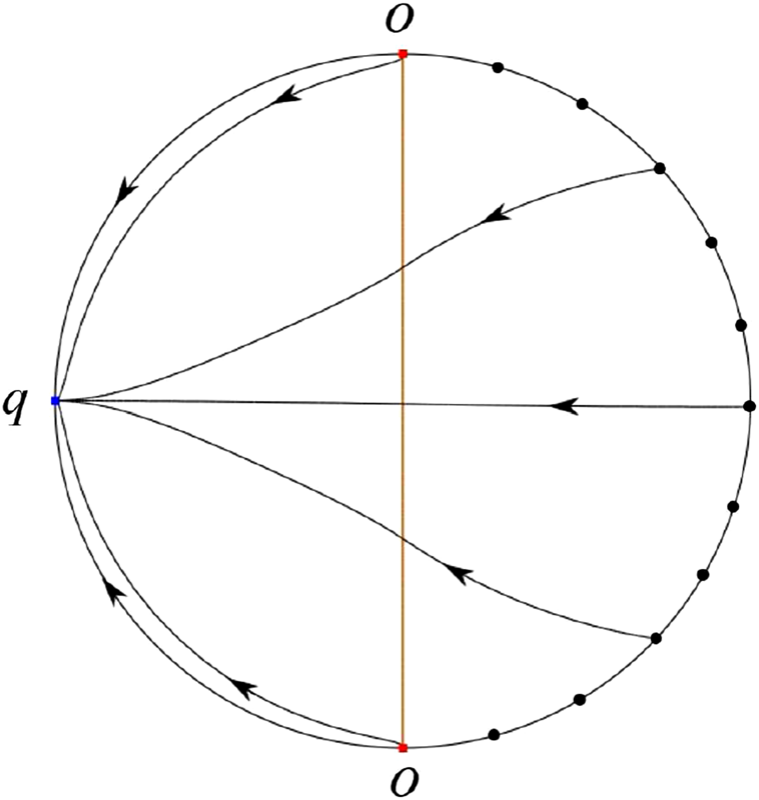
$$S=\infty$$

In the case (V-3) the infinity of $x\ge 0$ is filled with equilibrium points. At each one of these infinite equilibrium points starts an orbit going to the stable node *q*. Thus the global phase portrait is shown in Fig. 52.

**Fig. 53:**
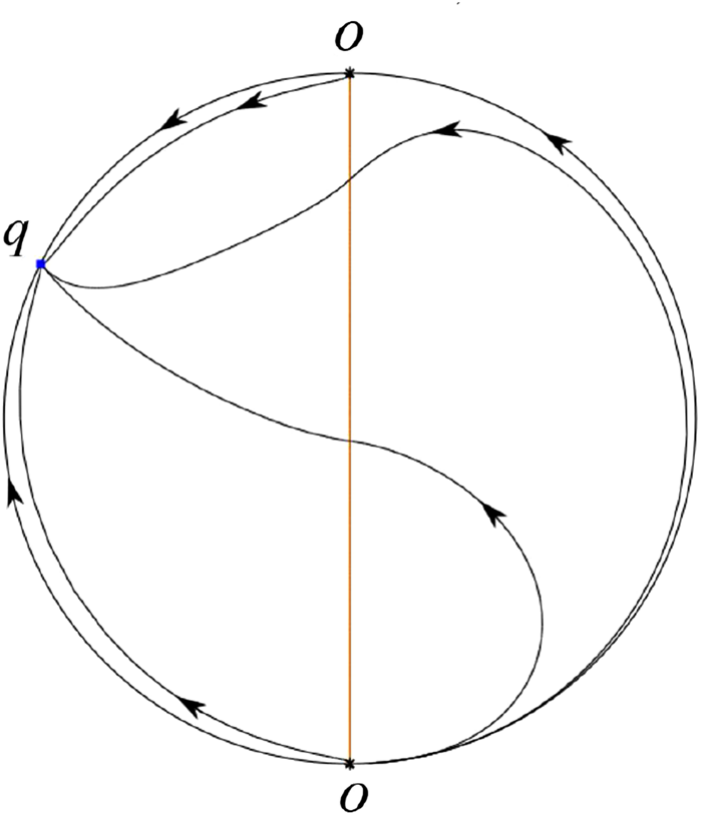
$$S=7,R=2$$

In the case (V-4) an unstable separatrix comes from the saddle-node *O* in the negative *y*-direction and goes to the stable node *q*. We similarly get the remaining orbits of the phase portrait by the type of stability of the equilibrium points and by the Poincaré–Bendixson theorem. Thus the global phase portrait is shown in Fig. 53.

**Fig. 54:**
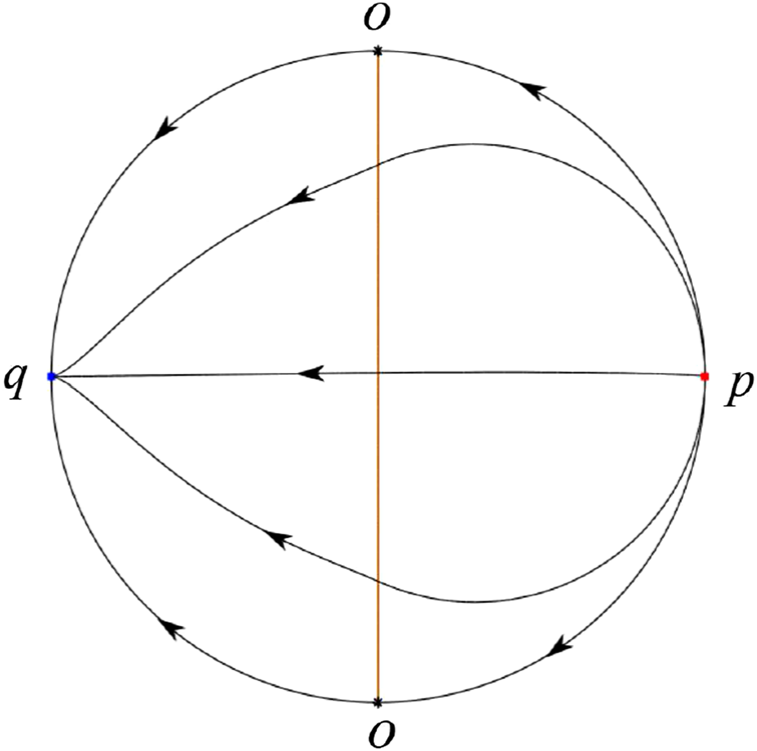
$$S=10,R=3$$

In the case (V-5) an unstable separatrix comes from the saddle-node *O* in the positive *y*-direction and goes to the stable node *q*. An unstable separatrix comes from the saddle-node *O* in the negative *y*-direction and goes to *q*. The remaining orbits in the phase portrait are determined by the type of stability of the equilibrium points and by the Poincaré–Bendixson theorem. Thus the global phase portrait is shown in Fig. 54.

**Fig. 55:**
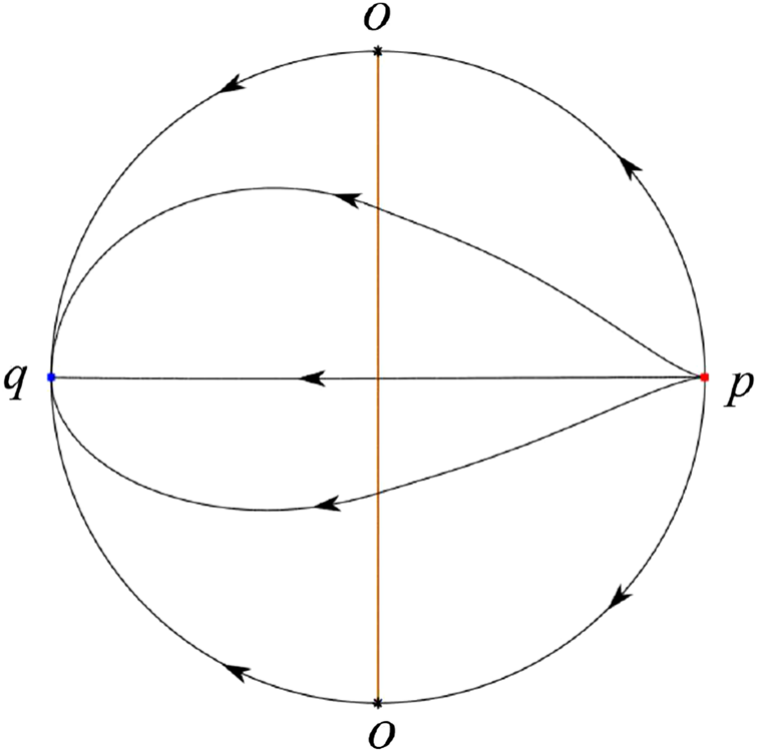
$$S=10,R=3$$

In the case (V-6) an unstable separatrix comes from the unstable node *p* and goes to the saddle-node *O* in the positive *y*-direction. Another unstable separatrix comes from *p* and goes to the saddle-node *O* in the negative *y*-direction. The remaining orbits in the phase portrait are determined by the type of stability of the equilibrium points and by the Poincaré–Bendixson theorem. Thus the global phase portrait is shown in Fig. 55.

**Fig. 56:**
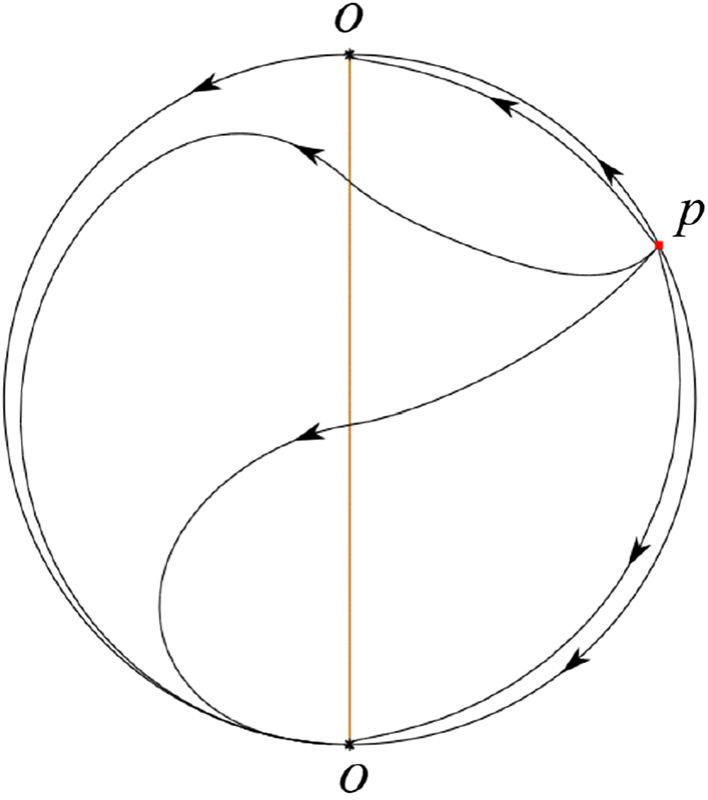
$$S=7,R=2$$

In the case (V-7) an unstable separatrix comes from the unstable node *p* and goes to the saddle-node *O* in the positive *y*-direction. We get the remaining orbits by the type of stability of the equilibrium points and by the Poincaré–Bendixson theorem. Thus the global phase portrait is shown in Fig. 56.

**Fig. 57:**
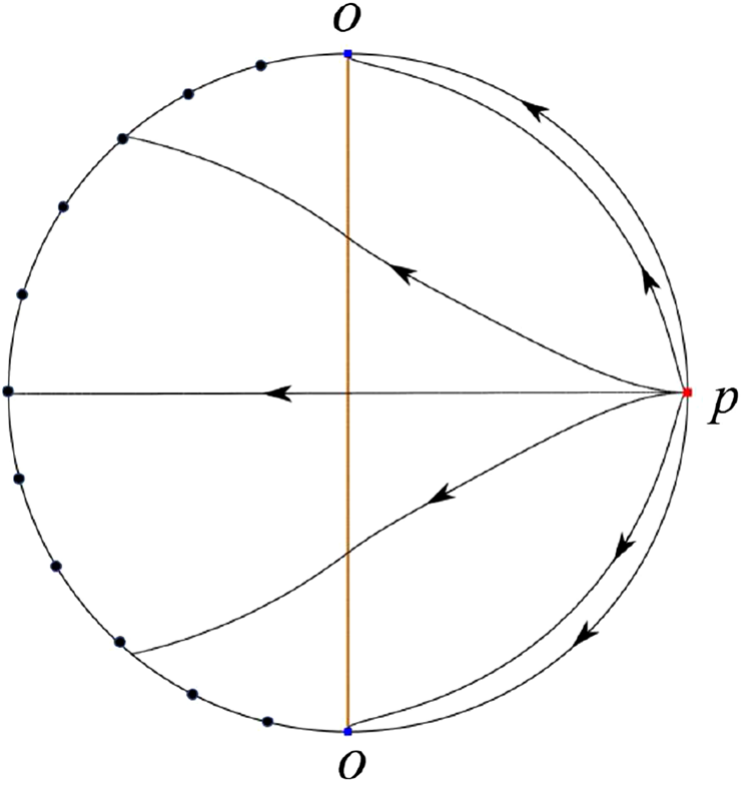
$$S=\infty$$

In the case (V-8) the infinity of the half plane $x\le 0$ is filled with equilibrium point. At each one of these infinite equilibrium points arrives an orbit starting at the unstable node *p*. Thus the global phase portrait is shown in Fig. 57.

**Fig. 58:**
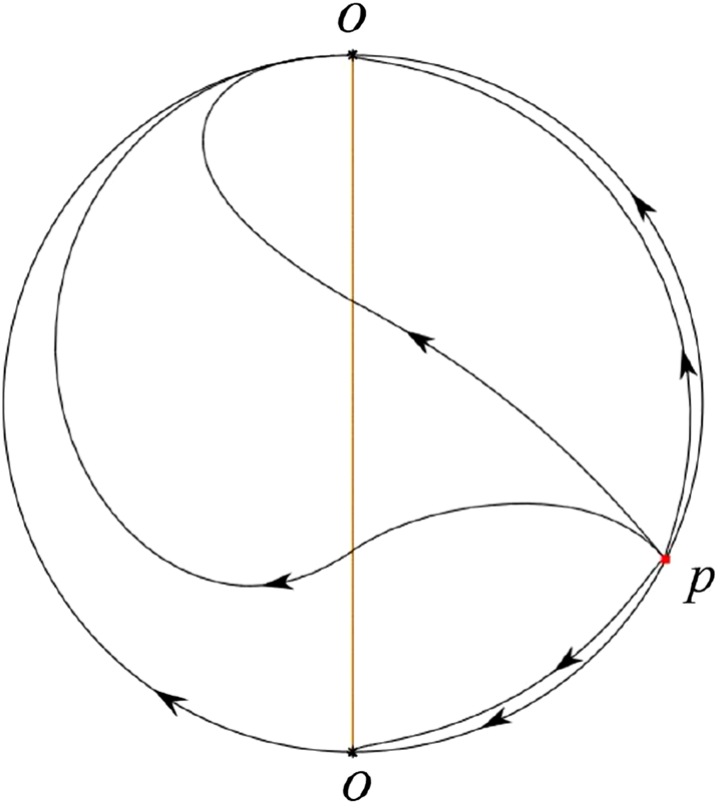
$$S=7,R=2$$

In the case (V-9) an unstable separatrix comes from the unstable node *p* and goes to the saddle-node *O* in the negative *y*-direction. We obtain the remaining orbits by the type of stability of the equilibrium points and by the Poincaré–Bendixson theorem. Thus the global phase portrait is shown in Fig. 58.

**Fig. 59:**
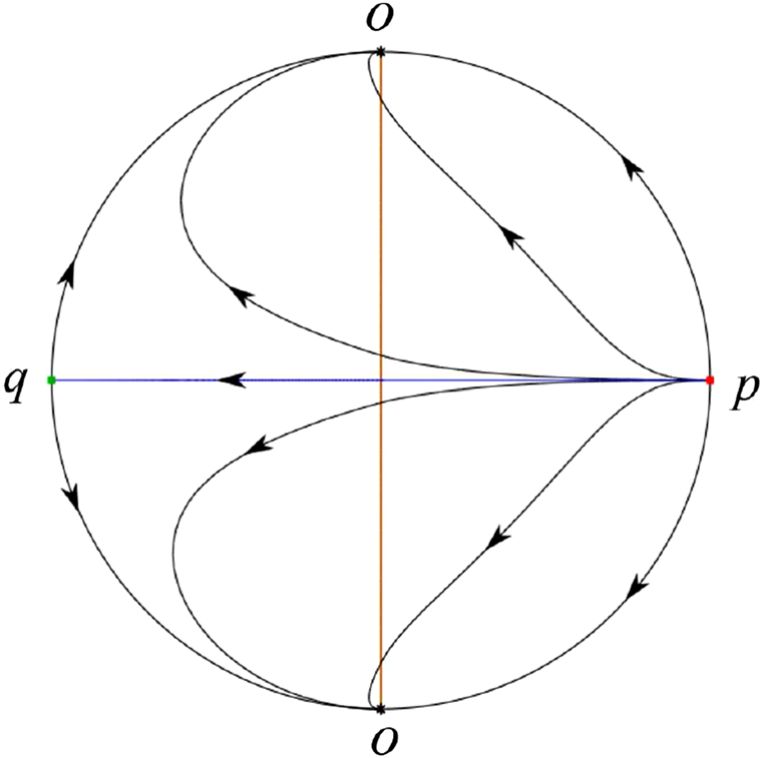
$$S=9,R=2$$

In the case (V-10) a stable separatrix of the saddle *q* come from the unstable node *p*. We similarly get the remaining orbits by the type of stability of the equilibrium points and by the Poincaré–Bendixson theorem. Thus the global phase portrait is shown in Fig. 59.

**Table 6:** The local phase portraits at the finite and infinite equilibrium points of the continuous piecewise differential systems (V)

Systems	Conditions	Finite equilibrium points	Infinite equilibrium points
(V)	(V-1): $\varsigma <-1$	$P_+$(stable node)	*p*(saddle)
			*O*(unstable node)
		$P_-$(saddle)	*q*(stable node)
			*O*(unstable node)
	(V-2): $\varsigma =-1,$	$P_+$(stable node)	
	$\sigma <0$		*O*(semi-hyperbolic saddle-node)
		$P_-$(saddle)	*q*(stable node)
			*O*(unstable node)
	(V-3): $\varsigma =-1,$	$P_+$(stable node)	*u*-axis(starts an orbit)
	$\sigma =0$		*O*(starts an orbit)
		$P_-$(saddle)	*q*(stable node)
			*O*(unstable node)
	(V-4): $\varsigma =-1,$	$P_+$(stable node)	
	$\sigma >0$		*O*(semi-hyperbolic saddle-node)
		$P_-$(saddle)	*q*(stable node)
			*O*(unstable node)
	(V-5): $-1<\varsigma <0$	$P_+$(stable node)	*p*(unstable node)
			*O*(saddle)
		$P_-$(saddle)	*q*(stable node)
			*O*(unstable node)
	(V-6): $0<\varsigma <1$	$P_+$(saddle)	*p*(unstable node)
			*O*(stable node)
		$P_-$(unstable node)	*q*(stable node)
			*O*(saddle)
	(V-7): $\varsigma =1,$	$P_+$(saddle)	*p*(unstable node)
	$\sigma <0$		*O*(stable node)
		$P_-$(unstable node)	
			*O*(semi-hyperbolic saddle-node)
	(V-8): $\varsigma =1,$	$P_+$(saddle)	*p*(unstable node)
	$\sigma =0$		*O*(stable node)
		$P_-$(unstable node)	*u*-axis(ends an orbit)
			*O*(ends an orbit)
	(V-9): $\varsigma =1,$	$P_+$(saddle)	*p*(unstable node)
	$\sigma >0$		*O*(stable node)
		$P_-$(unstable node)	
			*O*(semi-hyperbolic saddle-node)
	(V-10): $\varsigma >1$	$P_-$(saddle)	*p*(unstable node)
			*O*(stable node)
		$P_+$(unstable node)	*q*(saddle)
			*O*(stable node)

### Phase Portraits in the Poincaré Disc of Systems (*VI*)

#### The Finite and Infinite Equilibrium Points

As it is given in the proof of Lemma 3 we obtain the results of the finite equilibrium points for systems (VI).

For the infinite equilibrium points we write the differential systems $S_+$ in the local charts $U_1$ and become

$$\begin{aligned} {{\dot{u}}}= \sigma +(\varsigma -1)u, \quad {{\dot{v}}}= -v; \end{aligned}$$

and in the local chart $U_2$ become

$$\begin{aligned} {{\dot{u}}}=(1-\varsigma )u-\sigma u^2, \quad {{\dot{v}}}=-\varsigma v-\sigma uv. \end{aligned}$$

We separate the study of the infinite equilibrium points of systems $S_+$ in two cases.

*Case* (VI$1_+$): $\varsigma \ne 1$. Then there is only one infinite equilibrium point of systems $S_+$ in the local chart $U_1$, namely $p= (-\sigma /(\varsigma -1),0)$ and the origin *O* of the local chart $U_2$ is an infinite equilibrium point. The eigenvalues of the equilibrium point *p* are $-1$ and $\varsigma -1$. Thus *p* is a stable node if $\varsigma <1$, and a saddle if $\varsigma >1$. The eigenvalues of the equilibrium point *O* are $1-\varsigma$ and $-b$. Then *O* is an unstable node if $\varsigma <0$, a semi-hyperbolic saddle-node if $\varsigma =0$, a saddle if $0<\varsigma <1$ and a stable node $\varsigma >1$.

*Case* (VI$2_+$): $\varsigma =1$. We consider two subcases: $\sigma \ne 0$ and $\sigma =0$. In the first subcase there is no infinite equilibrium point in the local chart $U_1$, but the origin *O* of the local chart $U_2$ is an infinite equilibrium point. Moreover the eigenvalues of *O* are 0 and $-1$, implying that it is a semi-hyperbolic saddle-node. In the second subcase all points on the *u*-axis are infinite equilibrium points in the local chart $V_1$ for systems $S_+$. Since the eigenvalues at each one of these equilibrium points are 0 and $-1\ne 0$, it follows that at each one of these equilibrium points ends an orbit. The origin of the local chart $U_2$ is also an equilibrium point inside the continuum of equilibrium points at infinity with eigenvalues 0 and $-1$, so the same conclusion for it.

Again we write the differential systems $S_-$ in the local charts $V_1$ and $U_2$. Then in the local chart $V_1$ systems $S_-$ write

$$\begin{aligned} {{\dot{u}}}=-\sigma +(\varsigma +1)u, \quad {{\dot{v}}}= v; \end{aligned}$$

and in the local chart $U_2$ become

$$\begin{aligned} {{\dot{u}}}=-(1+\varsigma )u+\sigma u^2, \quad {{\dot{v}}}= -\varsigma v+\sigma uv. \end{aligned}$$

As we did for the systems $S_+$ we separate the study of the infinite equilibrium points of systems $S_-$ in two cases.

*Case* (VI$1_-$): $\varsigma \ne -1$. Then there is only one infinite equilibrium point of systems $S_-$ in the local chart $V_1$, namely $q= (\sigma /(\varsigma +1),0)$ and the origin *O* of the local chart $U_2$ is an infinite equilibrium point. The eigenvalues of the equilibrium point *q* are 1 and $\varsigma +1$. Then *q* is a saddle if $\varsigma <-1$ and an unstable node if $\varsigma >-1$. The eigenvalues of the equilibrium point *O* are $-1-\varsigma$ and $-b$. Then *O* is an unstable node if $\varsigma <-1$, a saddle if $-1<\varsigma <0$, a semi-hyperbolic saddle-node if $\varsigma =0$ and a stable node $\varsigma >0$.

*Case* (VI$2_-$): $\varsigma =-1$. Again we consider two subcases: $\sigma \ne 0$ and $\sigma =0$. In the first subcase there is no infinite equilibrium point in the local chart $U_1$, but the origin *O* of the local chart $U_2$ is an infinite equilibrium point. Moreover the eigenvalues of *O* are 0 and 1, implying that it is a semi-hyperbolic saddle-node. In the second subcase all points on the *u*-axis are infinite equilibrium points in the local chart $V_1$ for systems $S_-$. Since the eigenvalues at each one of these equilibrium points are 0 and $1\ne 0$, at each one of these equilibrium points starts an orbit. At the origin of the local chart $U_2$ we also have a semi-hyperbolic saddle-node.

In summary from the above discussion, we obtain the results of Table 7.

**Table 7:** The local phase portraits at the finite and infinite equilibrium points of the continuous piecewise differential systems (VI)

Systems	Conditions	Finite equilibrium points	Infinite equilibrium points
(VI)	(VI-1): $\varsigma <-1$	*P*(saddle)	*p*(stable node)
			*O*(unstable node)
		*P*(stable node)	*q*(saddle)
			*O*(unstable node)
	(VI-2): $\varsigma =-1,$	*P*(saddle)	*p*(stable node)
	$\sigma <0$		*O*(unstable node)
		*P*(stable node)	
			*O*(semi-hyperbolic saddle-node)
	(VI-3): $\varsigma =-1,$	*P*(saddle)	*p*(stable node)
	$\sigma =0$		*O*(unstable node)
		*P*(stable node)	*u*-axis(starts an orbit)
			*O*(starts an orbit)
	(VI-4): $\varsigma =-1,$	*P*(saddle)	*p*(stable node)
	$\sigma >0$		*O*(unstable node)
		*P*(stable node)	
			*O*(semi-hyperbolic saddle-node)
	(VI-5): $-1<\varsigma <0$	*P*(saddle)	*p*(stable node)
			*O*(unstable node)
		*P*(stable node)	*q*(unstable node)
			*O*(saddle)
	(VI-6): $0<\varsigma <1$	*P*(unstable node)	*p*(stable node)
			*O*(saddle)
		*P*(saddle)	*q*(unstable node)
			*O*(stable node)
	(VI-7): $\varsigma =1,$	*P*(unstable node)	
	$\sigma <0$		*O*(semi-hyperbolic saddle-node)
		*P*(saddle)	*q*(unstable node)
			*O*(stable node)
	(VI-8): $\varsigma =1,$	*P*(unstable node)	*u*-axis(ends an orbit)
	$\sigma =0$		*O*(ends an orbit)
		*P*(saddle)	*q*(unstable node)
			*O*(stable node)
	(VI-9): $\varsigma =1,$	*P*(unstable node)	
	$\sigma >0$		*O*(semi-hyperbolic saddle-node)
		*P*(saddle)	*q*(unstable node)
			*O*(stable node)
	(VI-10): $\varsigma >1$	*P*(unstable node)	*p*(saddle)
			*O*(stable node)
		*P*(saddle)	*q*(unstable node)
			*O*(stable node)

#### The Global Phase Portraits in the Poincaré Disc

Note that ${{\dot{x}}}=0$ and ${{\dot{y}}}=\varsigma y$ when $x=0$. This implies that the *y*-axis is invariant, i.e., the *y*-axis is formed by orbits. According to Table 7 we divide the study of the global phase portraits in the following cases.

**Fig. 60:**
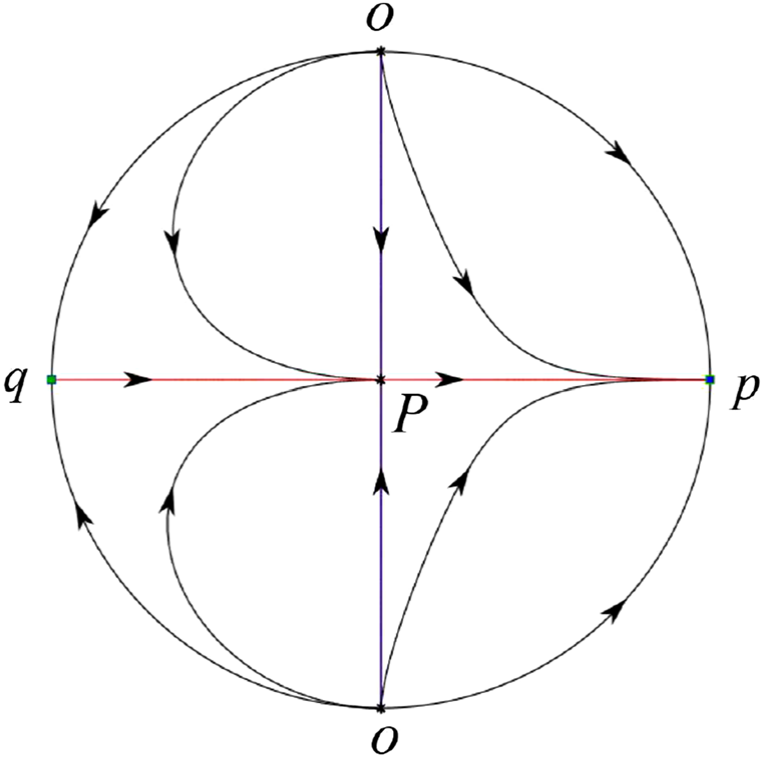
$$S=13,R=4$$

In the case (VI-1) one stable separatrix of the saddle-node *P* lying on the positive *y*-axis comes from the unstable node *O* in the positive *y*-direction, while the second stable separatrix of *P* lying on the negative *y*-axis comes from the unstable node *O* in the negative *y*-direction. The unstable separatrix of the saddle *q* goes to *P*. The unstable separatrix of *P* goes to the stable node *p*. The remaining orbits of the phase portrait are determined where they start and they end by the type of stability of the equilibrium points and by the Poincaré–Bendixson theorem. Thus the global phase portrait is shown in Fig. 60.

**Fig. 61:**
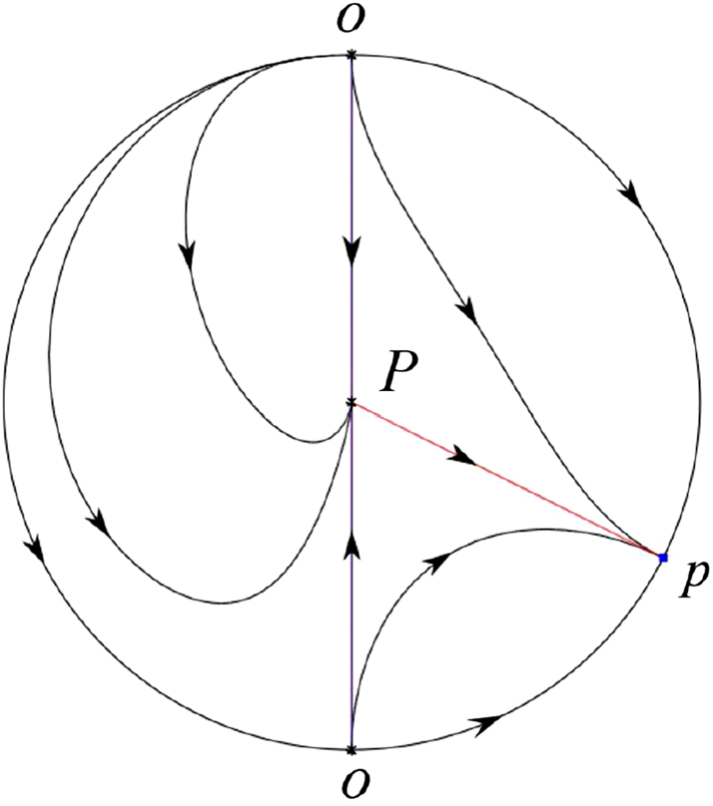
$$S=10,R=3$$

In the case (VI-2) one stable separatrix of *P* comes from the unstable node *O* in the positive *y*-direction and the second stable separatrix of *P* comes from the saddle-node *O* in the negative *y*-direction. An unstable separatrix of *P* goes to the stable node *p*. The remaining orbits of the phase portrait are determined by the type of stability of the equilibrium points and by the Poincaré–Bendixson theorem. Thus the global phase portrait is shown in Fig. 61.

**Fig. 62:**
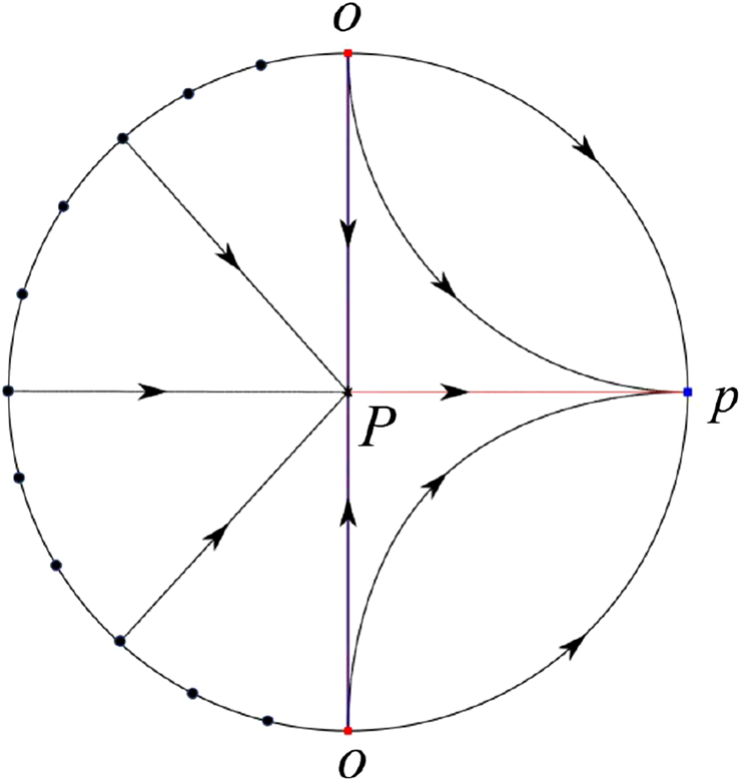
$$S=\infty$$

In the case (VI-3) one stable separatrix of *P* lying on the positive *y*-axis comes from the unstable node *O* in the positive *y*-direction of the half plane $x\ge 0$ and the second stable separatrix of *P* lying on the negative *y*-axis comes from the unstable node *O* in the negative *y*-direction of the half plane $x\ge 0$. An unstable separatrix of the saddle *P* in the half plane $x\ge 0$ goes to the stable node *p*. The infinity of $x\le 0$ is filled with equilibrium points. At each one of these equilibrium points starts an orbit going to the stable node *P* in the half plane $x\le 0$. The remaining orbits of the phase portrait are determined by the type of stability of the equilibrium points and by the Poincaré–Bendixson theorem. Thus the global phase portrait is shown in Fig. 62.

**Fig. 63:**
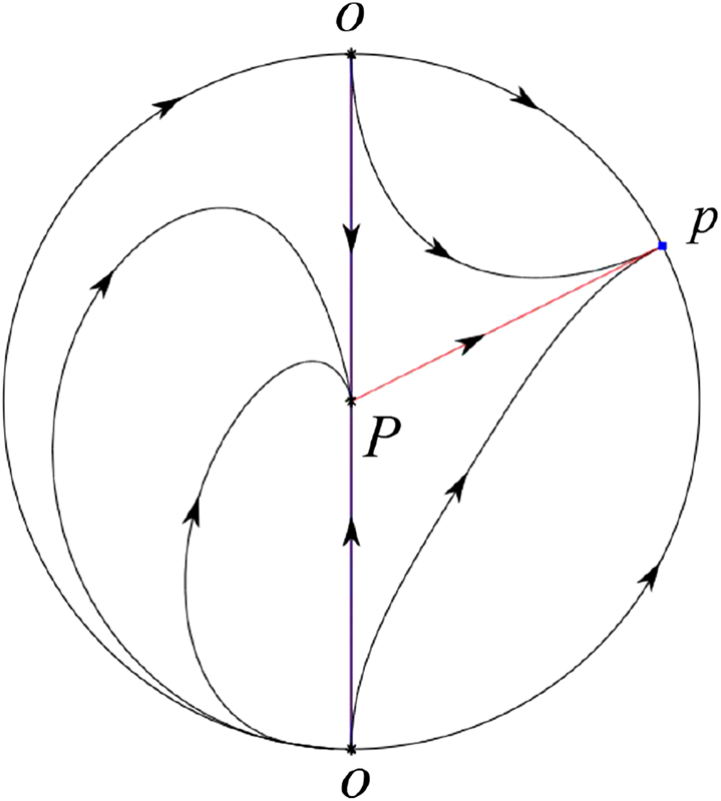
$$S=10,R=3$$

In the case (VI-4) one stable separatrix of *P* comes from the saddle-node *O* in the positive *y*-direction and the second stable separatrix of *P* comes from the unstable node *O* in the negative *y*-direction. An unstable separatrix of *P* goes to the stable node *p*. The remaining orbits of the phase portrait are determined by the type of stability of the equilibrium points and by the Poincaré–Bendixson theorem. Thus the global phase portrait is shown in Fig. 63.

**Fig. 64:**
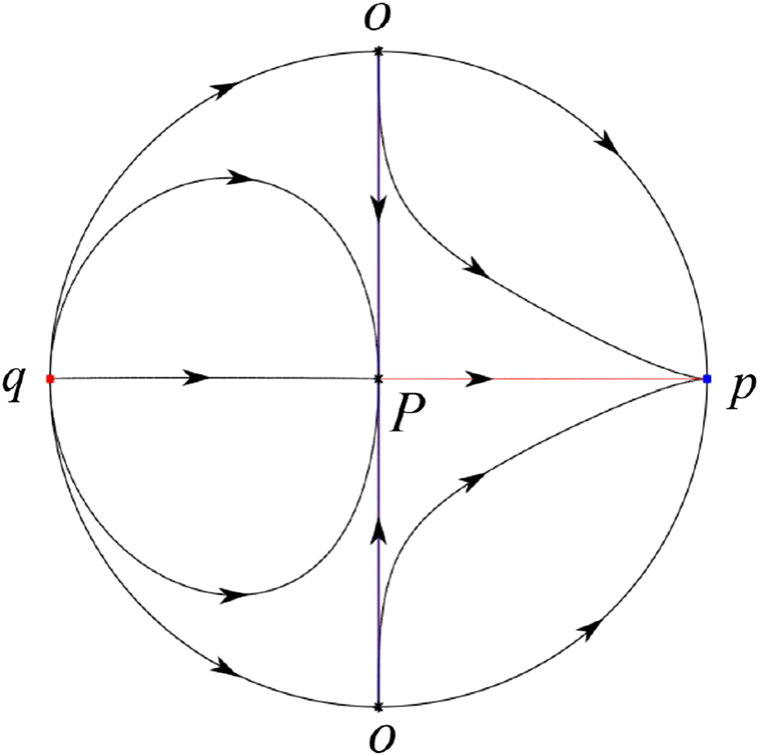
$$S=12,R=3$$

In the case (VI-5) one stable separatrix of *P* comes from the saddle-node *O* in the positive *y*-direction and the second stable separatrix of *P* comes from the saddle-node *O* in the negative *y*-direction. An unstable separatrix of *P* goes to the stable node *p*. The remaining orbits of the phase portrait are determined by the type of stability of the equilibrium points and by the Poincaré–Bendixson theorem. Thus the global phase portrait is shown in Fig. 64.

**Fig. 65:**
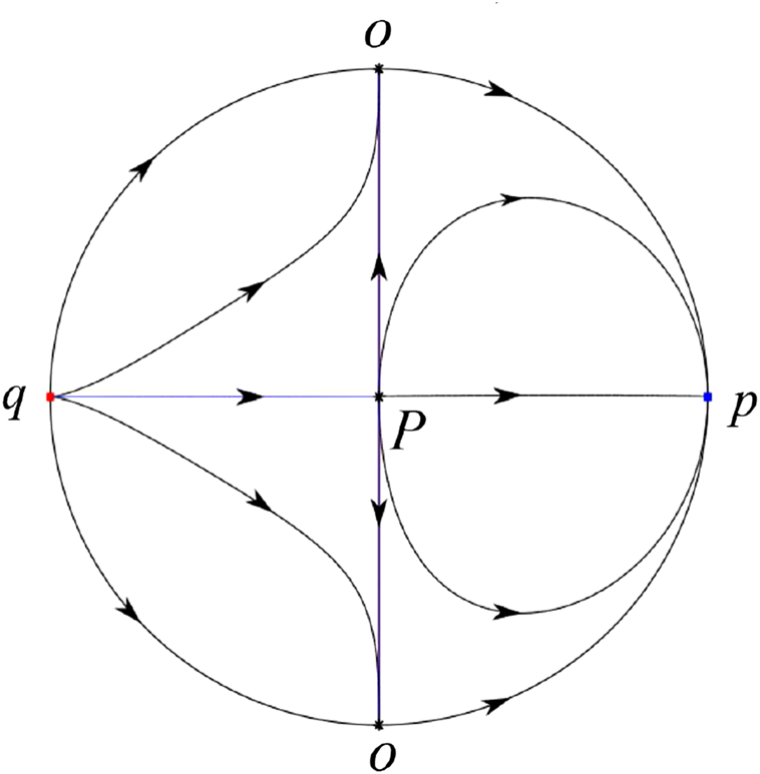
$$S=12, R=3$$

In the case (VI-6) one unstable separatrix of *P* goes to the saddle-node *O* in the positive *y*-direction and the second unstable separatrix of *P* goes to the saddle-node *O* in the negative *y*-direction. A stable separatrix of *P* comes from the unstable node *q*. The remaining orbits of the phase portrait are determined by the type of stability of the equilibrium points and by the Poincaré–Bendixson theorem. Thus the global phase portrait is shown in Fig. 65.

**Fig. 66:**
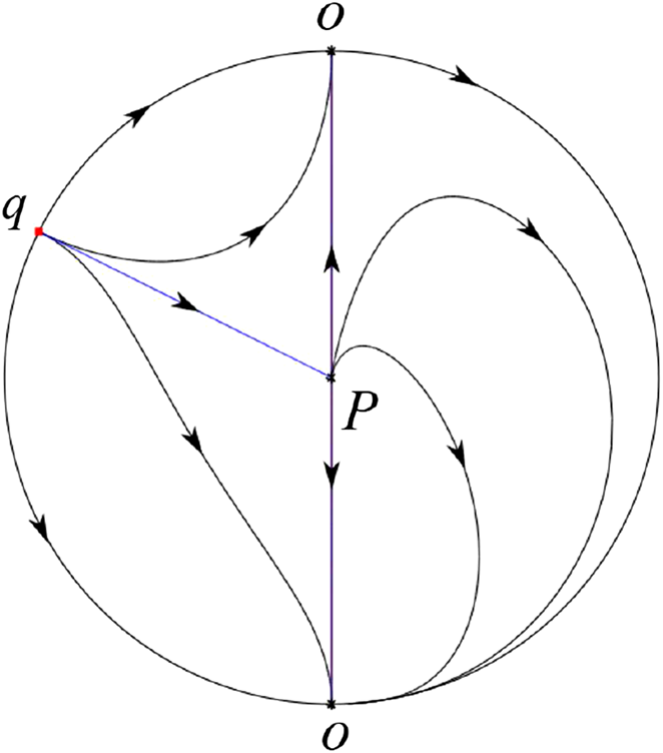
$$S=10,R=3$$

In the case (VI-7) one unstable separatrix of *P* goes to the saddle-node *O* in the positive *y*-direction and the second unstable separatrix of *P* goes to the stable node *O* in the negative *y*-direction. A stable separatrix of *P* comes from the unstable node *q*. On the other hand by the type of stability of the equilibrium points and by the Poincaré–Bendixson theorem we get the remaining orbits of the phase portrait. Thus the global phase portrait is shown in Fig. 66.

**Fig. 67:**
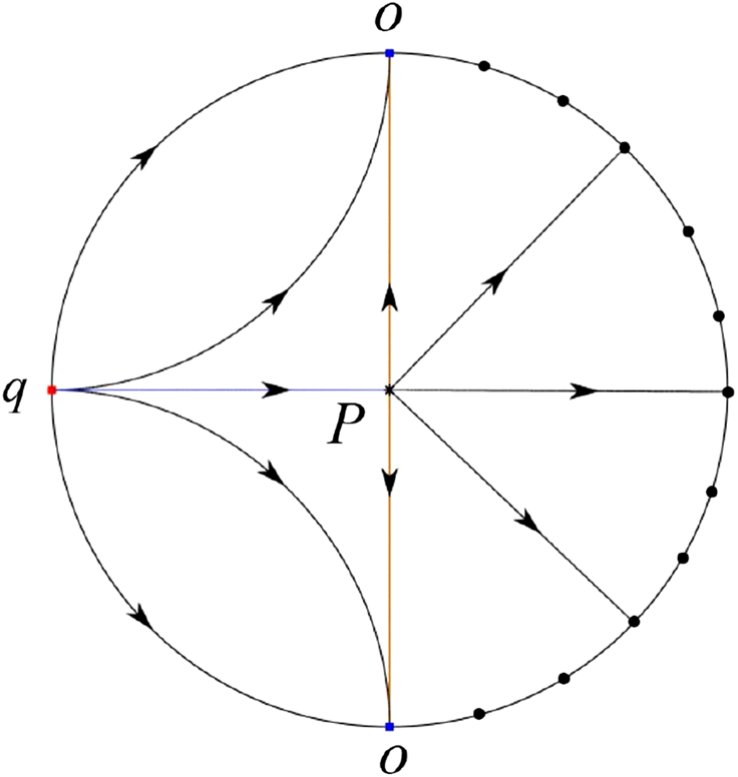
$$S=\infty$$

In the case (VI-8) one unstable separatrix of *P* goes to the stable node *O* in the positive *y*-direction of the half plane $x\le 0$ and the second unstable separatrix of *P* goes to the stable node *O* in the negative *y*-direction of the half plane $x\ge 0$. A stable separatrix of *P* comes from the unstable node *q*. The infinity of $x\ge 0$ is filled with equilibrium points. At each one of these infinite equilibrium points arrives an orbit starting at the unstable node *P* in the half plane $x\ge 0$. By the type of stability of the equilibrium points and by the Poincaré–Bendixson theorem we get the remaining orbits of the phase portrait. Thus the global phase portrait is shown in Fig. 67.

**Fig. 68:**
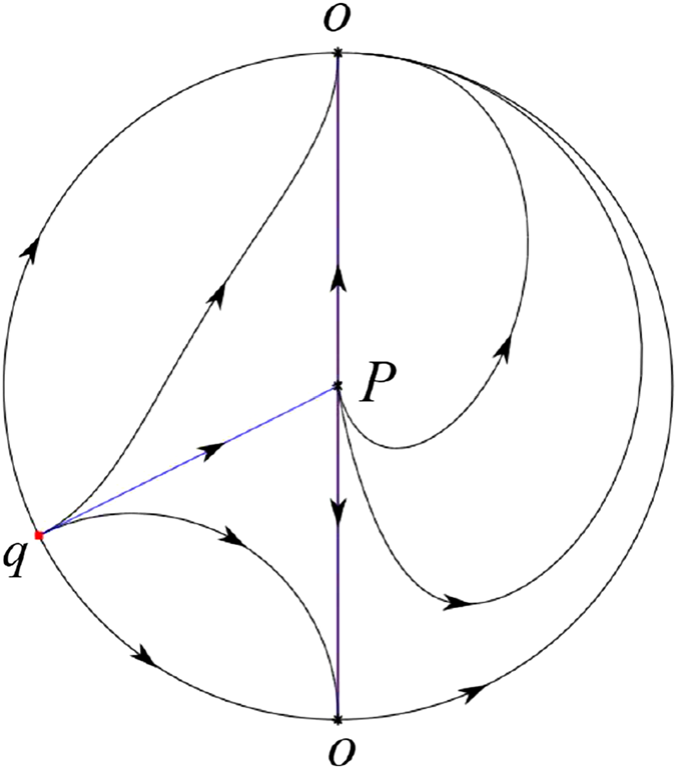
$$S=10,R=3$$

In the case (VI-9) a stable separatrix of *P* comes from the unstable node *q*. One unstable separatrix of *P* goes to the stable node *O* in the positive *y*-direction. The second unstable separatrix of *P* goes to the saddle-node *O* in the negative *y*-direction. By the type of stability of the equilibrium points and by the Poincaré–Bendixson theorem we get the remaining orbits of the phase portrait. Thus the global phase portrait is shown in Fig. 68.

**Fig. 69:**
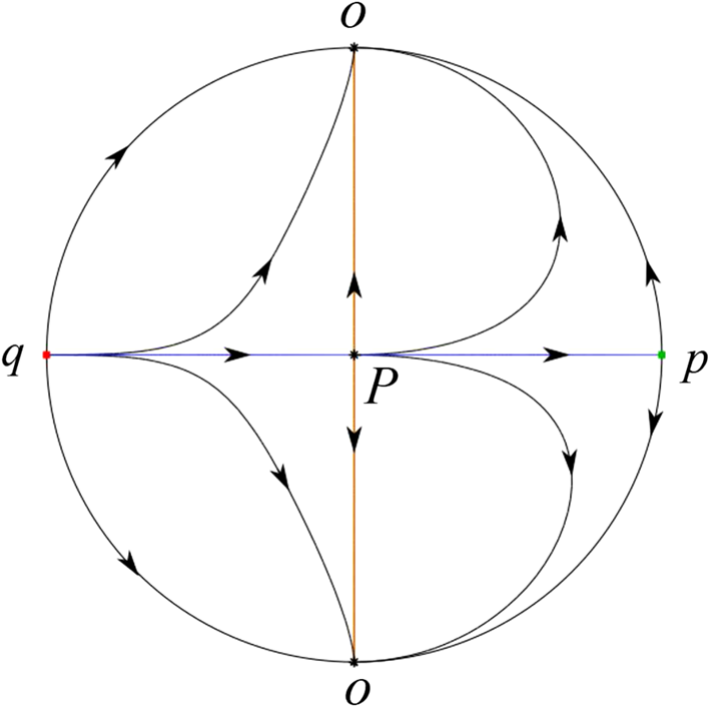
$$S=13,R=4$$

In the case (VI-10) a stable separatrix of *P* comes from the unstable node *q* in the half plane $x\le 0$. One unstable separatrix of *P* lying on the positive *y*-axis goes to the stable node *O* and the second unstable separatrix of *P* lying on the negative *y*-axis goes to the stable node *O* in the half plane $x\le 0$. The stable separatrix of the saddle *p* comes from *P*. We obtain the remaining orbits of the phase portraits by the type of stability of the equilibrium points and by the Poincaré–Bendixson theorem. Thus the global phase portrait is shown in Fig. 69.

## The Distinct Topologically Equivalent Phase Portraits

In this section we summarize the results on the distinct topological equivalent phase portraits from Figs. 17 to 69.

By the separatrix configuration of the phase portrait in Theorem 2 we have the following XIX categories

I. Figures 17, 29, 40 and 49 are topologically equivalent;
II. Figures 18 and 41 are topologically equivalent;
III. Figures 19 and 27 are topologically equivalent;
IV. Figures 20 and 26 are topologically equivalent;
V. Figures 21, 22, 24 and 25 are topologically equivalent;
VI. Figure 23;
VII. Figures 28, 43, 46 and 48 are topologically equivalent;
VIII. Figures 30, 34, 60 and 69 are topologically equivalent;
IX. Figures 31, 33, 61, 63, 66 and 68 are topologically equivalent;
X. Figure 32;
XI. Figures 35, 39, 50 and 59 are topologically equivalent;
XII. Figures 36, 38, 51, 53, 56 and 58 are topologically equivalent;
XIII. Figure 37;
XIV. Figures 42 and 47 are topologically equivalent;
XV. Figures 44 and 45 are topologically equivalent;
XVI. Figures 52 and 57 are topologically equivalent;
XVII. Figures 54 and 55 are topologically equivalent;
XVIII. Figures 62 and 67 are topologically equivalent;
XIX. Figures 64 and 65 are topologically equivalent.

This proves the first part of Theorem 1 and Fig. 1 comes from Figs. 17, 18, 19, 20, 21, 23, 28, 30, 31, 32, 35, 36, 37, 42, 44, 52, 54, 62, 64.

## Data Availability

The data underlying this article are available.
